# Transcriptomic regulation of the hypothalamic-pituitary axis by GnRH immunization in Xizang sheep

**DOI:** 10.1080/10495398.2026.2631819

**Published:** 2026-02-19

**Authors:** Tianzeng Song, Shehr Bano Mustafa, Haiyan Li, Xiaoming Zhang, Gaofu Wang, Tingting Zhang, Xiaoying Chen, Jianzhao Cui, Ming Zhang, Xianyin Zeng, Guiqiong Liu, Lili Xian, Zhuoma Jiayang, Wangsheng Zhao, Xunping Jiang

**Affiliations:** a College of Animal Science and Technology, Huazhong Agricultural University, Wuhan, China; b Institute of Animal Science, Xizang Academy of Agricultural and Animal Husbandry Science, Lhasa, Xizang, China; c College of Life Science and Agri-forestry, Southwest University of Science and Technology, Mianyang, China; d Dazhou City Animal Husbandry Technology Promotion Station, Dazhou, China; e Chongqing Academy of Animal Sciences, Rongchang, China; f Shigatse Science and Technology Bureau, Shigatse, China; g College of Animal Science and Technology, Sichuan Agricultural University, Chengdu, China; h College of Life Science, Sichuan Agricultural University, Ya’an, China; i Shigatse Agricultural and Animal Husbandry Science Research and Promotion Center, Shigatse, China

**Keywords:** Male Xizang sheep, Hypothalamus, Pituitary gland, Transcriptome sequence

## Abstract

Xizang sheep are vital economic livestock in plateau regions. Traditional surgical castration often induces stress and infection. Although immunization presents an alternative method, the physiological mechanisms underlying its effects in Xizang sheep remain unclear. Therefore, this study integrated serum immune-antioxidant indicators with hypothalamus-pituitary transcriptomics to investigate molecular mechanisms of GnRH immunization. Results indicated that serum IgA, IgG, IgM, SOD, and GSH in the immunization (IM) group were significantly higher than in control (CON) and surgical castration (SN) groups, while IL-6 and TNF-α were significantly reduced (*p* < 0.05). RNA-seq analysis revealed that hypothalamic *CYTB*, *ATP6*, *COX*, *ABLIM1*, and *ABI3* were significantly upregulated in IM group, whereas *SHISA7* and *PTPRO* were significantly downregulated, with notable enrichment in prion disease, oxidative phosphorylation, and thermogenesis pathways. Pituitary *RPL15* was upregulated while *RPL10A*, *CACNA1D*, *ANK1*, *DDX3X*, and *KCNMA1* were significantly downregulated, showing enrichment in myofibril, contractile fiber, sarcomere, and cytosolic ribosome pathways. Association analysis revealed significant positive correlations between IgG and pituitary *ATP6*, *CYTB*, *TNNI2*, as well as hypothalamic *COX1*, *COX*, and *ND4*. In summary, GnRH immunization outperforms surgical castration by modulating hypothalamic-pituitary genes and enhancing immunity and antioxidants in plateau Xizang sheep, achieving integrated neuroendocrine-immune regulation for healthy husbandry of plateau Xizang sheep.

## Introduction

Xizang sheep represent a unique breed that has adapted to the harsh conditions of the plateau environment. Their remarkable adaptability to high altitude, extreme climatic variations, and limited feed resources, they have become an important economic livestock breed in the region.^1^ Castration simplifies livestock and poultry management, accelerates fattening, and represents an important means of improving the economic efficiency of livestock farming.^2^ GnRH immunization has emerged as an alternative to surgical castration, altering the body’s hormonal balance by regulating the hypothalamic-pituitary-gonadal (HPG) axis. This process affects the growth performance of livestock and poultry while suppressing reproductive function.^3^ Therefore, elucidating the molecular regulatory mechanisms of GnRH immunization in Xizang sheep is essential for optimizing management strategies and enhancing economic benefits.

Changes in immune and antioxidant capacity following castration are critical indicators for assessing animal welfare. Serum immunoglobulins are key indicators for evaluating systemic humoral immune status, with IgG, IgM, and IgA playing significant roles in infection defense, immune homeostasis maintenance, and immune regulation.^4^ Increased pro-inflammatory cytokines (IL-6 and TNF-α) are indicators of impending disease, with higher serum and liver levels of IL-6 and TNF-α in inflammatory disease patients compared to healthy animals.^5^ The impacts of oxidative stress (OS) on animal growth and development, energy balance, metabolism, diet, production traits, and health status have been extensively studied.^6^^,^^7^ To meet the metabolic demands of various growth and development stages, animal oxidative metabolism is enhanced, leading to significantly elevated levels of reactive oxygen species (ROS) in organs and cells.^8^ Oxidative stress (OS) in ruminants is influenced by multiple factors and is closely associated with disease; therefore, enhancing antioxidant capacity is an important strategy for maintaining host health.^9^ Currently, the effects of GnRH immunization on immune and antioxidant functions in Xizang sheep remain unknown; therefore, exploring the potential role of GnRH immunization in immune and antioxidant regulation can provide theoretical support for optimizing the healthy breeding system of Xizang sheep in alpine pastoral areas.

The pituitary gland is hailed as the ‘commander of the endocrine system’ and constitutes an essential component of the mammalian endocrine and reproductive systems. Its secreted hormones govern metabolism, growth, and reproduction, making it a central target for investigating hormonal homeostasis changes following castration.^10^^,^^11^ Located in the sella turcica at the base of the skull, the pituitary comprises the adenohypophysis, which secretes multiple hormones including GH, FSH and LH, and the neurohypophysis, which stores and releases vasopressin and oxytocin synthesized by the hypothalamus.^12^^,^^13^ The hypothalamus is a highly conserved neural integration center that serves as both a maintainer of systemic homeostasis and a core regulator of immune responses and the autonomic nervous system.^14^ Studies have demonstrated that immunization can regulate the expression of genes related to lipid metabolism, antioxidant enzymes, and the tricarboxylic acid cycle in both the pituitary and hypothalamus.^15^ Transcriptome sequencing, through high-throughput capture and quantification of all tissue RNA, can systematically reveal gene regulatory networks and has become a powerful tool for dissecting the molecular mechanisms underlying important economic traits in livestock and poultry.^16^ Zhong et al.^17^ employed transcriptome sequencing to compare gene expression in the hypothalamic-pituitary-ovarian axis of Small-tailed Han sheep and Tan sheep across different estrous cycles, identifying *ODC1*, *PRLH*, *CRYBB2* and *SMAD5* as key differential genes in the estrogen signaling pathway. Lakhssassi et al.^18^ utilized transcriptomic sequencing technology to identify differentially expressed genes in the hypothalamus of rams exhibiting different sexual behaviors, pinpointing *PDYN*, *CGA*, *GABRD* and *TSHB* as core candidate molecules regulating sexual behavior. The molecular mechanisms underlying GnRH immunization effects on the pituitary-hypothalamic transcriptome in male Xizang sheep remain unexplored; transcriptomic analysis can systematically uncover its differential genes and pathways, providing a theoretical foundation for elucidating central regulatory mechanisms.

Taken together, this study preliminarily elucidated the molecular regulatory mechanisms of transcription factor targets in the HPG axis following GnRH immunization by integrating serum immunity and antioxidant indicators with hypothalamic-pituitary transcriptome analysis, providing a theoretical basis for optimizing precision castration techniques in plateau livestock.

## Materials and methods

### Ethics statement

We conformed protocols set by Livestock and Poultry Breeding Committee for the Ethical Review of Animal Well-being at the SWUST China. The whole method involving animals had been conducted in full agreement with ethical requirements for animal care (Approval No L2022026-2022-03). All owners gave their informed consent before the experimental procedure and sample collection.

### Preparation of the vaccine

GnRH vaccine was prepared following the method described in our earlier research,^19^^,^^20^ in which a synthetic variant of the gonadotrophin-releasing hormone decapeptide was modified by replacing the sixth amino acid with D-lysine to enable conjugation with ovalbumin (OVA). The peptide was purified using high-performance liquid chromatography, and Specol was employed as the adjuvant to emulsify the final vaccine formulation.

### Animal husbandry management and sample collection

Experimental animals were sourced from the Longri Livestock Farm in Hongyuan County, Aba Prefecture, Sichuan Province. Thirty male Xizang sheep at 6 months of age, with similar body weight, robust health, and sexual immaturity, were selected and randomly divided into three groups: control group (CON), surgical castration group (SN), and GnRH immunization group (IM), with 10 sheep in each group. All experimental sheep were vaccinated against diseases following standardized procedures, and each individual was systematically ear-tagged. The experimental period lasted 119 days, comprising a 7-day pre-experimental phase and a 112-day formal experimental phase. On the first day of the pre-experimental phase, sheep in the SN group underwent surgical castration. On day 1 of the feeding phase, 2 mL of GnRH emulsified antigen (containing 100 µg TDK) was subcutaneously injected into the neck region of sheep in the IM group. On day 56 of the feeding phase, a booster immunization was administered using the same dose of GnRH emulsified antigen as the initial immunization. The CON group received no treatment. Throughout the experimental period, all sheep had free access to water and were fed a standard diet twice daily at a rate of 0.5 kg per 30 kg body weight. From 08:00 to 18:00, all Xizang sheep were allowed to graze freely on pasture without supplemental feed provided. On the day before the end of the experiment, five sheep were randomly selected from each of the CON, SN, and IM groups and fasted for 12 h. Blood was then collected via jugular venipuncture, and serum was separated by centrifugation at 3000 r/min for 15 min. The sampled sheep were subsequently slaughtered for tissue collection. Pituitary and hypothalamus samples were collected, briefly rinsed in PBS, aliquoted into 2 mL enzyme-free cryovials, and immediately snap-frozen in liquid nitrogen. To ensure RNA integrity, all experimental samples were transported back to the laboratory after collection and stored at −80 °C for transcriptomic (RNA-Seq) and qRT-PCR analyses.

### Determination of immune indicators and antioxidant indicators

The collected serum was thawed in a 4 °C refrigerator. Immune indicators were measured using assay kits for immunoglobulin G (IgG), immunoglobulin M (IgM), immunoglobulin A (IgA), interleukin-6 (IL-6), and tumor necrosis factor-α (TNF-α). Antioxidant indicators were measured using kits for superoxide dismutase (SOD), malondialdehyde (MDA), total antioxidant capacity (T-AOC), and glutathione (GSH). All kits for immune and antioxidant indicators were purchased from Nanjing Jiancheng Bioengineering Institute, and all experimental procedures were performed according to the protocols provided with the kits.

### RNA extraction and quality assessment

RNA has been fully isolated from the tissue of pituitary gland and hypothalamus by TRIzol^®^ Reagent according to the manufacturer’s instructions (Invitrogen), and genomic DNA were removed with DNase I (TaKaRa). Subsequently, RNA quality was assessed utilizing the 2100 Bioanalyser (Agilent) and quantified with the ND-2000 (NanoDrop Technologies). The sequenced library had been made by exclusively RNA samples with high-quality (OD260/280 = 1.8–2.2, OD260/230 ≥ 2.0, RIN ≥ 6.5, 28S:18S ≥ 1.0, >2 μg).

### Construction and sequencing of RNA-seq library

A transcriptome library for RNA-seq was generated utilizing 1 μg of total RNA and the TruSeq™ RNA Sample Preparation Kit (Illumina, San Diego, CA, USA) by utilizing 1 μg of all RNA.). mRNA was isolated using poly(A) selection with oligo(dT) beads, followed by fragmentation with a specialized buffer. Subsequently, double-stranded cDNA had been produced by SuperScript double-stranded cDNA synthesis kit (Invitrogen, CA) with random hexamer primers (Illumina). Libraries were size-selected for cDNA target fragments measuring 200–300 bp on 2% Low Range Ultra Agarose, followed by PCR amplification utilizing Phusion DNA polymerase (NEB) for 15 cycles. Following TBS380 quantification, the paired-end RNA-seq library sequenced with the Illumina HiSeq xten/NovaSeq 6000 sequencer, including a read length of 2 × 150 bp.

### Differential gene screening and functional enrichment analysis

mRNA expression levels from four distinct libraries belonging to two groups were assessed using FPKM values obtained from Illumina sequencing data, and DEGs study had conducted employing the DESeq2 R v1.14.1 package. Benjamini-Hochberg (B-H) had been used *via* adjusting the *p*-value to control the false discovery rate (FDR). This study defined DEGs as those with FPKM > 1, adjusted *p*-value (*p*-adj) < 0.05, and |log2 fold change| ≥ 1. The analysis of differential gene expression clustering was conducted by heatmap R program. Bioinformatics analytical techniques have been utilized to conduct GO and KEGG pathway study on DEGs. Goatools program (v0.6.5) used for determine the principal GO functions linked to the gene collection. Fisher’s exact test employed. A GO function is considered substantially enriched *p*-value is <0.05. The R software had been utilized to study the KEGG study following same computational methodology as GO enrichment analysis.

### qRT-PCR analysis of DEGs

Eight differentially expressed genes (DEGs) were randomly selected and analyzed with qRT-PCR for the identical samples of RNA was obtained to sequencing to validate RNA-Seq results. Primer sequences for the genes are shown in Table 1. To synthesize cDNA, adhere to the guidelines included Quantscript RT Kit (TIANGEN BIOTECH CO., LTD, Beijing, China). RT-qPCR was conducted utilizing a CFX96 Touch™ real-time PCR detection system (Bio-Rad, Munich, Germany) and SYBR Green Super-Real Pre-Mix Plus (Tiangen Biotechnology Co., Ltd., Beijing, China). The 2 − ΔΔCt method was utilized to assess the data, and Sigmaplot v.8 had used for the Student’s *t*-test analyze the significance among samples.

**Table 1.** Primer sequences for hypothalamic-pituitary functional genes.

Gene	Forward primer	Temperature (°C)	Product (bp)
*ADPRHL1*	F: GCCTAACTGCTGCGTAAA	50.6	107
	R: TTGGAGGTCTGAGGTGTC	50.4	
*CHAT*	F: CCATCCTGCTCTGTATCG	49.4	110
	R: CCTGCCTGCTTCTATGTTA	49.6	
*EAF1*	F: TTGTGACCTCTTCTGTAGTG	50	108
	R: CAGTTAGCGGTTCTTGATTAG	50.4	
*FABP3*	F: GCATCAGTTCTTCACATCAG	50.2	100
	R: ATCCAACCAGTCCATCCTA	49.9	
*FGF1*	F: ACAATGCCTTGACTTCTCC	50.5	107
	R: CAACACCTGACAACCTGAT	50.4	
*LOC442994*	F: ACTGAGGAGGACCGTAAG	49.9	122
	R: GAGAGGTTGACATTGGATTG	49.7	
*PRTG*	F: TCATCTGTGGTCTCATCTTG	50.1	102
	R: AGGCACTGATACGAGGTAA	50.4	
*SCD*	F: CTACAAGAGTGGCTGAGTT	49.5	108
	R: GCATCCTGGTAGCATTATTC	49.6	

### Correlation analysis

Spearman correlation analysis was performed using Python’s SciPy library to calculate pairwise Spearman correlation coefficients and *p*-values between pituitary and hypothalamic DEGs and serum immune, antioxidant, and hormone levels. Multiple testing correction was conducted using the Benjamini-Hochberg method, with a significance threshold of FDR < 0.05. Correlation results were visualized as heatmaps using the Matplotlib library.

### Data processing and statistical analysis

Statistical analysis was performed using SPSS software (version 21.0) for one-way ANOVA, with independent samples *t*-test. Results were expressed as mean ± standard error of the mean (SEM). *p* < 0.05 was considered statistically significant, while *p* < 0.01 was considered highly significant. qRT-PCR results were calculated using the 2^−ΔΔCt method, with statistical significance determined by Student’s *t*-test or Mann-Whitney *U* test (*p* < 0.05). Graphs were generated using GraphPad Prism 5.0 software.

## Results

### Effects of GnRH immunization on immune and oxidative levels in Xizang sheep

Immune and antioxidant indicators are closely associated with animal health status. The effects of GnRH immunization on immune and oxidative levels in Xizang sheep are presented in Table 2. The IM group had the highest serum levels of IgA, IgG, IgM, SOD, and GSH, which were significantly higher than those in the CON group (*p* < 0.05), while IgG, IgM, SOD, and GSH levels were also significantly higher than in the SN group (*p* < 0.05). The SN group only showed significantly higher IgM content compared to the CON group (*p* < 0.05). In terms of inflammatory factors, the CON group had the highest IL-6 and TNF-α levels, significantly higher than both the SN and IM groups (*p* < 0.05), whereas the IM group had the lowest IL-6 level, significantly lower than the SN group (*p* < 0.05). No significant differences were found in T-AOC and MDA among the three groups (*p* > 0.05). These results indicate that GnRH immunization enhances immune function and free radical scavenging capacity, thereby alleviating oxidative stress.

**Table 2.** Effects of GnRH immunization on immune and antioxidant levels in Xizang sheep.

Index	CON	SN	IM	*p*-Value
IgA (mg/mL)	3.47 ± 0.40	2.97 ± 0.44	3.67 ± 0.42	0.055
IgG (mg/mL)	8.02 ± 1.01	8.28 ± 1.71	11.38 ± 1.03	0.002
IgM (mg/mL)	2.23 ± 3.98	2.53 ± 3.98	3.22 ± 3.98	<0.01
IL-6 (ng/L)	124.14 ± 5.79	104.43 ± 2.91	90.01 ± 6.68	<0.01
TNF-α (ng/L)	131.38 ± 9.53	87.39 ± 15.06	89.55 ± 13.89	<0.01
T-AOC (mmol/L)	1.32 ± 0.0	1.38 ± 0.06	1.31 ± 0.07	0.278
SOD (U/mL)	134.34 ± 1.90	145.11 ± 1.48	152.54 ± 2.13	<0.01
GSH (μmol/L)	18.11 ± 1.15	21.47 ± 1.20	26.74 ± 1.60	<0.01
MDA (nmol/mL)	6.11 ± 0.82	5.54 ± 0.79	6.07 ± 0.78	0.469

### Effects of GnRH immunization on pituitary gene expression

**Figure 1.**
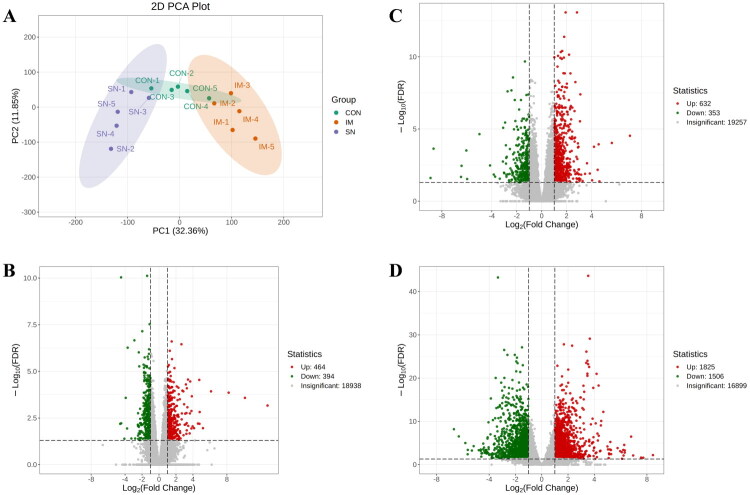
Analysis of DEGs in the pituitary. *Note:* (A) Principal component analysis of pituitary genes; (B) volcano plot of pituitary genes for IM *vs.* CON; (C) volcano plot of pituitary genes for SN *vs.* CON; (D) volcano plot of pituitary genes for IM *vs.* SN.

To investigate the effects of GnRH immunization on the pituitary transcriptome, transcriptome sequencing analysis was performed. Principal component analysis (PCA) revealed that the three sample groups formed distinct clusters, indicating significant differences in gene expression profiles (Fig. 1A). Volcano plot analysis showed that compared with the CON group, the IM group had 464 significantly upregulated and 394 significantly downregulated genes (Fig. 1B), while the SN group had 632 significantly upregulated and 353 significantly downregulated genes (Fig. 1C). When comparing IM *vs.* SN groups, the IM group exhibited 1,825 significantly upregulated and 1,506 significantly downregulated genes (Fig. 1D). These results demonstrate that GnRH immunization significantly reshaped the pituitary transcriptome.

### GO and KEGG enrichment analysis of pituitary DEGs

**Figure 2.**
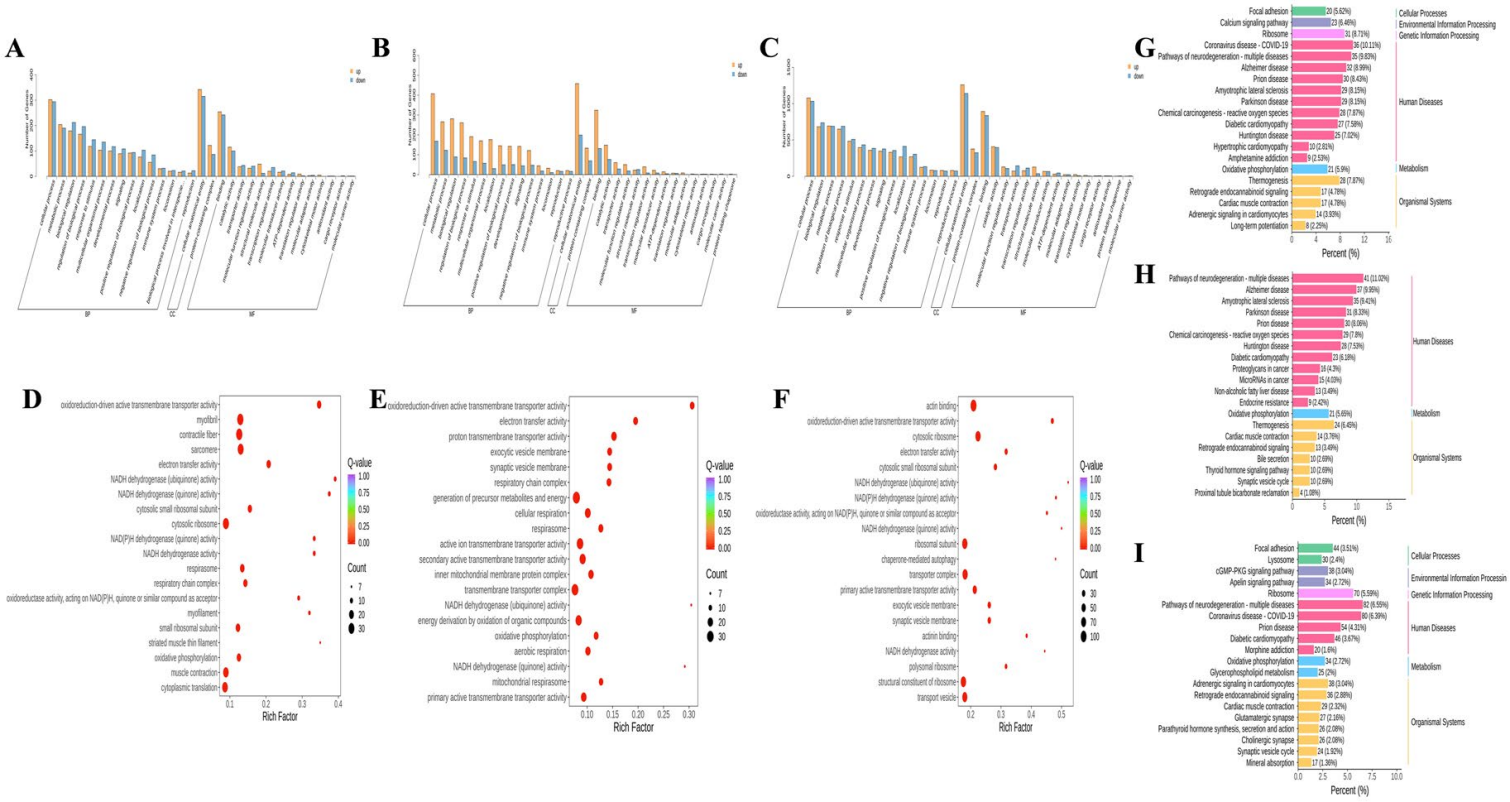
GO and KEGG enrichment analysis of pituitary DEGs. *Note:* (A) GO functional enrichment plot of differentially expressed genes in the pituitary between IM and CON; (B) GO functional enrichment plot of differentially expressed genes in the pituitary between SN and CON; (C) GO functional enrichment plot of differentially expressed genes in the pituitary between IM and SN; (D) scatter plot of GO enrichment analysis of differentially expressed genes in the pituitary between IM and CON; (E) scatter plot of GO enrichment analysis of differentially expressed genes in the pituitary between SN and CON; (F) scatter plot of GO enrichment analysis of differentially expressed genes in the pituitary between IM and SN; (G) KEGG pathway enrichment plot of differentially expressed genes in the pituitary between IM and CON; (H) KEGG pathway enrichment plot of differentially expressed genes in the pituitary between SN and CON; (I) KEGG pathway enrichment plot of differentially expressed genes in the pituitary between IM and SN.

To systematically dissect the impact of GnRH immunization on the pituitary transcriptional regulatory network in Xizang sheep, we performed comparative GO and KEGG functional enrichment analyses on differentially expressed genes (DEGs) among the CON, SN, and IM groups (Additional Table 1). GO enrichment analysis revealed that DEGs were primarily enriched across three domains: biological process, cellular component, and molecular function (Figs. 2A–C). Compared with CON, IM group DEGs were mainly enriched in pathways such as myofibril, contractile fiber, sarcomere, and cytosolic ribosome, with *RPL15* identified as a commonly upregulated gene in these pathways, while commonly downregulated genes included RPL10A, *CACNA1D*, and *ANK1* (Fig. 2D). In the SN group, DEGs were predominantly enriched in active ion transmembrane transporter activity, transmembrane transporter complex, and generation of precursor metabolites and energy pathways, with upregulated genes including *SLC25A22*, *ATP1A3*, and *ATP5B*, and commonly downregulated genes being *CYTB* and *COX1* (Fig. 2E). When comparing IM *vs.* SN, DEGs were primarily enriched in actin binding pathway, with significant enrichment also observed in cytosolic ribosome, transporter complex, and ribosomal subunit; commonly enriched genes such as *DDX3X*, *RPL10A*, and *KCNMA1* were significantly downregulated (Fig. 2F). KEGG functional enrichment analysis demonstrated that IM group DEGs were significantly enriched in Ribosome, Prion disease, and Oxidative Phosphorylation pathways compared with the other two groups (Figs. 2G,I); whereas SN *vs.* CON DEGs were significantly enriched in Thermogenesis and Oxidative Phosphorylation pathways (Fig. 2H).

### Effects of GnRH immunization on hypothalamic gene expression

**Figure 3.**
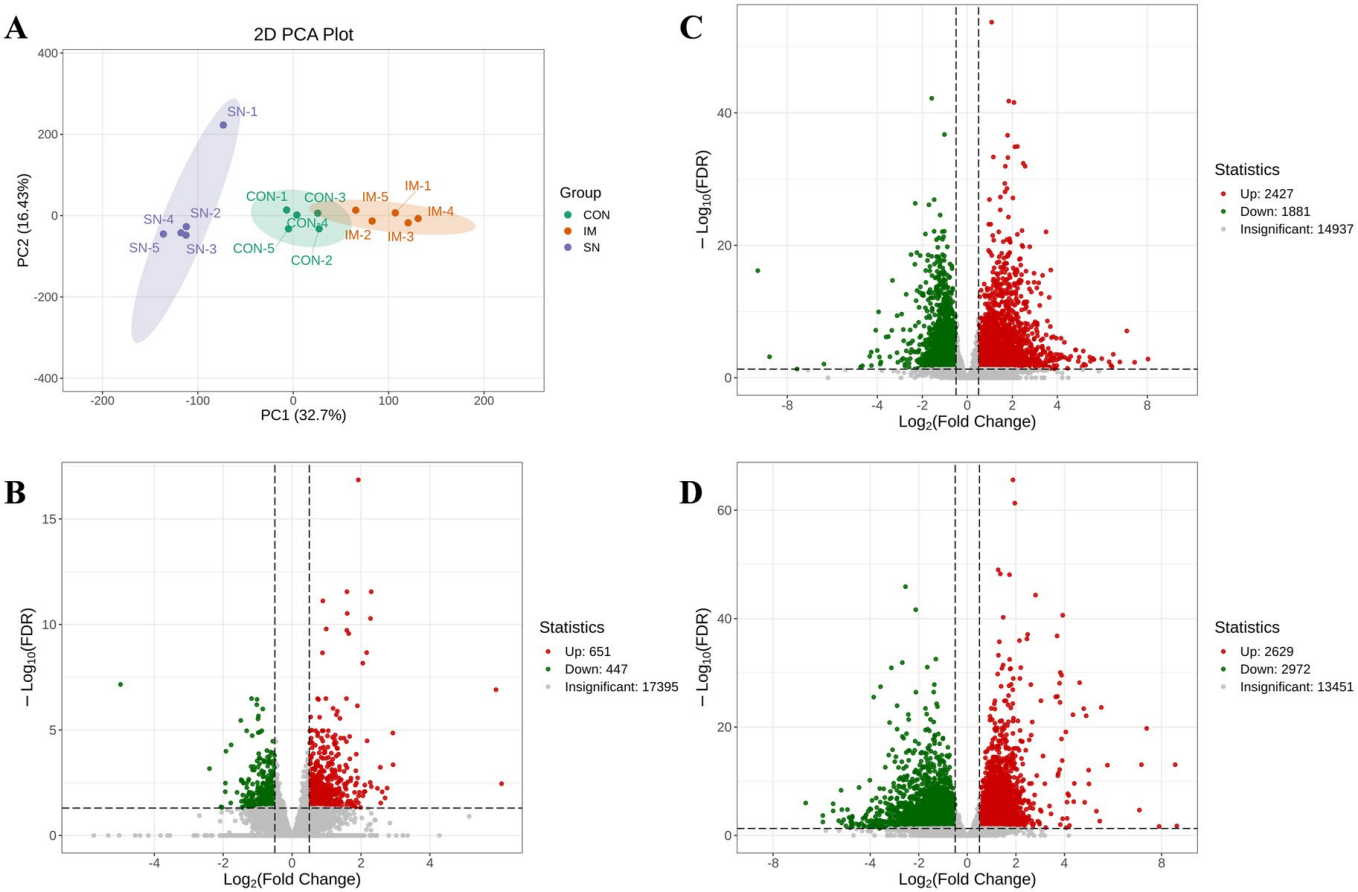
Analysis of DEGs in the hypothalamic. *Note:* (A) Principal component analysis of hypothalamic genes; (B) volcano plot of hypothalamic genes for IM *vs.* CON; (C) volcano plot of hypothalamic genes for SN *vs.* CON; (D) volcano plot of hypothalamic genes for IM *vs.* SN.

Differences in hypothalamic transcriptional profiles can further reveal the feedback regulation of the HPG axis by different castration methods and clarify the molecular mechanism by which GnRH immunization reshapes the reproductive center. Principal component analysis results showed that hypothalamic genes from the three groups clustered separately, indicating certain differences in gene expression among the three groups (Fig. 3A). Compared with the CON group, the IM group had 651 differential genes significantly upregulated and 447 differential genes significantly downregulated (Fig. 3B), while the SN group had 2427 differential genes significantly upregulated and 1881 genes significantly downregulated (Fig. 3C). When comparing IM with SN groups, the IM group had 2629 differential genes significantly upregulated and 2972 genes significantly downregulated (Fig. 3D).

### GO and KEGG enrichment analysis of hypothalamic DEGs

**Figure 4.**
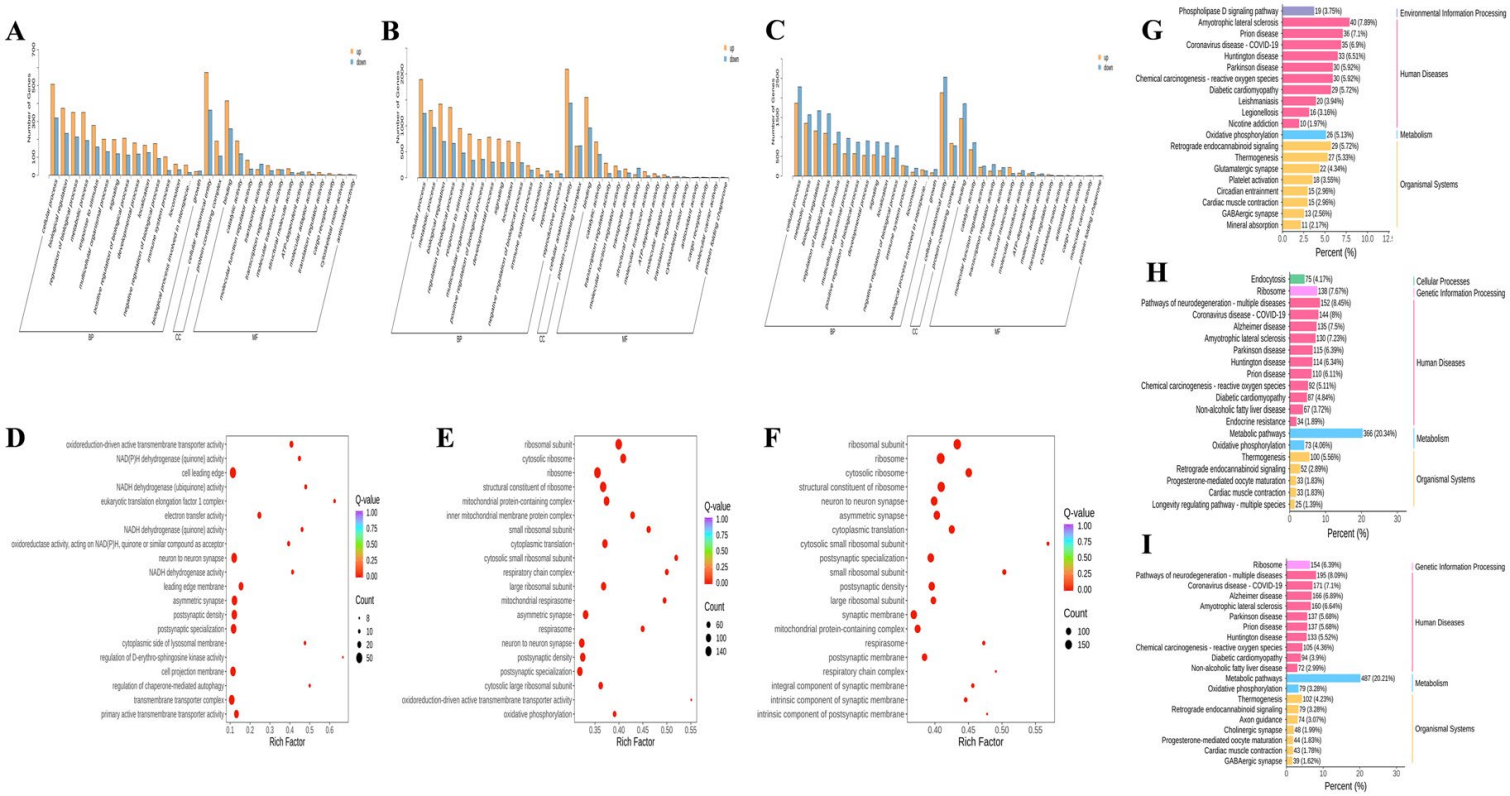
GO and KEGG enrichment analysis of hypothalamic DEGs. *Note:* (A) GO functional enrichment plot of differentially expressed genes in the hypothalamus between IM and CON; (B) GO functional enrichment plot of differentially expressed genes in the hypothalamus between SN and CON; (C) GO functional enrichment plot of differentially expressed genes in the hypothalamus between IM and SN; (D) scatter plot of GO enrichment analysis of differentially expressed genes in the hypothalamus between IM and CON; (E) scatter plot of GO enrichment analysis of differentially expressed genes in the hypothalamus between SN and CON; (F) scatter plot of GO enrichment analysis of differentially expressed genes in the hypothalamus between IM and SN; (G) KEGG pathway enrichment plot of differentially expressed genes in the hypothalamus between IM and CON; (H) KEGG pathway enrichment plot of differentially expressed genes in the hypothalamus between SN and CON; (I) KEGG pathway enrichment plot of differentially expressed genes in the hypothalamus between IM and SN.

To understand the unique transcriptional regulation pattern of GnRH immunization on the hypothalamus, we further analyzed GO and KEGG functional enrichment of hypothalamic differentially expressed genes (Additional Table 2). GO enrichment results showed that DEGs were mainly enriched in three ontologies: biological process, cellular component, and molecular function (Figs. 4A–C). Compared with the CON group, IM group DEGs were primarily enriched in Cell leading edge and Neuron to neuron synapse pathways, with commonly upregulated genes including *ABLIM1* and *ABI3*, and downregulated genes including *SHISA7* and *PTPRO* (Fig. 4D). In contrast, SN group DEGs were mainly enriched in ribosome, ribosomal subunit, and structural constituent of ribosome pathways, with upregulated genes including *MIEF1*, *DDX3X*, *MRPL15* and *NSUN4*, and downregulated genes including *NDUFA7*, *MRPL54*, *MRPS16* and the RPL and RPS gene families (Figs. 4E,F). KEGG functional enrichment revealed that IM group DEGs were significantly enriched in Amyotrophic lateral sclerosis, Prion disease, Oxidative phosphorylation, and Thermogenesis pathways compared with SN and CON groups, with upregulated genes including *CYTB*, *ATP6*, the *COX* gene family and the *ND* gene family, and downregulated gene *COX5A* (Figs. 4G,I). SN group DEGs were predominantly enriched in Metabolic pathways, with significant enrichment also observed in Ribosome, Thermogenesis, and Endocytosis (Fig. 4H).

### qRT-PCR validation of DEGs and correlation analysis

**Figure 5.**
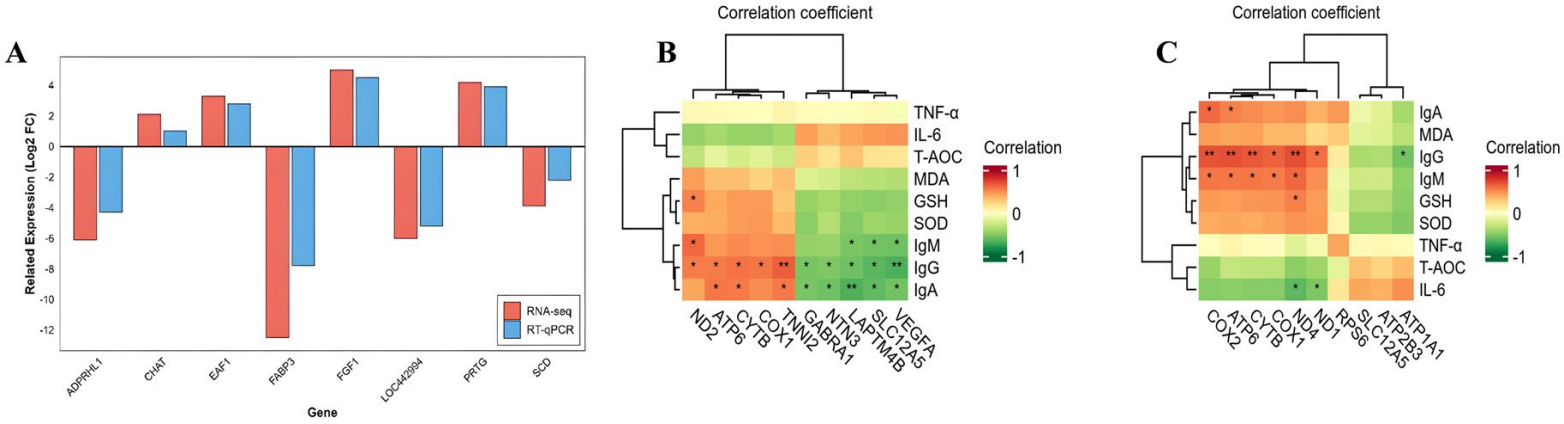
qRT-PCR validation and correlation analysis of pituitary and hypothalamic genes. *Note:* (A) qRT-PCR validation of pituitary and hypothalamic genes; (B,C) are correlation analysis heatmaps of pituitary and hypothalamic differentially expressed genes with immune and antioxidant indices, respectively.

This study selected eight key DEGs from the pituitary and hypothalamus for qRT-PCR validation, including upregulated *LOC442994* and *FABP3*, and downregulated *CHAT* and *SCD* in pituitary tissue; as well as upregulated *FGF1* and *EAF1,* and downregulated *ADPRHL1* and *PRTG* in hypothalamic tissue. The validation results were consistent with RNA-seq data, confirming the reliability of the sequencing results (Fig. 5A). To elucidate the coordinated regulatory relationships between pituitary-hypothalamic DEGs and reproductive hormone secretion, as well as immune-oxidative homeostasis, correlation analysis was performed between the top ten DEGs and immune and oxidative indicators. The results showed that in the pituitary, immune indicators such as IgA and IgG were significantly positively correlated with ATP6, CYTB and TNNI2, but significantly negatively correlated with *LAPTM4B*, *SLC12A5*, and *VEGFA* (Fig. 5B). In the hypothalamus, IgM and IgG were significantly positively correlated with *CYTB*, *ATP6*, *COX1*, *COX2* and *ND4*, while *ND1* and *ND4* were also significantly negatively correlated with IL-6 (Fig. 5C).

## Discussion

The hypothalamic-pituitary-gonadal (HPG) axis comprises the hypothalamus, anterior pituitary and gonads, representing a crucial neuroendocrine system responsible for regulating reproductive function and sex hormone production.^21^ This axis engages in close interaction with the immune system, achieving bidirectional regulation at both developmental and functional levels through hormones and cytokines.^22^ Increasing studies indicate that GnRH influences immune system development and function, and that ‘functional castration’ achieved through active GnRH immunization can preserve or enhance immune advantages.^23^^,^^24^ Oxidative stress serves as a core driving factor in disease pathogenesis and is associated with reduced reproductive hormone synthesis; it occurs when the generation of reactive oxygen species (ROS) generation exceeds their antioxidant clearance.^25^ This study demonstrates that GnRH castration can modulate hypothalamic-pituitary gene expression, thereby enhancing the immune and antioxidant capacity of Tibetan sheep, which is consistent with previous findings.^26^

IgA, IgG, and IgM are key immunoglobulins for disease resistance and immune regulation, while TNF-α and IL-6 mediate immune cell activation, B cell proliferation, and antibody responses, respectively.^22^^,^^27^^,^^28^ Excessive oxygen free radicals can induce oxidative stress. SOD, GSH, and T-AOC constitute the primary antioxidant defense system, while MDA is the end product of lipid peroxidation.^29^ GnRH castration may achieve immunomodulation through dual pathways: on one hand, the reduction of gonadal hormones can alleviate their inhibitory effect on B cell differentiation, promoting plasma cells to secrete immunoglobulins;^30^ on the other hand, the suppression of the hypothalamic-pituitary-gonadal axis may indirectly regulate oxidative stress pathways through the neuroendocrine network, manifested by significant increases in SOD and GSH activities.^^22^^,^^24^^ Previous studies reported that GnRH vaccination significantly elevated IgG and IgM antibodies in prostate cancer patients, demonstrating that GnRH vaccines can induce multiple types of humoral immune responses.^31^ Pan et al.^22^ found that GnRH immunization increased serum IL-6 levels in mice, proving that GnRH immunization enhances both humoral and cellular immune reactions, indirectly influencing immunoglobulin production. Han et al reported that GnRH immunization increased serum concentrations of IL-2, IL-4, IL-6 and TNF-α in rams, while also enhancing the expression of immune cytokines in the spleen.^32^ Additionally, castration affects the antioxidant capacity of pigs, with surgical castration demonstrating significantly higher antioxidant capacity compared to other castration methods.^33^ In this study, GnRH castration significantly enhanced immunoglobulin and antioxidant indicator levels while suppressing pro-inflammatory factor expression, revealing its immunoregulatory function in non-reproductive systems and providing theoretical support for GnRH-mediated immune intervention strategies.

The HPG axis is a crucial neuroendocrine system that regulates reproductive functions through hypothalamic GnRH hormone.^34^ Hypothalamic lncRNA can influence GnRH secretion, thereby regulating sheep reproductive performance.^35^ The pituitary gland serves as a bridge between the hypothalamus and gonads, influencing animal reproduction through the synthesis and secretion of FSH and LH.^36^ Yang et al. demonstrated that altered pituitary gene expression can regulate pituitary responses to steroid hormones and modulate sheep reproductive processes by affecting pituitary function.^37^ Studies indicate that GnRH analog drugs can lead to functional shutdown of the HPG axis through sustained suppression of the GnRH receptor signaling pathway.^38^ Transcriptomic research on goat hypothalamus revealed that GnRH neuronal activity is closely associated with the phased expression of multiple miRNAs, which participate in the initiation of sexual maturation by regulating signaling pathways such as JAK-STAT and PI3K-Akt.^39^ In this study, mitochondrial function-related genes such as ATP6 and CYTB were upregulated in the hypothalamus and pituitary, and showed positive correlations with IgG and IgM levels. These findings indicate that GnRH immunization maintains immune homeostasis by enhancing oxidative phosphorylation, thereby participating in the feedback regulation of the GnRH signaling pathway.^40^^,^^41^ The downregulation of *COX5A*, *SHISA7* and *PTPRO* disrupts mitochondrial complexes, leading to ROS imbalance and hypothalamic inflammatory microenvironment formation, thereby triggering GnRH network dysfunction and revealing the cross-talk mechanism of metabolic-immune-neural networks in GnRH castration.^41^^,^^42^ The significant enrichment of hypothalamic differential genes in pathways including Cell leading edge and Neuron-to-neuron synapse, Prion disease and amyotrophic lateral sclerosis, Oxidative phosphorylation, and Thermogenesis further demonstrates that GnRH immunization induces energy metabolic damage and calcium imbalance, ultimately leading to reproductive decline.^43–45^ A pituitary study reported that downregulation of *DDX3X* and *KCNMA1* genes reduces mRNA translation and impairs calcium-activated potassium channel function, leading to decreased excitability of gonadotropin cell membranes.^46^ Downregulation of *ANK1* in the pituitary further disrupts cytoskeletal integrity and membrane domain homeostasis in neuroendocrine neurons.^47^ In this study, genes such as *RPL10A*, *CACNA1D*, *ANK1*, *DDX3X* and *KCNMA1* were significantly downregulated, indicating that GnRH immunization disrupts the pituitary translation and secretion coupling mechanism by regulating the expression of ribosomal-related genes.^48^ The significant enrichment of differentially expressed genes in the hypothalamus and pituitary in pathways such as ‘Cell leading edge and Neuron to neuron synapse’, ‘Prion disease and amyotrophic lateral sclerosis’, ‘Oxidative phosphorylation’, and ‘Thermogenesis’ further indicates that GnRH active immunization integrates neuroendocrine and stress-immune responses by coordinately regulating pituitary energy metabolism, protein transport, and immune microenvironment, thereby influencing reproductive behavior and physiological processes.^43^^,^^44^

Surgical castration can directly eliminate gonadal negative feedback, triggering compensatory activation of the hypothalamic-pituitary axis.^49^ Previous transcriptome analysis revealed that surgical castration significantly upregulates the expression of reproductive-related neuropeptides in the hypothalamus of Tibetan sheep,^50^ whereas GnRH castration primarily affects G protein-coupled receptor signaling pathways and endoplasmic reticulum stress-related genes.^49^ Surgical castration eliminates gonadal hormone feedback, thereby removing the inhibition on the hypothalamus and pituitary gland, leading to increased pulse frequency of gonadotropin-releasing hormone (GnRH) and elevated levels of gonadotropins.^51^^,^^52^ These changes can further activate alternative signaling pathways in the absence of sex hormone receptors, such as in castration-resistant prostate cancer (CRPC), where amplification or mutation of the *AR* gene results in upregulation of compensatory pathways, including the glucocorticoid receptor.^53^ In this study, the mitochondrial-related genes *MIEF1*, *DDX3X*, *RPL* and *RPS* in the surgical group were primarily enriched in ribosomes, ribosomal subunits, and structural components of ribosomes. This suggests that surgical castration disrupts the mitochondrial and ribosomal functional networks in the hypothalamus-pituitary axis, leading to a reduction in overall cellular translation levels, thereby affecting hypothalamic metabolic remodeling and stress adaptation.^54^^,^^55^ The upregulation of *SLC25A22*, *ATP1A3* and *ATP5B* in the pituitary gland, along with the downregulation of *CYTB* and *COX1*, collectively reveals the critical role of mitochondrial energy metabolism in adaptive responses following surgical castration.^56^ The upregulation of *DDX3X* and *NSUN4* can inhibit tumor cell proliferation and enhance genomic stability. Their co-upregulation in the surgical castration group suggests that surgical castration may exert more durable anti-tumor effects through unique gene regulatory patterns.^57^^,^^58^

This study employed RNA-seq sequencing to investigate the transcriptional regulation of GnRH in the hypothalamus-pituitary axis of Tibetan sheep. Correlation analysis was preliminarily conducted to explore the relationship between transcription factors and immune/antioxidant functions. However, relevant functional validation remains lacking. Subsequent *in vitro* cell experiments could further verify the roles of genes such as *ATP6*, *CYTB* and *SHISA7* in immune-neuroendocrine crosstalk.

## Conclusion

This study systematically evaluated the effects of GnRH immunization on the hypothalamic-pituitary-gonadal (HPG) axis, immune function, and antioxidant capacity in Xizang sheep. The results showed that active GnRH immunization enhanced humoral immunity, improved the anti-inflammatory microenvironment, and elevated antioxidant capacity. At the molecular level, mitochondrial-encoded genes in the hypothalamus, including *ATP6*, *CYTB*, *COX* and the ND gene family, were upregulated and positively correlated with IgG and IgM, driving compensatory activation of energy metabolism to maintain immune homeostasis. DEGs were enriched in pathways such as Cell leading edge, Prion disease, and Amyotrophic lateral sclerosis, disrupting neuroendocrine balance and suppressing HPG axis function and gonadotropin synthesis. In the pituitary, RPL15 was upregulated while RPL10A, *CACNA1D*, *ANK1, DDX3X*, and *KCNMA1* were downregulated, with significant enrichment in Ribosome, Prion disease, and Oxidative Phosphorylation pathways, thereby disrupting the translation-secretion coupling in pituitary cells and leading to decreased excitability of gonadotropin cell membranes and vesicular transport disorders. *LAPTM4B*, *SLC12A5* and *VEGFA* were negatively correlated with IgA and IgG, indicating that GnRH immunization suppresses HPG axis function while preserving immunological advantage. In summary, GnRH immunization achieves integrated regulation of neuroendocrine and immune responses, providing theoretical support for efficient, low-stress castration technology in Xizang sheep and contributing to the development of healthy breeding practices.

## Supplementary Material

Additional Table2 GO and KEGG Pathway Enrichment Analysis of Hypothalamic Differentially Expressed Genes.docx

Additional Table1 GO and KEGG Pathway Enrichment Analysis of Pituitary Differentially Expressed Genes.docx

## Data Availability

All data from this study can be obtained by contacting the corresponding author upon request. This data can be found in the NCBI Sequence Read Archive (SRA) under accession number PRJNA1264702.
