# Supplementary material for: Transcriptomic regulation of the hypothalamic-pituitary axis by GnRH immunization in Xizang sheep
Source: Anim Biotechnol. 2026 Feb 19;37(1):2631819. doi: 10.1080/10495398.2026.2631819 (PMC12927403; doi:10.1080/10495398.2026.2631819)
Supplement: Additional Table2 GO and KEGG Pathway Enrichment Analysis of Hypothalamic Differentially Expressed Genes.docx [file LABT_A_2631819_SM2769.docx]

GO Enrichment Pathways and Differentially Expressed Genes in Hypothalamus (IM vs. CON)

| GO Enrichment Pathways | Up Genes | Down Genes |
| --- | --- | --- |
| ribosome | RPL7A;MRPL54;novel.3631;RPL36AL;MRPS26;RPL7;LOC101104725;RPS15;novel.900;novel.2495;RPL36A;RPL14;RPS10;MRPL41;RPL22;RPS13;novel.3376;novel.3935;RPL31;LOC101103316;RPL26;LOC105612165;RPS18;RPS9;LOC101123279;RPL35;MRPL13;RPL23;LOC101121371;MRPS2;RPS14;RPS27A;RPL18;RPL34;RPS4X;GNB2L1;RPS24;LOC101113344;RPL37RPL102;RPL4;LOC105604842;RPL9;MTG1;LOC101116132;LOC101115154;RPS25;RPL21;novel.37;RPL32;RPL19;RPSA;RPL28;EIF3H;LOC101102096;RPS11;novel.1438;RPS16;LOC101109155;RPS26;RPL3;RPL18A;RPS12;LOC101110998;RPL5;RPS23;RPS21;RPS7;RPS19;RPL13;RPL13A;MRPL27;LOC101123186;RPL24;LOC101123533;RPL12;RPL11;RPS20;RPL35A;RPL23A;RPLP1;MRPL14;RPLP2;RPL30;LOC443297;RPS27;LOC101109380;AURKAIP1;UBA52;RPLP0;novel.3330;novel.1181;LOC101102690;novel.283;CHCHD1;RPS17;RPS3;FAU;MRPL2;RPS6;MRPS35;RPL29;CMC1;MRPS34;LOC101104787;RPS29;MRPS14;LOC105607128;LOC101110941;LOC101118481;LOC101109545;RPL38;RPL39;MRPL24;LOC100037664;LOC105607745;MRPL36;RPS8;novel.574;LOC101114033;LOC101111215;RPL27;RPL36;RPS5;MRPL21;RPS2;RPL27A;novel.1132;SERP1;RPL10;RPS3A;MRPL32;MRPL10;LOC101103232;novel.2888;RPL8;LOC101110546;MRPL52;LOC101110758;MTERF4;MTG2;novel.256;RPL15;MRPS23;novel.2793;novel.1741;LOC101110176;BTF3;MRPS18A;RPS27L;LOC101107216;LOC101117120;LOC101123112;MRPL11;LOC101103595;LOC101107098;LOC105610137;LOC101122834;LOC101107031;LOC101105336;GCN1;RPL22L1;ISG15;MRPS33;RPL3L;MRPL28;LOC101111180;novel.3088;MRPL57;novel.2177;novel.459;NDUFA7;RPS262;LOC101104286;LOC106991949;LOC106990881;novel.1284;MRPS28;MRPL34;LOC101106855;MRPS16 | novel.407;DDX3X;LARP1;LARP4B;MRPS27;LOC106991948;NUFIP2;novel.2560;novel.2577;novel.2543;MIEF1;MRPL15;SF1;novel.606;novel.918;novel.3096;NSUN4;novel.1027 |
| ribosomal subunit | RPL7A;MRPL54;novel.3631;RPL36AL;MRPS26;RPL7;LOC101104725;RPS15;novel.900;novel.2495;RPL36A;RPL14;RPS10;MRPL41;RPS13;novel.3376;novel.3935;RPL31;LOC101103316;RPL26;LOC105612165;RPS18;RPS9;LOC101123279;RPL35;MRPL13;RPL23;LOC101121371;MRPS2;RPS14;RPS27A;RPL18;RPL34;RPS4X;GNB2L1;RPS24;LOC101113344;RPL37;RPL102;RPL4;LOC105604842;RPL9;LOC101116132;LOC101115154;RPS25;novel.37;RPL32;RPL19;RPSA;RPL28;LOC101102096;RPS11;novel.1438;RPS16;LOC101109155;RPS26;RPL3;RPL18A;RPS12;LOC101110998;RPL5;RPS23;RPS21;RPS7;RPS19;RPL13;RPL13A;MRPL27;LOC101123186;RPL24;LOC101123533;RPL12;RPL11;RPS20;RPL35A;RPL23A;RPLP1;MRPL14;RPLP2;RPL30;LOC443297;RPS27;LOC101109380;AURKAIP1;UBA52;RPLP0;novel.3330;novel.1181;LOC101102690;novel.283;CHCHD1;RPS3;FAU;MRPL2;RPS6;MRPS35;RPL29;CMC1;MRPS34;LOC101104787;RPS29;MRPS14;LOC105607128;LOC101118481;LOC101109545;RPL38;RPL39;MRPL24;LOC100037664;LOC105607745;MRPL36;RPS8;novel.574;LOC101114033;LOC101111215;RPL27;RPL36;RPS5;MRPL21;RPS2;RPL27A;novel.1132;RPL10;RPS3A;MRPL32;MRPL10;LOC101103232;novel.2888;RPL8;LOC101110546;MRPL52;LOC101110758;MTERF4;novel.256;RPL15;MRPS23;novel.2793;novel.1741;LOC101110176;MRPS18A;RPS27L;LOC101107216;LOC101117120;LOC101123112;MRPL11;LOC101103595;LOC101107098;LOC105610137;LOC101122834;LOC101105336;ISG15;MRPS33;RPL3L;MRPL28;LOC11111180;novel.3088;MRPL57;novel.2177;RPS262;LOC101104286;LOC106991949;LOC106990881;novel.1284;MRPS28;MRPL34;LOC101106855;MRPS16 | novel.407;DDX3X;MRPS27;LOC106991948;novel.2560;novel.2577;novel.2543;MIEF1;MRPL15;novel.918;NSUN4;novel.1027 |
| structural constituent of ribosome | MRPL54;novel.3631;RPL36AL;MRPS26;RPL7;LOC101104725;RPS15;novel.900;RPL36A;RPL14;RPS10;MRPL41;RPL22;RPS13;novel.3935;RPL31;LOC101103316;RPL26;LOC105612165;RPS18;RPS9;LOC101123279;RPL35;MRPL13;RPL23;LOC101121371;MRPS2;RPS14;RPS27A;RPL18;RPL34;RPS4X;RPS24;LOC101113344;RPL37;RPL102;RPL4;LOC105604842;RPL9;LOC101116132;LOC101115154;RPS25;RPL21;novel.37;RPL32;RPL19;RPSA;RPL28;LOC101102096;RPS11;novel.1438;RPS16;LOC101109155;RPS26;RPL3;RPL18A;RPS12;LOC101110998;RPL5;RPS23;RPS21;RPS7;RPS19;RPL13;RPL13A;MRPL27;LOC101123186;RPL24;LOC101123533;RPL12;RPL11;RPS20;RPL35A;RPL23A;RPLP1;MRPL14;RPLP2;RPL30;LOC443297;RPS27;LOC101109380;UBA52;RPLP0;novel.3330;novel.1181;LOC101102690;novel.283;RPS17;RPS3;FAU;MRPL2;RPS6;MRPS35;RPL29;CMC1;MRPS34;LOC101104787;RPS29;MRPS14;LOC105607128;LOC101110941;LOC101118481;LOC101109545;RPL38;RPL39;MRPL24;LOC100037664;LOC105607745;MRPL36;RPS8;novel.574;LOC101114033;RPL27;RPL36;RPS5;MRPL21;RPS2;RPL27A;novel.1132;RPL10;RPS3A;MRPL32;MRPL10;LOC101103232;novel.2888;RPL8;LOC101110546;MRPL52;LOC101110758;novel.256;RPL15;MRPS23;novel.2793;novel.1741;LOC101110176;MRPS18A;RPS27L;LOC101107216;LOC101117120;LOC101123112;MRPL11;LOC101103595;LOC101107098;LOC105610137;LOC101122834;LOC101105336;RPL22L1;ISG15;RPL3L;MRPL28;LOC101111180;novel.3088;MRPL57;novel.2177;novel.459;NDUFA7;RPS262;LOC101104286;LOC106991949;LOC106990881;novel.1284;MRPL34;LOC101106855;MRPS16 | novel.407;LOC106991948;novel.2560;novel.2577;novel.2543;MRPL15;novel.606;novel.918;novel.3096;novel.1027 |
| neuron to neuron synapse | RPS13;RPS18;RPS14;RPS25;novel.1438;ARFGAP1;HOMER3;RPS19;RPL12;KLHL17;RPL30;RPS27;LOC101109380;RPLP0;TSC2;DTNBP1;RPS3;ABHD17A;RGS9;RPL38;BAALC;AXIN1;RPL8;LOC101110546;LOC101110758;novel.256;ABI3;PDLIM5;FABP5;LYN;CAMK2N1;NGFR;GRASP;P2RX6;LOC101111180;LOC101106855 | NLGN2;PTPRS;ADD1;MPP2;EFNB2;PRR12;CACNG7;novel.3079;GRM7;SHISA9;ARFGEF2;ADAM22;CLSTN1;SLC8A2;SEMA4C;EFNB3;SYNGAP1;MAP1B;TSC1;CTNNB1;ADD2;BSN;SHISA7;ATP1A1;LOC105606122;DNAJC6;SLC4A8;SLITRK3;SHANK2;RTN4;LOC106990985;LRRTM3;DAGLA;PTPRO;RTN3;IQSEC1;CPEB3;BMPR2;PTCH1;DNM3;RAPGEF4;ATP2B2;DCC;SRGAP2;SORCS3;novel.2159;LRRTM2;SYT11;PCLO;CTNND1;ERBB4;GIT1;SYT7;SLC16A3;ATP1A3;GRID1;PTPRT;GRIN1;CLSTN2;NOS1;ARC;NLGN1;DCLK1;novel.2577;DLGAP2;SH3GL2;LOC101116422;AKAP9;SYN3;STXBP5;SYN2;PDPK1;SORCS2;ARHGEF9;IGSF9B;DLGAP1;SPTBN1;PLPPR4;PLEKHA5;GRIN2A;SPOCK1;FAM196A;ANKS1B;NLGN3;CPEB4;BACE1;ADCY1;LRRC4;LOC105607676;ARHGAP44;GPHN;LRRC7;KALRN;GRM3;LRFN5;CHRM2;LZTS1;LRFN4;TMEM108;CDH2;GRM1;GRIA2;LRRC4B;INPP4A;CACNG8;SYNPO;CAMK2A;SOS1;GOPC;EPHA4 |
| cytosolic ribosome | RPL7A;novel.3631;RPL36AL;RPL7;RPS15;novel.900;RPL36A;RPL14;RPS10;RPS13;novel.3376;RPL31;LOC101103316;RPL26;LOC105612165;RPS18;RPS9;LOC101123279;RPL35;RPL23;LOC101121371;RPS14;RPS27A;RPL18;RPL34;RPS4X;GNB2L1;RPS24;LOC101113344;RPL37;RPL102;RPL4;LOC105604842;RPL9;LOC101116132;LOC101115154;RPS25;novel.37;RPL32;RPL19;RPSA;RPL28;LOC101102096;RPS11;novel.1438;RPS16;LOC101109155;RPS26;RPL3;RPL18A;RPS12;LOC101110998;RPL5;RPS23;RPS21;RPS7;RPS19;RPL13;RPL13A;LOC101123186;RPL24;LOC101123533;RPL12;RPL11;RPS20;RPL35A;RPL23A;RPLP1;RPLP2;RPL30;LOC443297;RPS27;LOC101109380;UBA52;RPLP0;novel.3330;novel.1181;LOC101102690;novel.283;RPS3;FAU;RPS6;RPL29;CMC1;LOC101104787;RPS29;LOC105607128;LOC101118481;LOC101109545;RPL38;RPL39;LOC100037664;LOC105607745;RPS8;LOC101114033;LOC101111215;RPL27;RPL36;RPS5;RPS2;RPL27A;novel.1132;RPL10;RPS3A;LOC101103232;novel.2888;RPL8;LOC101110546;LOC101110758;novel.256;RPL15;novel.2793;LOC101110176;RPS27L;LOC101107216;LOC101117120;LOC101123112;LOC101103595;LOC101107098;LOC105610137;LOC101122834;LOC101105336;ISG15;RPL3L;LOC101111180;novel.3088;novel.2177;RPS262;LOC101104286;LOC106991949;LOC106990881;novel.1284;LOC101106855 | novel.407;DDX3X;LOC106991948;novel.2560;novel.2577;novel.2543;novel.918;novel.1027 |
| postsynaptic specialization | RPS13;RPS18;RPS14;RPS25;novel.1438;ARFGAP1;HOMER3;RPS19;RPL12;KLHL17;RPL30;RPS27;LOC101109380;RPLP0;TSC2;DTNBP1;RPS3;ABHD17A;RGS9;RPL38;BAALC;AXIN1;RPL8;LOC101110546;LOC101110758;novel.256;ABI3;PDLIM5;FABP5;LYN;CAMK2N1;NGFR;GRASP;CHRND;P2RX6;LOC101111180;LOC101106855 | NLGN2;PTPRS;ADD1;MPP2;EFNB2;PRR12;CACNG7;novel.3079;SHISA9;ADAM22;CLSTN1;SLC8A2;SEMA4C;EFNB3;SYNGAP1;MAP1B;TSC1;CTNNB1;ADD2;BSN;SHISA7;ATP1A1;LOC105606122;DNAJC6;SLITRK3;SHANK2;RTN4;LOC106990985;LRRTM3;DAGLA;PTPRO;RTN3;IQSEC1;CPEB3;BMPR2;PTCH1;DNM3;ATP2B2;DCC;SRGAP2;SORCS3;KPNA2;novel.2159;LRRTM2;SYT11;PCLO;CTNND1;ERBB4;GIT1;SLC16A3;GRID1;PTPRT;GRIN1;CHRNB2;CLSTN2;NOS1;ARC;NLGN1;DCLK1;novel.2577;DLGAP2;LOC101116422;AKAP9;GLRA4;SYN3;SYN2;PDPK1;SORCS2;ARHGEF9;IGSF9B;DLGAP1;SPTBN1;PLPPR4;PLEKHA5;GRIN2A;SPOCK1;FAM196A;ANKS1B;NLGN3;CPEB4;ADCY1;LRRC4;LOC105607676;ARHGAP44;GPHN;LRRTM1;LRRC7;KALRN;GRM3;LRFN5;LZTS1;LRFN4;TMEM108;CDH2;GRM1;GRIA2;LRRC4B;INPP4A;CACNG8;SYNPO;CAMK2A;SOS1;GOPC;EPHA4 |
| asymmetric synapse | RPS13;RPS18;RPS14;RPS25;novel.1438;ARFGAP1;HOMER3;RPS19;RPL12;KLHL17;RPL30;RPS27;LOC101109380;RPLP0;TSC2;DTNBP1;RPS3;ABHD17A;RGS9;RPL38;BAALC;AXIN1;RPL8;LOC101110546;LOC101110758;novel.256;ABI3;PDLIM5;FABP5;LYN;CAMK2N1;NGFR;GRASP;P2RX6;LOC101111180;LOC101106855 | PTPRS;ADD1;MPP2;EFNB2;PRR12;CACNG7;novel.3079;GRM7;SHISA9;ARFGEF2;ADAM22;CLSTN1;SLC8A2;SEMA4C;EFNB3;SYNGAP1;MAP1B;TSC1;CTNNB1;ADD2;BSN;SHISA7;ATP1A1;LOC105606122;DNAJC6;SLC4A8;SLITRK3;SHANK2;RTN4;LOC106990985;LRRTM3;DAGLA;PTPRO;RTN3;IQSEC1;CPEB3;BMPR2;PTCH1;DNM3;ATP2B2;DCC;SRGAP2;SORCS3;novel.2159;LRRTM2;SYT11;PCLO;CTNND1;ERBB4;GIT1;SLC16A3;GRID1;PTPRT;GRIN1;CLSTN2;NOS1;ARC;NLGN1;DCLK1;novel.2577;DLGAP2;LOC101116422;AKAP9;SYN3;SYN2;PDPK1;SORCS2;ARHGEF9;IGSF9B;DLGAP1;SPTBN1;PLPPR4;PLEKHA5;GRIN2A;SPOCK1;FAM196A;ANKS1B;NLGN3;CPEB4;ADCY1;LRRC4;LOC105607676;ARHGAP44;GPHN;LRRC7;KALRN;GRM3;LRFN5;CHRM2;LZTS1;LRFN4;TMEM108;CDH2;GRM1;GRIA2;LRRC4B;INPP4A;CACNG8;SYNPO;CAMK2A;SOS1;GOPC;EPHA4 |
| synaptic membrane | KCTD12;DTNBP1;ABHD17A;RGS9;COL13A1;SRPX2;novel.2103;STX4;GRID2IP;P2RX1;CHRNE;GRASP;CHRND;STX2;P2RX6;GABRE | SLC6A1;KCNJ9;CLCN3;NLGN2;CADM3;PTPRS;MPP2;EFNB2;CACNG7;GRM7;SYT1;SHISA9;ADAM22;CLSTN1;KCNA1;EPHB2;SEMA4C;EFNB3;CTNNB1;CSPG5;NRXN1;SHISA7;UNC13A;SLC4A8;SLITRK3;KCNMA1;LRRTM3;DAGLA;PTPRO;ZDHHC17;DNM3;KCNA3;ATP2B2;DCC;KCNC3;SRGAP2;SORCS3;KPNA2;novel.2159;LRRTM2;SYT11;PI4K2A;ERBB4;SYNJ1;ATP2B3;SYT7;ANK2;SLC16A3;ATP1A3;GRID1;CASK;PTPRT;GRIN1;CHRNB2;CLSTN2;ARC;NLGN1;GABRG1;LOC101116422;AKAP9;LHFPL4;GLRA3;CHRNA4;GABRR2;GLRA4;TRAPPC4;LOC105610339;KCNJ4;NIPSNAP1;SORCS2;GLRA1;GABRA4;KCNB1;CNTN2;KCNA2;IGSF9B;DLGAP1;LRFN3;PLPPR4;STX1B;CHRNA7;CLSTN3;GRIN2A;CNTNAP1;NLGN3;GABRR1;CNR1;CDH8;GAD2;FAIM2;ABHD6;ADCY1;LRRC4;NCSTN;LOC105607676;GRIK3;APBA1;GPHN;ATP2B1;LRRTM1;GABRA1;GRM3;LRFN5;CHRNA3;CHRM2;LZTS1;LRFN4;SYT6;CDH2;GRIA3;F2R;LRFN2;GRM1;RIMS1;GRIA2;LRRC4B;IL31RA;CACNG8;HIP1;EPHA4 |
| postsynaptic density | RPS13;RPS18;RPS14;RPS25;novel.1438;ARFGAP1;HOMER3;RPS19;RPL12;KLHL17;RPL30;RPS27;LOC101109380;RPLP0;TSC2;DTNBP1;RPS3;ABHD17A;RGS9;RPL38;BAALC;AXIN1;RPL8;LOC101110546;LOC101110758;novel.256;ABI3;PDLIM5;FABP5;LYN;CAMK2N1;NGFR;GRASP;P2RX6;LOC101111180;LOC101106855 | PTPRS;ADD1;MPP2;EFNB2;PRR12;CACNG7;novel.3079;SHISA9;ADAM22;CLSTN1;SLC8A2;SEMA4C;EFNB3;SYNGAP1;MAP1B;TSC1;CTNNB1;ADD2;BSN;SHISA7;ATP1A1;LOC105606122;DNAJC6;SLITRK3;SHANK2;RTN4;LOC106990985;LRRTM3;DAGLA;PTPRO;RTN3;IQSEC1;CPEB3;BMPR2;PTCH1;DNM3;ATP2B2;DCC;SRGAP2;SORCS3;novel.2159;LRRTM2;SYT11;PCLO;CTNND1;ERBB4;GIT1;SLC16A3;GRID1;PTPRT;GRIN1;CLSTN2;NOS1;ARC;NLGN1;DCLK1;novel.2577;DLGAP2;LOC101116422;AKAP9;SYN3;SYN2;PDPK1;SORCS2;ARHGEF9;IGSF9B;DLGAP1;SPTBN1;PLPPR4;PLEKHA5;GRIN2A;SPOCK1;FAM196A;ANKS1B;CPEB4;ADCY1;LRRC4;LOC105607676;ARHGAP44;GPHN;LRRC7;KALRN;GRM3;LRFN5;LZTS1;LRFN4;TMEM108;CDH2;GRM1;GRIA2;LRRC4B;INPP4A;CACNG8;SYNPO;CAMK2A;SOS1;GOPC;EPHA4 |
| cytoplasmic translation | novel.3631;RPL36AL;RPL36A;CNBP;RPS10;RPS13;novel.3376;RPL31;LOC101103316;RPL26;LOC105612165;RPS18;RPS9;LOC101123279;RPL23;LOC101121371;RPS14;RPS27A;RPL18;RPL34;RPS4X;GNB2L1;RPL37;LOC105604842;RPL9;LOC101116132;LOC101115154;RPS25;RPL32;RPL19;RPSA;EIF3H;LOC101102096;RPS11;novel.1438;RPS16;LOC101109155;RPS26;RPL18A;RPS12;LOC101110998;RPS23;RPS21;RPS7;RPS19;RPL13A;LOC101123186;RPL24;LOC101123533;RPL12;RPL11;RPS20;RPL35A;RPL23A;RPLP1;EIF3K;RPLP2;RPL30;LOC443297;RPS27;LOC101109380;UBA52;RPLP0;novel.3330;novel.1181;LOC101102690;RPS3;FAU;RPL29;CMC1;RPS29;LOC101109545;RPL38;RPL39;LOC100037664;EIF3E;LOC105607745;RPS8;LOC101114033;LOC101111215;RPL27;RPL36;RPS5;YBX1;RPL27A;novel.1132;RPS3A;LOC101103232;RPL8;LOC101110546;LOC101110758;PABPC1;novel.256;RPL15;novel.2793;EIF3M;HNRNPD;LOC101110176;LOC101107216;LOC101117120;LOC101123112;LOC101103595;LOC101107098;LOC105610137;LOC101122834;LOC101105336;RPL22L1;LOC101111180;novel.2177;RPS26-2;LOC101104286;LOC106991949;LOC106990881;novel.1284;LOC101106855 | DPH1;EIF4H;LOC101112694;CPEB3;FTSJ1;novel.2577;CPEB4;METTL3;novel.3302;CPEB2 |
| mitochondrial protein-containing complex | MRPL54;novel.3324;ATP5I;MRPS26;ND5;COX1;LOC101104725;COX3;ATP6;ND6;novel.368;novel.1573;novel.3901;novel.3902;novel.2495;LOC105606223;novel.370;MRPL41;LOC101105179;novel.371;NDUFA2;ROMO1;COX2;NDUFA13;UQCRH;MRPL13;MRPS2;ND2;NDUFB7;LOC101110916;IDH3G;ND4;ND1;NDUFS5;TIMM10;ATP5J2;MRPL27;NDUFA1;ATP8;novel.367;MRPL14;POLG;NDUFB10;AURKAIP1;LOC101108663;CHCHD1;ATP5J;MRPL2;MRPS35;TIMM8B;MRPS34;NDUFB11;MRPS14;NDUFA11;NDUFB9;MRPL24;novel.2849;NDUFB3;novel.2048;MRPL36;novel.574;novel.2617;CHCHD10;C5H19orf70;NDUFB1;ATP5L;MRPL21;LOC101121285;LOC101110664;MRPL32;LOC101108778;MRPL10;ND3;novel.2267;NDUFA3;MRPL52;LOC101119721;MTERF4;MRPS23;novel.1741;NDUFA6;novel.1122;TOMM5;BAX;SMDT1;NDUFS8;MRPS18A;LOC101121420;TIMM8A;MRPL11;MINOS1;novel.1318;FOXRED1;SUPV3L1;LOC101111410;LOC105609918;LOC101110066;LOC101115773;MRPS33;MRPL28;TRMT10B;MRPL57;MRPS36;NDUFA7;MRPS28;MRPL34;MRPS16 | MCCC2;MICU3;MCU;MRPS27;LOC101109981;ATP5B;MIEF1;SDHC;ATP5A1;MRPL15;SDHD;MTX3;TOMM70A;MTX2;NSUN4 |
| large ribosomal subunit | RPL7A;MRPL54;RPL36AL;RPL7;LOC101104725;novel.900;novel.2495;RPL36A;RPL14;MRPL41;RPL31;RPL26;LOC105612165;LOC101123279;RPL35;MRPL13;RPL23;LOC101121371;RPL18;RPL34;LOC101113344;RPL37;RPL102;RPL4;RPL9;LOC101115154;RPL32;RPL19;RPL28;LOC101109155;RPL3;RPL18A;LOC101110998;RPL5;RPL13;RPL13A;MRPL27;LOC101123186;RPL24;LOC101123533;RPL12;RPL11;RPL35A;RPL23A;RPLP1;MRPL14;RPLP2;RPL30;LOC101109380;UBA52;RPLP0;novel.3330;LOC101102690;MRPL2;RPL29;CMC1;LOC101104787;LOC105607128;LOC101118481;LOC101109545;RPL38;RPL39;MRPL24;LOC100037664;MRPL36;LOC101114033;RPL27;RPL36;MRPL21;RPL27A;novel.1132;RPL10;MRPL32;MRPL10;LOC101103232;novel.2888;RPL8;LOC101110546;MRPL52;MTERF4;RPL15;novel.2793;novel.1741;LOC101110176;LOC101123112;MRPL11;LOC101107098;LOC105610137;LOC101105336;RPL3L;MRPL28;LOC101111180;novel.3088;MRPL57;LOC101104286;LOC106990881;MRPL34;LOC101106855 | novel.407;LOC106991948;novel.2560;novel.2543;MIEF1;MRPL15;novel.918;NSUN4;novel.1027 |
| postsynaptic membrane | KCTD12;ABHD17A;RGS9;COL13A1;novel.2103;GRID2IP;P2RX1;CHRNE;GRASP;CHRND;P2RX6;GABRE | SLC6A1;CLCN3;NLGN2;PTPRS;MPP2;EFNB2;CACNG7;GRM7;SHISA9;ADAM22;CLSTN1;KCNA1;EPHB2;SEMA4C;EFNB3;CTNNB1;CSPG5;SHISA7;SLITRK3;KCNMA1;LRRTM3;DAGLA;PTPRO;DNM3;KCNA3;ATP2B2;DCC;KCNC3;SRGAP2;SORCS3;KPNA2;novel.2159;LRRTM2;ERBB4;ANK2;SLC16A3;GRID1;PTPRT;GRIN1;CHRNB2;CLSTN2;ARC;NLGN1;GABRG1;LOC101116422;AKAP9;LHFPL4;GLRA3;CHRNA4;GABRR2;GLRA4;TRAPPC4;LOC105610339;KCNJ4;SORCS2;GLRA1;GABRA4;KCNB1;CNTN2;IGSF9B;DLGAP1;LRFN3;PLPPR4;CHRNA7;CLSTN3;GRIN2A;NLGN3;GABRR1;FAIM2;ABHD6;ADCY1;LRRC4;LOC105607676;GRIK3;GPHN;LRRTM1;GABRA1;GRM3;LRFN5;CHRNA3;CHRM2;LZTS1;LRFN4;CDH2;GRIA3;F2R;LRFN2;GRM1;GRIA2;LRRC4B;CACNG8;HIP1;EPHA4 |
| small ribosomal subunit | novel.3631;MRPS26;RPS15;RPS10;RPS13;novel.3376;novel.3935;LOC101103316;RPS18;RPS9;MRPS2;RPS14;RPS27A;RPS4X;GNB2L1;RPS24;LOC105604842;LOC101116132;RPS25;novel.37;RPSA;LOC101102096;RPS11;novel.1438;RPS16;RPS26;RPS12;RPS23;RPS21;RPS7;RPS19;RPS20;LOC443297;RPS27;AURKAIP1;UBA52;novel.1181;novel.283;CHCHD1;RPS3;FAU;RPS6;MRPS35;MRPS34;RPS29;MRPS14;LOC105607745;RPS8;novel.574;LOC101111215;RPS5;RPS2;RPS3A;LOC101110758;novel.256;MRPS23;MRPS18A;RPS27L;LOC101107216;LOC101117120;LOC101103595;LOC101122834;ISG15;MRPS33;novel.2177;RPS262;LOC106991949;novel.1284;MRPS28;MRPS16 | DDX3X;MRPS27;novel.2577 |
| intrinsic component of synaptic membrane | P2RX1;CHRND;P2RX6 | SLC6A1;KCNJ9;CLCN3;NLGN2;CADM3;PTPRS;MPP2;EFNB2;CACNG7;SHISA9;ADAM22;KCNA1;EPHB2;EFNB3;CSPG5;SHISA7;SLITRK3;LRRTM3;DAGLA;PTPRO;KCNA3;ATP2B2;DCC;KCNC3;SORCS3;novel.2159;LRRTM2;ERBB4;ATP2B3;SYT7;SLC16A3;ATP1A3;GRID1;PTPRT;CHRNB2;CLSTN2;NLGN1;LOC101116422;GLRA4;LOC105610339;CNTN2;KCNA2;LRFN3;PLPPR4;GRIN2A;NLGN3;CNR1;ABHD6;ADCY1;LRRC4;NCSTN;GPHN;ATP2B1;LRRTM1;GRM3;LRFN5;CHRM2;LRFN4;SYT6;CDH2;GRM1;LRRC4B;EPHA4 |
| integral component of synaptic membrane | P2RX1;CHRND;P2RX6 | SLC6A1;KCNJ9;CLCN3;NLGN2;CADM3;PTPRS;EFNB2;CACNG7;SHISA9;ADAM22;KCNA1;EPHB2;EFNB3;CSPG5;SHISA7;SLITRK3;LRRTM3;DAGLA;PTPRO;KCNA3;ATP2B2;DCC;KCNC3;SORCS3;novel.2159;LRRTM2;ERBB4;ATP2B3;SYT7;SLC16A3;ATP1A3;GRID1;PTPRT;CHRNB2;CLSTN2;NLGN1;LOC101116422;GLRA4;LOC105610339;KCNA2;LRFN3;PLPPR4;GRIN2A;NLGN3;CNR1;ABHD6;ADCY1;LRRC4;NCSTN;ATP2B1;LRRTM1;GRM3;LRFN5;CHRM2;LRFN4;SYT6;CDH2;GRM1;LRRC4B;EPHA4 |
| respirasome | novel.3324;CYTB;ND5;COX1;COX3;ND6;novel.368;novel.1573;novel.3901;novel.3902;novel.3325;novel.370;LOC101105179;novel.371;NDUFA2;COX2;NDUFA13;UQCRH;novel.369;ND2;NDUFB7;ND4;ND1;NDUFS5;NDUFA1;novel.367;NDUFB10;LOC101108663;ND4L;LOC101118736;NDUFB11;NDUFA11;NDUFB9;LOC101116886;novel.2849;NDUFB3;novel.2048;NDUFB1;LOC101121285;LOC101110664;LOC101108778;ND3;NDUFA3;LOC101119721;NDUFA6;novel.1122;NDUFS8;LOC101121420;SURF1;FOXRED1;LOC105609918;LOC101121538;LOC101110066;LOC101115773;NDUFA7 | LOC101104348;C2H2orf69;SDHC;SDHD;HIGD1A;LOC101102527 |
| cytosolic small ribosomal subunit | novel.3631;RPS15;RPS10;RPS13;novel.3376;LOC101103316;RPS18;RPS9;RPS14;RPS27A;RPS4X;GNB2L1;RPS24;LOC105604842;LOC101116132;RPS25;novel.37;RPSA;LOC101102096;RPS11;novel.1438;RPS16;RPS26;RPS12;RPS23;RPS21;RPS7;RPS19;RPS20;LOC443297;RPS27;UBA52;novel.1181;novel.283;RPS3;FAU;RPS6;RPS29;LOC105607745;RPS8;LOC101111215;RPS5;RPS2;RPS3A;LOC101110758;novel.256;RPS27L;LOC101107216;LOC101117120;LOC101103595;LOC101122834;ISG15;novel.2177;RPS26-2;LOC106991949;novel.1284 | DDX3X;novel.2577 |
| respiratory chain complex | novel.3324;CYTB;ND5;COX1;COX3;ND6;novel.368;novel.1573;novel.3901;novel.3902;novel.3325;novel.370;LOC101105179;novel.371;NDUFA2;COX2;NDUFA13;UQCRH;novel.369;ND2;NDUFB7;ND4;ND1;NDUFS5;NDUFA1;novel.367;NDUFB10;LOC101108663;NDUFB11;NDUFA11;NDUFB9;LOC101116886;novel.2849;NDUFB3;novel.2048;NDUFB1;LOC101121285;LOC101110664;LOC101108778;ND3;NDUFA3;LOC101119721;NDUFA6;novel.1122;NDUFS8;LOC101121420;FOXRED1;LOC105609918;LOC101121538;LOC101110066;LOC101115773;NDUFA7 | SDHC;SDHD |
| intrinsic component of postsynaptic membrane | P2RX1;CHRND;P2RX6 | SLC6A1;CLCN3;NLGN2;PTPRS;MPP2;EFNB2;CACNG7;SHISA9;ADAM22;KCNA1;EPHB2;EFNB3;CSPG5;SHISA7;SLITRK3;LRRTM3;DAGLA;PTPRO;KCNA3;ATP2B2;DCC;KCNC3;SORCS3;novel.2159;LRRTM2;ERBB4;SLC16A3;GRID1;PTPRT;CHRNB2;CLSTN2;NLGN1;LOC101116422;GLRA4;LOC105610339;CNTN2;PLPPR4;GRIN2A;NLGN3;ABHD6;ADCY1;LRRC4;LRRTM1;GRM3;LRFN5;CHRM2;LRFN4;CDH2;GRM1;LRRC4B;EPHA4 |

GO Enrichment Pathways and Differentially Expressed Genes in Hypothalamus (IM vs. SN)

| GO Enrichment Pathways | Up Genes | Down Genes |
| --- | --- | --- |
| cell leading edge | EEF1A1;LOC101114018;FAM107A;AMOT;PSTPIP1;novel.2381;FGD2;AIF1;NF2;LOC101102178;PABPC1;DOCK8;LOC101110773;INPPL1;RAB22A;WIPF1;ABLIM1;LOC100125610;MCC;TLN1;APBB1IP;RAPGEF3;LOC101120236;CDC42BPG;FGD6;STX4;LOC101119576;PSD4;ARAP3;PLEK;LOC101102857;ABI3;S100B;NEDD9;MYO1G;novel.1720;HMHA1;novel.894;DDN;novel.423 | SLC12A5;ATP6V1B2;GABRA1;KCNC1;SHISA7;KCNC4;novel.2043;THY1;PTPRK;NHS;PTPRO;TRPM7;novel.1012;SNTG1 |
| neuron to neuron synapse | CTNND2;LYN;LOC101109380;IQSEC3;HOMER3;BCR;NCS1;IQSEC1;RPL12;novel.1438;NSMF;LOC105606096;ABLIM1;SHANK3;ARHGEF9;ADD1;GRIP2;ABI3;MAGI2;NGFR | ATP1A3;GRIN2A;PTPRS;LOC101114066;LRFN5;GRM7;NEFH;MAP1B;SHISA7;GRIA4;PCLO;RTN3;GAP43;GRIA2;ATP1A1;STXBP5;SORCS3;novel.589;PTPRO;DNM3;GRIN1;SLITRK1;ABHD17B;ITPR1 |
| transmembrane transporter complex | COX1;CYTB;novel.3324;novel.371;ND5;novel.370;novel.368;ND6;ND4;LOC101107658;ND2;ND1;TEX40;novel.1573;ND3;SLC26A6 | ATP1A3;GRIN2A;SCN8A;GABRA1;KCNC1;UNC80;GRIA4;DPP6;KCNB1;GABRA3;KCNC4;CACNA2D1;GRIA2;VWC2;GABRG2;CHRNA7;ATP1A1;SCN1A;CACNA2D2;KCNA3;STXBP5;ABHD6;novel.589;TRPC1;KCNA1;GRIN1;HCN3;CACNA1E |
| asymmetric synapse | CTNND2;LYN;LOC101109380;IQSEC3;HOMER3;BCR;NCS1;IQSEC1;RPL12;novel.1438;NSMF;LOC105606096;ABLIM1;SHANK3;ARHGEF9;ADD1;GRIP2;ABI3;MAGI2;NGFR | GRIN2A;PTPRS;LOC101114066;LRFN5;GRM7;NEFH;MAP1B;SHISA7;GRIA4;PCLO;RTN3;GAP43;GRIA2;ATP1A1;SORCS3;novel.589;PTPRO;DNM3;GRIN1;SLITRK1;ABHD17B;ITPR1 |
| postsynaptic specialization | CTNND2;LYN;LOC101109380;IQSEC3;HOMER3;BCR;NCS1;IQSEC1;RPL12;novel.1438;NSMF;LOC105606096;ABLIM1;SHANK3;ARHGEF9;ADD1;GRIP2;ABI3;MAGI2;NGFR | GRIN2A;PTPRS;LOC101114066;LRFN5;NEFH;MAP1B;SHISA7;GRIA4;PCLO;RTN3;GAP43;GRIA2;ATP1A1;SORCS3;novel.589;PTPRO;DNM3;GRIN1;LRRTM1;SLITRK1;ABHD17B;ITPR1 |
| postsynaptic density | CTNND2;LYN;LOC101109380;IQSEC3;HOMER3;BCR;NCS1;IQSEC1;RPL12;novel.1438;NSMF;LOC105606096;ABLIM1;SHANK3;ARHGEF9;ADD1;GRIP2;ABI3;MAGI2;NGFR | GRIN2A;PTPRS;LOC101114066;LRFN5;NEFH;MAP1B;SHISA7;GRIA4;PCLO;RTN3;GAP43;GRIA2;ATP1A1;SORCS3;novel.589;PTPRO;DNM3;GRIN1;SLITRK1;ABHD17B;ITPR1 |
| cell projection membrane | EEF1A1;LOC101114018;FAM107A;GNA12;novel.2381;FGD2;AIF1;NF2;LOC101102178;DOCK8;LOC101110773;LOC100125610;TLN1;SHANK3;MSN;LOC101120236;STX4;LOC101119576;LOC101102230;MAPRE1;PSD4;PLEK;LOC101102857;SLC26A6;novel.1720;GNA13;HMHA1;DDN;novel.423 | SLC12A5;GABRA1;KCNC1;SHISA7;ADCY3;KCNC4;NDRG4;THY1;GAP43;TMEM67;BBS7;SNTG1 |
| leading edge membrane | EEF1A1;LOC101114018;FAM107A;novel.2381;FGD2;AIF1;NF2;DOCK8;LOC101110773;LOC100125610;TLN1;LOC101120236;STX4;LOC101119576;PSD4;PLEK;LOC101102857;MYO1G;novel.1720;HMHA1;DDN;novel.423 | SLC12A5;GABRA1;KCNC1;SHISA7;KCNC4;THY1;PTPRK;SNTG1 |
| primary active transmembrane transporter activity | COX2;COX1;CYTB;novel.3901;novel.3324;novel.371;novel.369;ND5;COX3;novel.370;novel.368;ND6;ND4;ND2;ND1;ND4L;novel.1573;ND3 | ATP1A3;ATP6V1B2;ATP2B3;ATP6V0A1;LOC101104348;ABCG4;ATP6V0C;ATP6V0D1;ATP1A1;ATP6V1H;ATP13A5;LOC101120446 |
| oxidoreduction-driven active transmembrane transporter activity | COX2;COX1;CYTB;novel.3901;novel.3324;novel.371;novel.369;ND5;COX3;novel.370;novel.368;ND6;ND4;ND2;ND1;ND4L;novel.1573;ND3 | LOC101104348;LOC101120446 |
| electron transfer activity | COX2;COX1;CYTB;novel.3901;novel.3324;novel.371;novel.369;ND5;COX3;novel.370;novel.368;ND6;ND4;ND2;ND1;LOC101106452;ND4L;novel.1573;ND3 | LOC101104348 |
| NAD(P)H dehydrogenase (quinone) activity | novel.3324;novel.371;ND5;novel.370;ND6;ND4;ND2;ND1;ND4L;novel.1573;ND3;CBR4 | LOC101104348 |
| oxidoreductase activity, acting on NAD(P)H, quinone or similar compound as acceptor | novel.3324;novel.371;ND5;novel.370;ND6;ND4;ND2;ND1;ND4L;novel.1573;ND3;CBR4 | LOC101104348 |
| NADH dehydrogenase (ubiquinone) activity | novel.3324;novel.371;ND5;novel.370;ND6;ND4;ND2;ND1;ND4L;novel.1573;ND3 | LOC101104348 |
| NADH dehydrogenase (quinone) activity | novel.3324;novel.371;ND5;novel.370;ND6;ND4;ND2;ND1;ND4L;novel.1573;ND3 | LOC101104348 |
| NADH dehydrogenase activity | novel.3324;novel.371;ND5;novel.370;ND6;ND4;ND2;ND1;ND4L;novel.1573;ND3 | LOC101104348 |
| eukaryotic translation elongation factor 1 complex | EEF1A1;LOC101114018;novel.2381;EEF1D;LOC101110773;LOC100125610;LOC101120236;LOC101102857;novel.423 | EEF1A2 |
| cytoplasmic side of lysosomal membrane | EEF1A1;LOC101114018;novel.2381;LOC101110773;LOC100125610;LOC101120236;LITAF;LOC101102857;novel.423 | EEF1A2 |
| regulation of chaperone-mediated autophagy | EEF1A1;LOC101114018;novel.2381;LOC101110773;LOC100125610;LOC101120236;LOC101102857;novel.423 | EEF1A2 |
| regulation of D-erythro-sphingosine kinase activity | EEF1A1;LOC101114018;novel.2381;LOC101110773;LOC100125610;LOC101120236;LOC101102857;novel.423 | -- |

GO Enrichment Pathways and Differentially Expressed Genes in Hypothalamus (SN vs. CON)

| GO Enrichment Pathways | Up Genes | Down Genes |
| --- | --- | --- |
| ribosome | LARP4B;LARP1;NUFIP2;novel.407;MIEF1;LOC106991948;DDX3X;MRPL15;DHX29;LOC101119765;novel.2543;NSUN4;novel.918 | RPL36AL;novel.3935;RPL7A;novel.3631;RPL7;LOC101104725;LOC105612165;RPL36;MRPL41;MTG1;MRPS26;MRPL54;novel.3376;RPS15;novel.2495;LOC101102690;RPS10;novel.900;RPS13;RPL36A;MRPL13;AURKAIP1;RPL14;LOC101103316;RPS18;MRPL36;LOC101102096;RPL22;RPL26;MRPS2;RPL23;LOC101123279;RPL19;CHCHD1;RPS14;RPS9;LOC101121371;RPL31;LOC443297;RPS16;RPL37;RPS4X;RPL35;RPL35A;RPL18;MRPL14;RPL21;RPS27A;LOC101113344;RPL3;RPS21;LOC101115154;RPL13;RPL18A;RPS19;MRPL27;RPL24;LOC101110998;MRPL24;RPS26;novel.37;MRPL2;MRPL52;novel.688;RPS23;RPLP2;RPL9;RPL34;RPS25;LOC105610139;RPL28;RPS12;RPL102;MRPS34;novel.1181;MRPS14;RPL13A;LOC105604842;LOC101123533;RPS24;RPL32;MRPL21;LOC105607128;RPS7;RPLP1;RPS11;RPL10;MRPS23;RPL11;CMC1;RPL30;MRPL11;RPL38;RPL4;RPS17;RPL12;UBA52;LOC101123186;RPS3;FAU;novel.1438;RPS20;RPL23A;RPS29;MRPL47;MRPS18A;LOC101109155;MRPS35;RPL5;novel.574;LOC101111215;RPL29;novel.2003;novel.3330;novel.283;novel.2327;RPL26L1;RPSA;LOC101111180;RPS27;LOC101118057;RPLP0;LOC101116132;novel.256;RPL39;LOC101110546;LOC101114033;LOC101109380;MRPL55;LOC101104787;ICT1;RPS8;LOC101123112;RPL27;RPS3A;LOC105607745;novel.459;RPS6;MRPL32;novel.1741;ISG15;RPS2;MRPL57;novel.120;MRPS15;LOC101103595;novel.1132;MRPS28;RPL6;RPL8;RPL27A;RPS5;RPS262;novel.3088;RPS27L;LOC101110758;novel.2888;LOC101119869;LOC101109545;MRPS33 |
| ribosomal subunit | novel.407;MIEF1;LOC106991948;DDX3X;MRPL15;DHX29;LOC101119765;novel.2543;NSUN4;novel.918 | RPL36AL;novel.3935;RPL7A;novel.3631;RPL7;LOC101104725;LOC105612165;RPL36;MRPL41;MRPS26;MRPL54;novel.3376;RPS15;novel.2495;LOC101102690;RPS10;novel.900;RPS13;RPL36A;MRPL13;AURKAIP1;RPL14;LOC101103316;RPS18;MRPL36;LOC101102096;RPL26;MRPS2;RPL23;LOC101123279;RPL19;CHCHD1;RPS14;RPS9;LOC101121371;RPL31;LOC443297;RPS16;RPL37;RPS4X;RPL35;RPL35A;RPL18;MRPL14;RPS27A;LOC101113344;RPL3;RPS21;LOC101115154;RPL13;RPL18A;RPS19;MRPL27;RPL24;LOC101110998;MRPL24;RPS26;novel.37;MRPL2;MRPL52;novel.688;RPS23;RPLP2;RPL9;RPL34;RPS25;LOC105610139;RPL28;RPS12;RPL102;MRPS34;novel.1181;MRPS14;RPL13A;LOC105604842;LOC101123533;RPS24;RPL32;MRPL21;LOC105607128;RPS7;RPLP1;RPS11;RPL10;MRPS23;RPL11;CMC1;RPL30;MRPL11;RPL38;RPL4;RPL12;UBA52;LOC101123186;RPS3;FAU;novel.1438;RPS20;RPL23A;RPS29;MRPL47;MRPS18A;LOC101109155;MRPS35;RPL5;novel.574;LOC101111215;RPL29;novel.2003;novel.3330;novel.283;novel.2327;RPL26L1;RPSA;LOC101111180;RPS27;LOC101118057;RPLP0;LOC101116132;novel.256;RPL39;LOC101110546;LOC101114033;LOC101109380;MRPL55;LOC101104787;ICT1;RPS8;LOC101123112;RPL27;RPS3A;LOC105607745;RPS6;MRPL32;novel.1741;ISG15;RPS2;MRPL57;novel.120;MRPS15;LOC101103595;novel.1132;MRPS28;RPL6;RPL8;RPL27A;RPS5;RPS26-2;novel.3088;RPS27L;LOC101110758;novel.2888;LOC101119869;LOC101109545;MRPS33 |
| structural constituent of ribosome | novel.407;LOC106991948;MRPL15;LOC101119765;novel.2543;novel.918 | RPL36AL;novel.3935;novel.3631;RPL7;LOC101104725;LOC105612165;RPL36;MRPL41;MRPS26;MRPL54;RPS15;LOC101102690;RPS10;novel.900;RPS13;RPL36A;MRPL13;RPL14;LOC101103316;RPS18;MRPL36;LOC101102096;RPL22;RPL26;MRPS2;RPL23;LOC101123279;RPL19;RPS14;RPS9;LOC101121371;RPL31;LOC443297;RPS16;RPL37;RPS4X;RPL35;RPL35A;RPL18;MRPL14;RPL21;RPS27A;LOC101113344;RPL3;RPS21;LOC101115154;RPL13;RPL18A;RPS19;MRPL27;RPL24;LOC101110998;MRPL24;RPS26;novel.37;MRPL2;MRPL52;novel.688;RPS23;RPLP2;RPL9;RPL34;RPS25;LOC105610139;RPL28;RPS12;RPL10-2;MRPS34;novel.1181;MRPS14;RPL13A;LOC105604842;LOC101123533;RPS24;RPL32;MRPL21;LOC105607128;RPS7;RPLP1;RPS11;RPL10;MRPS23;RPL11;CMC1;RPL30;MRPL11;RPL38;RPL4;RPS17;RPL12;UBA52;LOC101123186;RPS3;FAU;novel.1438;RPS20;RPL23A;RPS29;MRPL47;MRPS18A;LOC101109155;MRPS35;RPL5;novel.574;RPL29;novel.2003;novel.3330;novel.283;novel.2327;RPL26L1;RPSA;LOC101111180;RPS27;LOC101118057;RPLP0;LOC101116132;novel.256;RPL39;LOC101110546;LOC101114033;LOC101109380;MRPL55;LOC101104787;RPS8;LOC101123112;RPL27;RPS3A;LOC105607745;novel.459;RPS6;MRPL32;novel.1741;ISG15;RPS2;MRPL57;novel.120;MRPS15;LOC101103595;novel.1132;RPL6;RPL8;RPL27A;RPS5;RPS26-2;novel.3088;RPS27L;LOC101110758;novel.2888;LOC101119869;LOC101109545 |
| cytosolic ribosome | novel.407;LOC106991948;DDX3X;DHX29;novel.2543;novel.918 | RPL36AL;RPL7A;novel.3631;RPL7;LOC105612165;RPL36;novel.3376;RPS15;LOC101102690;RPS10;novel.900;RPS13;RPL36A;RPL14;LOC101103316;RPS18;LOC101102096;RPL26;RPL23;LOC101123279;RPL19;RPS14;RPS9;LOC101121371;RPL31;LOC443297;RPS16;RPL37;RPS4X;RPL35;RPL35A;RPL18;RPS27A;LOC101113344;RPL3;RPS21;LOC101115154;RPL13;RPL18A;RPS19;RPL24;LOC101110998;RPS26;novel.37;novel.688;RPS23;RPLP2;RPL9;RPL34;RPS25;LOC105610139;RPL28;RPS12;RPL10-2;novel.1181;RPL13A;LOC105604842;LOC101123533;RPS24;RPL32;LOC105607128;RPS7;RPLP1;RPS11;RPL10;RPL11;CMC1;RPL30;RPL38;RPL4;RPL12;UBA52;LOC101123186;RPS3;FAU;novel.1438;RPS20;RPL23A;RPS29;LOC101109155;RPL5;LOC101111215;RPL29;novel.2003;novel.3330;novel.283;novel.2327;RPL26L1;RPSA;LOC101111180;RPS27;LOC101118057;RPLP0;LOC101116132;novel.256;RPL39;LOC101110546;LOC101114033;LOC101109380;LOC101104787;RPS8;LOC101123112;RPL27;RPS3A;LOC105607745;RPS6;ISG15;RPS2;novel.120;LOC101103595;novel.1132;RPL6;RPL8;RPL27A;RPS5;RPS26-2;novel.3088;RPS27L;LOC101110758;novel.2888;LOC101119869;LOC101109545 |
| mitochondrial protein-containing complex | MICU3;LOC101109981;MIEF1;MTX3;LOC105616860;MRPL15;ATP5A1;SDHD;MCCC2;LOC101119765;TOMM40L;NSUN4;ATP5B | ND6;ND5;ATP5I;LOC101104725;novel.3324;MRPL41;NDUFA2;NDUFA13;MRPS26;novel.368;LOC105606223;MRPL54;NDUFB7;novel.2495;NDUFS5;LOC101105179;COX3;POLG;COX1;ROMO1;ATP6;LOC101110916;MRPL13;novel.1573;AURKAIP1;TIMM10;NDUFB3;MRPL36;UQCRH;MRPS2;CHCHD1;TIMM8B;novel.370;ATP8;NDUFB1;ATP5J2;NDUFA3;NDUFB10;novel.371;NDUFA8;LOC101115773;MRPL14;novel.3901;novel.3902;ATP5J;MRPL27;NDUFB9;MRPL24;DNAJC19;MRPL2;MRPL52;NDUFA1;MRPS34;MRPS14;MRPL21;LOC101108778;LOC101108663;novel.3368;MRPS23;LOC101111410;NDUFA11;MRPL11;novel.2849;ND2;novel.2617;novel.1122;NDUFB11;LOC101110664;BAX;LOC101119721;MRPL47;MRPS18A;MRPS35;novel.574;novel.2048;LOC105604454;novel.1423;COX2;TOMM5;NDUFS7;LOC101110066;ND1;LOC101121420;SMDT1;MRPL55;LOC105609918;ATP5H;ICT1;CHCHD10;novel.2267;NDUFS8;C5H19orf70;novel.551;ATP5L;MRPL32;novel.1741;MRPS36;NDUFB8;MRPL57;ND4;LOC101121285;MRPS15;MRPS28;novel.309;LOC101116871;MINOS1;LOC101121949;MRPS33;LOC101102327 |
| neuron to neuron synapse | IQSEC1;ADD1;SHISA9;EFNB2;TSC1;BMPR2;PRR12;LOC105606122;CTNND2;LOC106990985;SRGAP2;NLGN2;MPP2;BSN;SORCS2;ARHGEF9;DCLK1;ADD2;PTPRS;SHANK2;CTNNB1;CACNG7;CPEB3;ADAM22;ARFGEF2;SLC8A2;ERBB4;NCS1;EFNB3;PDPK1;GRM7;GIT1;SYNGAP1;DCC;SEMA4C;novel.2159;ABLIM1;DAGLA;LOC105612524;ARC;FAM196A;ATP2B2;IQSEC3;DNAJC6;ATP7A;LOC105606096;NOS1;SPTBN1;CHRM2;SH3GL2;GRM5;CLSTN1;CLSTN2;MAGI2;PTCH1;DLGAP1;DLGAP2;INPP4A;PTK2B;LRRTM3;SLITRK3;LRRTM2;IGSF9B;DISC1;RTN4;GRIN1;NLGN1;novel.2975;SYT7;ATP1A1;PTPRO;SHISA7;PTPRT;AKAP9;ERC1;SYN2;SYN3;SLC16A3;SLC4A8;PSD3;SIPA1L1;LRRC4;CTNND1;PPP1R9B;ARHGAP44;LOC101116422;BCR;GRID1;SORCS3;SYT11;SHANK1;MAP1B | RPS13;RPS18;RPS14;KLHL17;RPS19;DTNBP1;novel.688;RPS25;RPL30;RPL38;RPL12;RPS3;novel.1438;RGS9;LOC101111180;RPS27;LOC101118057;RPLP0;novel.256;LOC101110546;LOC101109380;LOC105609538;HOMER3;RPL8;FABP5;LOC101110758 |
| asymmetric synapse | IQSEC1;ADD1;SHISA9;EFNB2;TSC1;BMPR2;PRR12;LOC105606122;CTNND2;LOC106990985;SRGAP2;MPP2;BSN;SORCS2;ARHGEF9;DCLK1;ADD2;PTPRS;SHANK2;CTNNB1;CACNG7;CPEB3;ADAM22;ARFGEF2;SLC8A2;ERBB4;NCS1;EFNB3;PDPK1;GRM7;GIT1;SYNGAP1;DCC;SEMA4C;novel.2159;ABLIM1;DAGLA;LOC105612524;ARC;FAM196A;ATP2B2;IQSEC3;DNAJC6;ATP7A;LOC105606096;NOS1;SPTBN1;CHRM2;GRM5;CLSTN1;CLSTN2;MAGI2;PTCH1;DLGAP1;DLGAP2;INPP4A;PTK2B;LRRTM3;SLITRK3;LRRTM2;IGSF9B;DISC1;RTN4;GRIN1;NLGN1;novel.2975;ATP1A1;PTPRO;SHISA7;PTPRT;AKAP9;ERC1;SYN2;SYN3;SLC16A3;SLC4A8;PSD3;SIPA1L1;LRRC4;CTNND1;PPP1R9B;ARHGAP44;LOC101116422;BCR;GRID1;SORCS3;SYT11;SHANK1;MAP1B | RPS13;RPS18;RPS14;KLHL17;RPS19;DTNBP1;novel.688;RPS25;RPL30;RPL38;RPL12;RPS3;novel.1438;RGS9;LOC101111180;RPS27;LOC101118057;RPLP0;novel.256;LOC101110546;LOC101109380;HOMER3;RPL8;FABP5;LOC101110758 |
| postsynaptic specialization | IQSEC1;ADD1;SHISA9;EFNB2;TSC1;BMPR2;PRR12;LOC105606122;CTNND2;LOC106990985;SRGAP2;NLGN2;MPP2;BSN;SORCS2;ARHGEF9;DCLK1;ADD2;PTPRS;SHANK2;CTNNB1;CACNG7;CPEB3;ADAM22;SLC8A2;ERBB4;NCS1;EFNB3;KPNA2;PDPK1;GIT1;SYNGAP1;DCC;SEMA4C;novel.2159;ABLIM1;DAGLA;LOC105612524;ARC;FAM196A;ATP2B2;IQSEC3;DNAJC6;ATP7A;LOC105606096;NOS1;SPTBN1;CHRNB2;GRM5;CLSTN1;CLSTN2;MAGI2;PTCH1;DLGAP1;DLGAP2;INPP4A;PTK2B;LRRTM3;SLITRK3;LRRTM2;IGSF9B;DISC1;RTN4;GRIN1;NLGN1;novel.2975;ATP1A1;PTPRO;SHISA7;PTPRT;AKAP9;ERC1;SYN2;SYN3;SLC16A3;PSD3;SIPA1L1;LRRC4;CTNND1;PPP1R9B;ARHGAP44;LOC101116422;BCR;GRID1;SORCS3;SYT11;SHANK1;MAP1B;GLRA4 | RPS13;RPS18;RPS14;KLHL17;RPS19;DTNBP1;novel.688;RPS25;RPL30;RPL38;RPL12;RPS3;novel.1438;RGS9;LOC101111180;RPS27;LOC101118057;RPLP0;novel.256;LOC101110546;LOC101109380;HOMER3;RPL8;FABP5;LOC101110758 |
| postsynaptic density | IQSEC1;ADD1;SHISA9;EFNB2;TSC1;BMPR2;PRR12;LOC105606122;CTNND2;LOC106990985;SRGAP2;MPP2;BSN;SORCS2;ARHGEF9;DCLK1;ADD2;PTPRS;SHANK2;CTNNB1;CACNG7;CPEB3;ADAM22;SLC8A2;ERBB4;NCS1;EFNB3;PDPK1;GIT1;SYNGAP1;DCC;SEMA4C;novel.2159;ABLIM1;DAGLA;LOC105612524;ARC;FAM196A;ATP2B2;IQSEC3;DNAJC6;ATP7A;LOC105606096;NOS1;SPTBN1;GRM5;CLSTN1;CLSTN2;MAGI2;PTCH1;DLGAP1;DLGAP2;INPP4A;PTK2B;LRRTM3;SLITRK3;LRRTM2;IGSF9B;DISC1;RTN4;GRIN1;NLGN1;novel.2975;ATP1A1;PTPRO;SHISA7;PTPRT;AKAP9;ERC1;SYN2;SYN3;SLC16A3;PSD3;SIPA1L1;LRRC4;CTNND1;PPP1R9B;ARHGAP44;LOC101116422;BCR;GRID1;SORCS3;SYT11;SHANK1;MAP1B | RPS13;RPS18;RPS14;KLHL17;RPS19;DTNBP1;novel.688;RPS25;RPL30;RPL38;RPL12;RPS3;novel.1438;RGS9;LOC101111180;RPS27;LOC101118057;RPLP0;novel.256;LOC101110546;LOC101109380;HOMER3;RPL8;FABP5;LOC101110758 |
| cytoplasmic translation | LOC101112694;CPEB3;EIF4B;EIF4H;FTSJ1;DHX29;DPH1;ZNF385A | RPL36AL;novel.3631;LOC105612165;RPL36;novel.3376;LOC101102690;RPS10;CNBP;RPS13;RPL36A;LOC101103316;RPS18;LOC101102096;RPL26;RPL23;LOC101123279;RPL19;RPS14;RPS9;LOC101121371;RPL31;LOC443297;RPS16;RPL37;RPS4X;RPL35A;RPL18;RPS27A;RPS21;LOC101115154;RPL18A;RPS19;RPL24;LOC101110998;RPS26;novel.688;RPS23;RPLP2;RPL9;RPL34;RPS25;LOC105610139;RPS12;EIF3K;novel.1181;RPL13A;LOC105604842;LOC101123533;RPL32;LOC101122488;RPS7;RPLP1;RPS11;RPL11;CMC1;RPL30;RPL38;RPL12;UBA52;LOC101123186;RPS3;FAU;novel.1438;RPS20;RPL23A;RPS29;LOC101109155;LOC101111215;RPL29;novel.3330;novel.2327;RPL26L1;RPSA;LOC101111180;RPS27;LOC101118057;RPLP0;DENR;LOC101116132;novel.256;RPL39;LOC101110546;LOC101114033;LOC101109380;HNRNPD;RPS8;LOC101123112;RPL27;RPS3A;LOC105607745;LOC105602999;LOC101103595;novel.1132;RPL6;RPL8;YBX1;RPL27A;RPS5;RPS26-2;LOC101110758;LOC101109545 |
| large ribosomal subunit | novel.407;MIEF1;LOC106991948;MRPL15;LOC101119765;novel.2543;NSUN4;novel.918 | RPL36AL;RPL7A;RPL7;LOC101104725;LOC105612165;RPL36;MRPL41;MRPL54;novel.2495;LOC101102690;novel.900;RPL36A;MRPL13;RPL14;MRPL36;RPL26;RPL23;LOC101123279;RPL19;LOC101121371;RPL31;RPL37;RPL35;RPL35A;RPL18;MRPL14;LOC101113344;RPL3;LOC101115154;RPL13;RPL18A;MRPL27;RPL24;LOC101110998;MRPL24;MRPL2;MRPL52;RPLP2;RPL9;RPL34;LOC105610139;RPL28;RPL102;RPL13A;LOC101123533;RPL32;MRPL21;LOC105607128;RPLP1;RPL10;RPL11;CMC1;RPL30;MRPL11;RPL38;RPL4;RPL12;UBA52;LOC101123186;RPL23A;MRPL47;LOC101109155;RPL5;RPL29;novel.2003;novel.3330;RPL26L1;LOC101111180;LOC101118057;RPLP0;RPL39;LOC101110546;LOC101114033;LOC101109380;MRPL55;LOC101104787;ICT1;LOC101123112;RPL27;MRPL32;novel.1741;MRPL57;novel.120;novel.1132;RPL6;RPL8;RPL27A;novel.3088;novel.2888;LOC101119869;LOC101109545 |
| inner mitochondrial membrane protein complex | MICU3;MTX3;LOC105616860;ATP5A1;SDHD;ATP5B | ND6;ND5;ATP5I;novel.3324;NDUFA2;NDUFA13;novel.368;LOC105606223;NDUFB7;NDUFS5;LOC101105179;COX3;COX1;ROMO1;ATP6;LOC101110916;novel.1573;TIMM10;NDUFB3;UQCRH;novel.370;ATP8;NDUFB1;ATP5J2;NDUFA3;NDUFB10;novel.371;NDUFA8;LOC101115773;novel.3901;novel.3902;ATP5J;NDUFB9;DNAJC19;NDUFA1;LOC101108778;LOC101108663;novel.3368;LOC101111410;NDUFA11;novel.2849;ND2;novel.2617;novel.1122;NDUFB11;LOC101110664;LOC101119721;novel.2048;LOC105604454;novel.1423;COX2;NDUFS7;LOC101110066;ND1;LOC101121420;SMDT1;LOC105609918;ATP5H;CHCHD10;novel.2267;NDUFS8;C5H19orf70;novel.551;ATP5L;NDUFB8;ND4;LOC101121285;novel.309;LOC101116871;MINOS1;LOC101121949;LOC101102327 |
| cytosolic large ribosomal subunit | novel.407;LOC106991948;novel.2543;novel.918 | RPL36AL;RPL7A;RPL7;LOC105612165;RPL36;LOC101102690;novel.900;RPL36A;RPL14;RPL26;RPL23;LOC101123279;RPL19;LOC101121371;RPL31;RPL37;RPL35;RPL35A;RPL18;LOC101113344;RPL3;LOC101115154;RPL13;RPL18A;RPL24;LOC101110998;RPLP2;RPL9;RPL34;LOC105610139;RPL28;RPL102;RPL13A;LOC101123533;RPL32;LOC105607128;RPLP1;RPL10;RPL11;CMC1;RPL30;RPL38;RPL4;RPL12;UBA52;LOC101123186;RPL23A;LOC101109155;RPL5;RPL29;novel.2003;novel.3330;RPL26L1;LOC101111180;LOC101118057;RPLP0;RPL39;LOC101110546;LOC101114033;LOC101109380;LOC101104787;LOC101123112;RPL27;novel.120;novel.1132;RPL6;RPL8;RPL27A;novel.3088;novel.2888;LOC101119869;LOC101109545 |
| small ribosomal subunit | DDX3X;DHX29 | novel.3935;novel.3631;MRPS26;novel.3376;RPS15;RPS10;RPS13;AURKAIP1;LOC101103316;RPS18;LOC101102096;MRPS2;CHCHD1;RPS14;RPS9;LOC443297;RPS16;RPS4X;RPS27A;RPS21;RPS19;RPS26;novel.37;novel.688;RPS23;RPS25;RPS12;MRPS34;novel.1181;MRPS14;LOC105604842;RPS24;RPS7;RPS11;MRPS23;UBA52;RPS3;FAU;novel.1438;RPS20;RPS29;MRPS18A;MRPS35;novel.574;LOC101111215;novel.283;novel.2327;RPSA;RPS27;LOC101116132;novel.256;RPS8;RPS3A;LOC105607745;RPS6;ISG15;RPS2;MRPS15;LOC101103595;MRPS28;RPS5;RPS26-2;RPS27L;LOC101110758;MRPS33 |
| respirasome | LOC101104348;LOC105616860;SDHD | ND6;ND5;novel.3324;NDUFA2;NDUFA13;novel.368;CYTB;NDUFB7;NDUFS5;LOC101105179;COX3;COX1;novel.1573;NDUFB3;UQCRH;novel.370;NDUFB1;NDUFA3;NDUFB10;novel.371;NDUFA8;LOC101115773;novel.3901;novel.3902;LOC101116886;NDUFB9;NDUFA1;LOC101118736;LOC101108778;LOC101108663;novel.3368;NDUFA11;novel.2849;ND2;novel.1122;NDUFB11;LOC101110664;LOC101121538;LOC101119721;novel.2048;LOC105604454;COX2;NDUFS7;LOC101110066;novel.369;ND1;LOC101121420;LOC105609918;NDUFS8;novel.551;SURF1;NDUFB8;ND4;LOC101121285;LOC101102327 |
| respiratory chain complex | LOC105616860;SDHD | ND6;ND5;novel.3324;NDUFA2;NDUFA13;novel.368;CYTB;NDUFB7;NDUFS5;LOC101105179;COX3;COX1;novel.1573;NDUFB3;UQCRH;novel.370;NDUFB1;NDUFA3;NDUFB10;novel.371;NDUFA8;LOC101115773;novel.3901;novel.3902;LOC101116886;NDUFB9;NDUFA1;LOC101108778;LOC101108663;novel.3368;NDUFA11;novel.2849;ND2;novel.1122;NDUFB11;LOC101110664;LOC101121538;LOC101119721;novel.2048;LOC105604454;COX2;NDUFS7;LOC101110066;novel.369;ND1;LOC101121420;LOC105609918;NDUFS8;novel.551;NDUFB8;ND4;LOC101121285;LOC101102327 |
| cytosolic small ribosomal subunit | DDX3X;DHX29 | novel.3631;novel.3376;RPS15;RPS10;RPS13;LOC101103316;RPS18;LOC101102096;RPS14;RPS9;LOC443297;RPS16;RPS4X;RPS27A;RPS21;RPS19;RPS26;novel.37;novel.688;RPS23;RPS25;RPS12;novel.1181;LOC105604842;RPS24;RPS7;RPS11;UBA52;RPS3;FAU;novel.1438;RPS20;RPS29;LOC101111215;novel.283;novel.2327;RPSA;RPS27;LOC101116132;novel.256;RPS8;RPS3A;LOC105607745;RPS6;ISG15;RPS2;LOC101103595;RPS5;RPS26-2;RPS27L;LOC101110758 |
| mitochondrial respirasome | LOC105616860;SDHD | ND6;ND5;novel.3324;NDUFA2;NDUFA13;novel.368;NDUFB7;NDUFS5;LOC101105179;COX3;COX1;novel.1573;NDUFB3;UQCRH;novel.370;NDUFB1;NDUFA3;NDUFB10;novel.371;NDUFA8;LOC101115773;novel.3901;novel.3902;LOC101116886;NDUFB9;NDUFA1;LOC101108778;LOC101108663;novel.3368;NDUFA11;novel.2849;ND2;novel.1122;NDUFB11;LOC101110664;LOC101119721;novel.2048;LOC105604454;COX2;NDUFS7;LOC101110066;ND1;LOC101121420;LOC105609918;NDUFS8;novel.551;SURF1;NDUFB8;ND4;LOC101121285;LOC101102327 |
| oxidative phosphorylation | LOC101104348;PDE12;ATP7A;LOC105616860;ATP5B | ND6;ND5;novel.3324;novel.368;LOC105606223;LOC101105179;COX3;COX1;ATP6;novel.1573;UQCRH;novel.370;ATP5J2;novel.371;NDUFA8;LOC101115773;novel.3901;novel.3902;LOC101116886;NDUFB9;LOC101118647;FAM173A;LOC101108778;LOC101108663;novel.2849;ND2;novel.1122;LOC101110664;LOC101121538;LOC101119721;novel.2048;LOC105604454;novel.1423;LOC101112013;NDUFS7;LOC101110066;ND1;LOC101121420;LOC105609918;ATP5H;CHCHD10;NDUFS8;NUPR1;NDUFB8;ND4;LOC101121285;LOC101102327 |
| oxidoreduction-driven active transmembrane transporter activity | LOC101104348;LOC101105614 | ND6;ND5;novel.3324;NDUFA13;novel.368;CYTB;LOC101105179;COX3;COX1;novel.1573;novel.370;novel.371;LOC101115773;novel.3901;novel.3902;LOC101116886;ND2;COX2;NDUFS7;novel.369;ND1;NDUFS8;SURF1;ND4;LOC101102327 |

KEGG Enrichment Pathways and Differentially Expressed Genes in Hypothalamus (IM vs. CON)

| KEGG Enrichment Pathways | Up Genes | Down Genes |
| --- | --- | --- |
| Amyotrophic lateral sclerosis | COX2;COX1;CYTB;novel.3901;novel.3324;ATP6;novel.371;novel.369;ND5;COX3;novel.370;novel.368;NXF1;ND6;ND4;ND2;ND1;LOC101103463;novel.2733;ND4L;novel.1573;ND3;BCL2;MAPK12;LOC101114597;LOC101119713 | GRIN2A;LOC101104348;R3HDM1;NEFH;ATXN2L;novel.2043;GRIA2;COX5A;TUBB6;LOC106990101;novel.589;NUP210;GRIN1;POM121C |
| Prion disease | COX2;COX1;CYTB;novel.3901;novel.3324;ATP6;novel.371;novel.369;ND5;COX3;novel.370;novel.368;ND6;ND4;NCF1;ND2;ND1;LOC101106452;novel.2733;IL1B;ND4L;PIK3R1;novel.1573;ND3;MAPK12;LOC101119713 | GRIN2A;LOC101104348;novel.2043;COX5A;TUBB6;LOC106990101;novel.589;GRIN1;PRKACA;ITPR1 |
| Coronavirus disease - COVID-19 | IRAK1;RPS6;LOC101109380;RPSA;RPS12;RPS20;RPL28;RPS11;RPL4;LOC101107098;novel.37;RPS15;LOC101113211;RPL12;novel.1438;RPL32;RPL15;LOC101113831;LOC443475;LOC101118481;RPL31;RPS24;IL1B;novel.3330;novel.283;IKBKE;PIK3R1;MYD88;MAPK12;LOC105607745;LOC101110941;LOC101103232;LOC101116132 | MASP2;PRKCG |
| Huntington disease | COX2;COX1;CYTB;novel.3901;novel.3324;ATP6;novel.371;novel.369;ND5;COX3;novel.370;novel.368;ND6;ND4;PLCB2;ND2;SOD2;ND1;LOC101103463;ND4L;novel.1573;ND3;LOC101119713 | LOC101104348;GRIA4;novel.2043;GRIA2;COX5A;TUBB6;LOC106990101;novel.589;GRIN1;ITPR1 |
| Chemical carcinogenesis - reactive oxygen species | COX2;COX1;CYTB;novel.3901;novel.3324;ATP6;novel.371;novel.369;ND5;COX3;novel.370;novel.368;ND6;ND4;NCF1;ND2;SOD2;ND1;PGFS;LOC101106452;ND4L;PIK3R1;novel.1573;ND3;SLC26A6;MAPK12 | LOC101104348;MGST3;COX5A;LOC101114663 |
| Parkinson disease | COX2;COX1;CYTB;novel.3901;novel.3324;ATP6;novel.371;novel.369;ND5;COX3;novel.370;novel.368;ND6;ND4;ND2;ND1;novel.2733;LOC101102411;ND4L;novel.1573;ND3;LOC101119713 | UCHL1;LOC101104348;novel.2043;COX5A;TUBB6;LOC106990101;PRKACA;ITPR1 |
| Retrograde endocannabinoid signaling | novel.3324;novel.371;ND5;GNG4;novel.370;ND6;ND4;PLCB2;ND2;ND1;novel.2733;ND4L;novel.1573;GNG5;ND3;MAPK12 | LOC101114066;GABRA1;LOC101104348;GRIA4;ADCY3;GABRA3;SLC32A1;GRIA2;GABRG2;ABHD6;PRKACA;PRKCG;ITPR1 |
| Diabetic cardiomyopathy | COX2;COX1;CYTB;novel.3901;novel.3324;ATP6;novel.371;novel.369;ND5;COX3;novel.370;novel.368;ND6;ND4;NCF1;PPARA;PLCB2;ND2;ND1;LOC101106452;LOC101102230;ND4L;PIK3R1;novel.1573;ND3;MAPK12 | LOC101104348;COX5A;PRKCG |
| Thermogenesis | COX2;COX1;CYTB;novel.3901;novel.3324;ATP6;novel.371;novel.369;ND5;COX3;novel.370;novel.368;RPS6;ND6;ND4;ND2;ND1;LOC101102411;ND4L;novel.1573;ND3;MAPK12 | LOC101104348;ADCY3;ARID1A;COX5A;PRKACA |
| Oxidative phosphorylation | COX2;COX1;CYTB;novel.3901;novel.3324;ATP6;novel.371;novel.369;ND5;COX3;novel.370;novel.368;ND6;ND4;ND2;ND1;ND4L;novel.1573;ND3 | ATP6V1B2;ATP6V0A1;LOC101104348;ATP6V0C;ATP6V0D1;COX5A;ATP6V1H |
| Glutamatergic synapse | GNG4;HOMER3;PLCB2;SHANK3;novel.2733;LOC101102411;GNG5 | GRIN2A;LOC101114066;SLC38A2;GRM7;GRIA4;ADCY3;GRIA2;GRM8;novel.589;TRPC1;GRM4;GRIN1;PRKACA;PRKCG;ITPR1 |
| Leishmaniasis | EEF1A1;LOC101114018;IRAK1;novel.2381;NCF1;LOC101113211;LOC101110773;LOC100125610;LOC101113831;LOC443475;LOC101106452;LOC101120236;IL1B;ITGB2;MYD88;PTPN6;LOC101102857;MAPK12;novel.423 | EEF1A2 |
| Phospholipase D signaling pathway | CYTH4;GNA12;LOC101113211;DGKQ;PLCB2;LOC105606005;PIK3CG;RAPGEF3;LOC101102411;LPAR5;PIK3R1;FCER1G;GNA13 | GRM7;RAPGEFL1;ADCY3;GRM8;DNM3;GRM4 |
| Platelet activation | LYN;ARHGAP35;PLCB2;PIK3CG;TLN1;APBB1IP;novel.2733;LOC101102411;FERMT3;PIK3R1;VAMP8;FCER1G;MAPK12;GNA13;BTK | ADCY3;PRKACA;ITPR1 |
| Legionellosis | EEF1A1;LOC101114018;novel.2381;LOC101105208;LOC101110773;LOC100125610;LOC101113831;LOC443475;NFKB2;LOC101120236;IL1B;ITGB2;MYD88;LOC101102857;novel.423 | EEF1A2 |
| Cardiac muscle contraction | COX2;COX1;CYTB;novel.3901;novel.369;COX3;novel.368;TPM4;MYL4 | ATP1A3;CACNA2D1;COX5A;ATP1A1;SLC9A1;CACNA2D2 |
| Circadian entrainment | GNG4;PLCB2;novel.2733;LOC101102411;GNG5 | GRIN2A;LOC101114066;GRIA4;ADCY3;GRIA2;novel.589;GRIN1;PRKACA;PRKCG;ITPR1 |
| GABAergic synapse | GNG4;SLC6A13;GNG5 | SLC12A5;LOC101114066;GABRA1;SLC38A2;ADCY3;GABRA3;SLC32A1;GABRG2;PRKACA;PRKCG |
| Mineral absorption | FTL;SLC40A1;SLC26A6;HEPH;novel.2792;VDR | ATP1A3;ATP2B3;HEPHL1;ATP1A1;TRPM7 |
| Nicotine addiction |  | GRIN2A;GABRA1;GRIA4;GABRA3;SLC32A1;GRIA2;GABRG2;CHRNA7;novel.589;GRIN1 |

KEGG Enrichment Pathways and Differentially Expressed Genes in Hypothalamus (IM vs.SN)

| KEGG Enrichment Pathways | Up Genes | Down Genes |
| --- | --- | --- |
| Pathways of neurodegeneration - multiple diseases | novel.3324;CYTB;ND5;COX1;COX3;ATP6;ND6;novel.368;novel.1573;novel.3901;novel.3902;LOC105606223;novel.3325;ATF4;LOC101103463;novel.370;LOC101105179;novel.371;NDUFA2;SOD1;COX2;NDUFA13;UQCRH;novel.369;RPS27A;ND2;NDUFB7;ND4;ND1;PSMC5;NDUFS5;NDUFA1;DDIT3;MAPK12;ATP8;novel.367;novel.158;LOC443297;NDUFB10;ATG2A;UBA52;LOC101108663;novel.2733;ND4L;ATP5J;NDUFB11;ATP5G2;NDUFA11;NDUFB9;LOC101116886;PLCB2;novel.2849;NDUFB3;novel.2048;BAD;NDUFB1;CALML4;AXIN1;CCS;LOC101121285;RYR1;novel.2730;LOC101110664;LOC101108778;ND3;LOC101108730;SPG11;novel.2267;GPX8;NDUFA3;LOC101119721;IL1B;FZD10;novel.2732;BCL2;NDUFA6;novel.1122;BAX;NDUFS8;LOC101121420;IL1A;BID;GPX2;novel.2303;LOC105614854;GPX7;LOC105609918;PLCB3;DNAH1;LOC101121538;CACNA1S;LOC101110066;LOC101115773;novel.2731;PSMA8;PINK1;TRAP1;HRAS;WNT6;NDUFA7;KIF5B;MAPK13;novel.2728 | ATF6;LOC101104348;ACTR1A;ATXN2L;DERL1;LOC101109608;PSMD3;MAP2K1;novel.3599;LOC101119975;LOC443104;ATP2A2;LOC101121024;LOC443240;RAB1A;CSNK2A1;MAPK1;CTNNB1;MCU;EIF2AK3;MAPK8;PSMC2;WNT5A;FZD7;PSMC4;PSMD11;LOC101103959;LOC101109981;novel.2043;EIF2S1;BRAF;TNFRSF1A;GSK3B;TARDBP;KIF5A;LOC105605934;BCL2L1;KIF5C;LOC101111156;LOC106991402;ATP5B;GRIN1;NOS1;ATG2B;TUBB6;WNT9A;LOC106990101;FZD8;SMCR8;PSMD6;SDHC;ATP5A1;LOC101122475;KLC2;PDYN;PRKCG;novel.4073;SDHD;CHMP2B;WNT3A;HSPA5;CHRNA7;GRIN2A;LOC105602665;FRAT2;APC;UCHL1;DNAH2;CSNK1A1;PRNP;LOC105601936;MAPK9;LOC101116286;ATG14;LOC101123026;SQSTM1;CASP3;GRIA3;LOC101102527;novel.511;LOC101106728;GRM1;FZD1;UBE2G2;GRIA2;novel.2030;DNAL4;DNAI2;CAMK2A;CALM2;HIP1;LOC101110904 |
| Coronavirus disease - COVID-19 | RPL7A;novel.3631;RPL36AL;RPL7;RPS15;novel.900;RPL36A;RPL14;RPS10;RPL22;RPS13;RPL31;LOC101103316;RPL26;LOC105612165;RPS18;RPS9;LOC101123279;RPL35;RPL23;LOC101121371;RPS14;RPS27A;RPL18;RPL34;RPS4X;RPS24;LOC101113344;RPL37;RPL10-2;RPL4;LOC105604842;RPL9;LOC101116132;LOC101115154;RPS25;RPL21;novel.37;RPL32;RPL19;RPSA;RPL28;LOC101102096;RPS11;novel.1438;RPS16;LOC101109155;RPS26;RPL3;RPL18A;RPS12;LOC101110998;RPL5;RPS23;RPS21;RPS7;RPS19;RPL13;RPL13A;LOC101123186;RPL24;LOC101123533;MAPK12;RPL12;RPL11;RPS20;RPL35A;RPL23A;RPLP1;RPLP2;RPL30;LOC443297;RPS27;LOC101109380;C3;UBA52;RPLP0;novel.3330;novel.1181;LOC101102690;novel.283;RPS17;RPS3;FAU;RPS6;RPL29;CMC1;LOC101104787;RPS29;LOC105607128;LOC101110941;LOC101118481;TMEM173;IKBKE;LOC101109545;RPL38;RPL39;LOC100037664;LOC105607745;LOC101123672;RPS8;LOC101114033;CFD;RPL27;RPL36;RPS5;RPS2;RPL27A;IRAK1;novel.1132;RPL10;RPS3A;LOC101103232;RPL8;LOC101110546;LOC101110758;IL1B;novel.256;RPL15;novel.3427;novel.2793;LOC101110176;LOC101123419;RPS27L;TYK2;LOC105605879;LOC101107216;LOC101117120;LOC101123112;C5;C6;LOC101103595;C7;LOC101107098;C1QB;LOC105610137;LOC101122834;C1S;LOC101105336;LOC101123159;RPL22L1;C1QA;ISG15;RPL3L;LOC101107430;LOC101111180;LOC101117013;novel.2177;MAP3K7CL;ACE2;RPS26-2;LOC101104286;LOC106991949;LOC106990881;MAPK13;novel.1284;LOC101106855;CFB | MAPK1;JAK1;MAPK8;TNFRSF1A;STAT3;novel.2577;PIK3CA;MASP2;PRKCG;IFNAR1;MAPK9;EGFR;SELP |
| Alzheimer disease | novel.3324;CYTB;ND5;COX1;COX3;ATP6;ND6;novel.368;novel.1573;novel.3901;novel.3902;LOC105606223;novel.3325;ATF4;LOC101103463;novel.370;LOC101105179;novel.371;NDUFA2;COX2;NDUFA13;UQCRH;novel.369;ND2;NDUFB7;ND4;ND1;PSMC5;NDUFS5;NDUFA1;DDIT3;ATP8;novel.367;NDUFB10;ATG2A;LOC101108663;novel.2733;ND4L;ATP5J;NDUFB11;ATP5G2;NDUFA11;NDUFB9;LOC101116886;PLCB2;novel.2849;NDUFB3;novel.2048;BAD;NDUFB1;CALML4;AXIN1;LOC101121285;novel.2730;LOC101110664;LOC101108778;ND3;LOC101108730;novel.2267;NDUFA3;LOC101119721;IL1B;FZD10;novel.2732;NDUFA6;novel.1122;NDUFS8;LOC101121420;IL1A;BID;novel.2303;LOC105614854;LOC105609918;APOE;PLCB3;LOC101121538;CACNA1S;LOC101110066;LOC101115773;novel.2731;PSMA8;HRAS;WNT6;NDUFA7;KIF5B;novel.2728 | ATF6;LOC101115614;LOC101104348;LOC101109608;PSMD3;MAP2K1;novel.3599;SLC39A13;LOC101119975;LOC443104;ATP2A2;LOC101121024;LOC443240;SLC39A9;CSNK2A1;MAPK1;SLC39A3;CTNNB1;MCU;EIF2AK3;MAPK8;PSMC2;IRS2;WNT5A;FZD7;RTN4;PSMC4;PSMD11;LOC101103959;RTN3;LOC101109981;novel.2043;EIF2S1;BRAF;TNFRSF1A;GSK3B;KIF5A;LOC105605934;LOC106991605;KIF5C;LOC101111156;LOC106991402;ATP5B;GRIN1;NOS1;ATG2B;TUBB6;WNT9A;LOC101116975;LOC106990101;PIK3CA;FZD8;PSMD6;SDHC;ATP5A1;LOC101122475;AKT3;KLC2;novel.4073;SDHD;WNT3A;CHRNA7;GRIN2A;LOC105602665;FRAT2;APC;BACE1;CSNK1A1;NCSTN;LOC105601936;MAPK9;LOC101116286;ATG14;SLC39A10;CASP3;LOC101102527;novel.511;FZD1;CALM2;LOC101110904 |
| Amyotrophic lateral sclerosis | novel.3324;CYTB;ND5;COX1;COX3;ATP6;ND6;novel.368;novel.1573;novel.3901;novel.3902;LOC105606223;novel.3325;ATF4;LOC101103463;novel.370;LOC101105179;novel.371;NDUFA2;SOD1;COX2;NDUFA13;UQCRH;novel.369;ND2;NDUFB7;ND4;ND1;NXF1;PSMC5;NDUFS5;NDUFA1;DDIT3;MAPK12;ATP8;novel.367;novel.158;NDUFB10;ATG2A;LOC101108663;novel.2733;ND4L;ATP5J;NDUFB11;ATP5G2;PFN1;NDUFA11;NDUFB9;LOC101116886;novel.2849;NDUFB3;novel.2048;BAD;CHCHD10;NDUFB1;CCS;LOC101121285;novel.2730;LOC101110664;LOC101108778;ND3;LOC101108730;SPG11;novel.2267;GPX8;NDUFA3;LOC101119721;novel.2732;BCL2;NDUFA6;novel.1122;BAX;NDUFS8;LOC101121420;HDAC6;BID;GPX2;novel.2303;GPX7;LOC105609918;DNAH1;LOC101121538;LOC101110066;LOC101115773;novel.2731;PSMA8;LOC101109899;PINK1;NRG4;LOC101117013;NDUFA7;novel.2548;KIF5B;MAPK13;novel.2728 | ATF6;POM121C;LOC101104348;ACTR1A;ATXN2L;DERL1;UBQLN1;LOC101109608;PSMD3;LOC101119975;LOC443104;RAB1A;MCU;EIF2AK3;NUP62;PSMC2;PSMC4;PSMD11;LOC101103959;novel.2043;EIF2S1;NUP153;RAE1;TNFRSF1A;TARDBP;KIF5A;BCL2L1;KIF5C;ERBB4;LOC101111156;ATP5B;GRIN1;NOS1;ATG2B;TUBB6;LOC106990101;SMCR8;PSMD6;SDHC;ATP5A1;LOC101122475;SETX;KLC2;ACTB2;SDHD;CHMP2B;HSPA5;RANBP2;GRIN2A;NDC1;DNAH2;NUP133;PFN2;R3HDM1;LOC105601936;LOC101116286;ATG14;SQSTM1;CASP3;LOC101102527;GRIA2;novel.2030;DNAL4;DNAI2;LOC101110904 |
| Ribosome | RPL7A;novel.3631;RPL36AL;RPL7;LOC101104725;RPS15;novel.900;RPL36A;RPL14;RPS10;RPL22;RPS13;novel.3935;RPL31;LOC101103316;RPL26;LOC105612165;RPS18;RPS9;LOC101123279;RPL35;MRPL13;RPL23;LOC101121371;MRPS2;RPS14;RPS27A;RPL18;RPL34;RPS4X;RPS24;LOC101113344;RPL37;RPL10-2;RPL4;LOC105604842;RPL9;LOC101116132;LOC101115154;RPS25;RPL21;novel.37;RPL32;RPL19;RPSA;RPL28;LOC101102096;RPS11;novel.1438;RPS16;LOC101109155;RPS26;RPL3;RPL18A;RPS12;LOC101110998;RPL5;RPS23;RPS21;RPS7;RPS19;RPL13;RPL13A;MRPL27;LOC101123186;RPL24;LOC101123533;RPL12;RPL11;RPS20;RPL35A;RPL23A;RPLP1;MRPL14;RPLP2;RPL30;LOC443297;RPS27;LOC101109380;UBA52;RPLP0;novel.3330;novel.1181;LOC101102690;novel.283;RPS17;RPS3;FAU;MRPL2;RPS6;RPL29;CMC1;LOC101104787;RPS29;MRPS14;LOC105607128;LOC101110941;LOC101118481;LOC101109545;RPL38;RPL39;MRPL24;LOC100037664;LOC105607745;MRPL36;RPS8;novel.574;LOC101114033;RPL27;RPL36;RPS5;MRPL21;RPS2;RPL27A;novel.1132;RPL10;RPS3A;MRPL32;MRPL10;LOC101103232;RPL8;LOC101110546;LOC101110758;novel.256;RPL15;novel.2793;novel.1741;LOC101110176;MRPS18A;RPS27L;LOC101107216;LOC101117120;LOC101123112;MRPL11;LOC101103595;LOC101107098;LOC105610137;LOC101122834;LOC101105336;RPL22L1;RPL3L;MRPL28;LOC101111180;novel.2177;RPS26-2;LOC101104286;LOC106991949;LOC106990881;novel.1284;MRPL34;LOC101106855;MRPS16 | novel.2577;MRPL15 |
| Prion disease | novel.3324;CYTB;ND5;COX1;COX3;ATP6;ND6;novel.368;novel.1573;novel.3901;novel.3902;LOC105606223;novel.3325;ATF4;novel.370;LOC101105179;novel.371;NDUFA2;SOD1;COX2;NDUFA13;UQCRH;novel.369;ND2;NDUFB7;ND4;ND1;PSMC5;NDUFS5;NDUFA1;DDIT3;MAPK12;ATP8;novel.367;novel.158;NDUFB10;LOC101106452;LOC101108663;novel.2733;ND4L;ATP5J;NDUFB11;ATP5G2;NDUFA11;NDUFB9;LOC101116886;novel.2849;NDUFB3;novel.2048;NCF1;BAD;NDUFB1;LOC101121285;RYR1;novel.2730;LOC101110664;LOC101108778;ND3;LOC101108730;novel.2267;NDUFA3;LOC101119721;IL1B;novel.3427;novel.2732;NDUFA6;NCF4;novel.1122;BAX;NDUFS8;LOC101121420;IL1A;C5;novel.2303;C6;C7;C1QB;LOC105609918;LOC101121538;CACNA1S;LOC101110066;LOC101115773;C1QA;novel.2731;PSMA8;NDUFA7;KIF5B;MAPK13;novel.2728 | PRKACA;NCAM1;HSPA8;LOC101104348;LOC101109608;PSMD3;LOC101119975;LOC443104;STIP1;PRKACB;CSNK2A1;MAPK1;MCU;EIF2AK3;MAPK8;PSMC2;PSMC4;PSMD11;LOC101103959;LOC101109981;novel.2043;EIF2S1;CAV1;GSK3B;KIF5A;KIF5C;ATP5B;GRIN1;CREB3L2;TUBB6;LOC106990101;PIK3CA;PSMD6;SDHC;ATP5A1;LOC101122475;KLC2;SDHD;HSPA5;GRIN2A;PRNP;LOC105601936;MAPK9;LOC101116286;LOC101123026;CASP3;LOC101102527;novel.511 |
| Parkinson disease | novel.3324;CYTB;ND5;COX1;COX3;ATP6;ND6;novel.368;novel.1573;novel.3901;novel.3902;LOC105606223;novel.3325;ATF4;novel.370;LOC101105179;novel.371;NDUFA2;SOD1;COX2;NDUFA13;UQCRH;novel.369;RPS27A;ND2;NDUFB7;ND4;ND1;PSMC5;NDUFS5;NDUFA1;DDIT3;ATP8;novel.367;novel.158;LOC443297;NDUFB10;UBA52;LOC101108663;novel.2733;ND4L;ATP5J;NDUFB11;ATP5G2;NDUFA11;NDUFB9;LOC101116886;novel.2849;NDUFB3;novel.2048;NDUFB1;CALML4;LOC101121285;novel.2730;LOC101110664;LOC101108778;ND3;LOC101108730;novel.2267;NDUFA3;LOC101119721;novel.2732;NDUFA6;novel.1122;BAX;NDUFS8;LOC101121420;novel.2303;LOC105614854;LOC105609918;LOC101121538;LOC101110066;LOC101115773;novel.2731;PSMA8;PINK1;TRAP1;NDUFA7;KIF5B;novel.2728 | ATF6;PRKACA;LOC101115614;LOC101104348;LOC101109608;PSMD3;SLC39A13;LOC101119975;LOC443104;LOC101121024;SLC39A9;PRKACB;SLC39A3;MCU;EIF2AK3;ADCY5;MAPK8;PSMC2;PSMC4;PSMD11;LOC101103959;LOC101109981;novel.2043;EIF2S1;KIF5A;BCL2L1;KIF5C;LOC106991402;ATP5B;TUBB6;LOC101116975;LOC106990101;PSMD6;SDHC;ATP5A1;LOC101122475;TXNL1;KLC2;SDHD;LOC105606029;HSPA5;GNAL;UCHL1;LOC105601936;DRD1;MAPK9;LOC101116286;SLC39A10;CASP3;LOC101102527;novel.511;UBE2G2;novel.2030;CAMK2A;GNAI1;CALM2;novel.197 |
| Huntington disease | LOC105604154;novel.3324;CYTB;ND5;COX1;COX3;ATP6;ND6;novel.368;novel.1573;novel.3901;novel.3902;LOC105606223;novel.3325;LOC101103463;novel.370;LOC101105179;novel.371;NDUFA2;SOD1;COX2;NDUFA13;UQCRH;novel.369;ND2;NDUFB7;POLR2I;ND4;ND1;PSMC5;NDUFS5;NDUFA1;ATP8;novel.367;novel.158;NDUFB10;ATG2A;POLR2J;LOC101108663;ND4L;ATP5J;NDUFB11;ATP5G2;NDUFA11;NDUFB9;LOC101116886;PLCB2;novel.2849;NDUFB3;novel.2048;NDUFB1;LOC101121285;LOC101110664;LOC101108778;ND3;LOC101108730;novel.2267;GPX8;NDUFA3;LOC101119721;NDUFA6;novel.1122;BBC3;BAX;NDUFS8;LOC101121420;GPX2;novel.2303;LOC105616906;GPX7;LOC105609918;PLCB3;DNAH1;LOC101121538;LOC101110066;LOC101115773;PSMA8;AP2S1;NDUFA7;KIF5B | LOC101104348;ACTR1A;LOC101109608;PSMD3;LOC101119975;LOC443104;POLR2A;KCNJ10;MAPK8;PSMC2;PSMC4;PSMD11;LOC101103959;LOC101109981;AP2B1;novel.2043;AP2A1;KIF5A;KIF5C;RCOR1;LOC101111156;AP2M1;CREBBP;ATP5B;GRIN1;CREB3L2;ATG2B;TUBB6;CLTC;LOC106990101;PSMD6;SDHC;ATP5A1;LOC101122475;KLC2;SDHD;DNAH2;LOC105601936;TFAM;MAPK9;LOC101116286;ATG14;LOC101123026;CASP3;GRIA3;LOC101102527;novel.511;GRIA2;DNAL4;DNAI2;TAF4B;HIP1;LOC101110904 |
| Chemical carcinogenesis - reactive oxygen species | novel.3324;CYTB;ND5;COX1;COX3;ATP6;ND6;novel.368;novel.1573;novel.3901;novel.3902;LOC105606223;novel.3325;novel.370;LOC101105179;novel.371;NDUFA2;SOD1;COX2;NDUFA13;UQCRH;novel.369;ND2;NDUFB7;ND4;ND1;NDUFS5;NDUFA1;SLC26A6;MAPK12;ATP8;novel.367;novel.158;NDUFB10;LOC101106452;LOC101108663;ND4L;ATP5J;NDUFB11;ATP5G2;NDUFA11;NDUFB9;LOC101116886;novel.2849;PLD2;NDUFB3;novel.2048;GSTT2B;NCF1;BAD;NDUFB1;LOC101121285;LOC101110664;LOC101108778;ND3;novel.2267;NDUFA3;LOC101119721;EPHX2;NDUFA6;novel.1122;NDUFS8;LOC101121420;LOC105609918;PGFS;LOC101121538;AS3MT;LOC101110066;LOC101115773;OSTF1;PRKD2;HRAS;PTPN11;NDUFA7;MAPK13 | LOC101104348;MAP2K1;novel.3079;MAPK1;ARNT;MAPK8;LOC101114408;LOC101109981;VEGFA;BRAF;HIF1A;LOC106991605;ATP5B;AHR;PIK3CA;SDHC;ATP5A1;AKT3;FOXO3;EPHX3;PDPK1;SDHD;PTPRJ;MAP2K4;MAPK9;EGFR;LOC101102527;novel.511;ABL1;SOS1 |
| Thermogenesis | novel.3324;CYTB;ATP5I;ND5;COX1;COX3;ATP6;ND6;novel.368;novel.1573;novel.3901;novel.3902;LOC105606223;novel.3325;novel.370;LOC101105179;LOC101107153;novel.371;NDUFA2;LOC106991149;COX2;NDUFA13;UQCRH;novel.369;ND2;NDUFB7;ND4;ND1;NDUFS5;C11H17orf89;ATP5J2;NDUFA1;MAPK12;ATP8;RPS6KB2;novel.367;NDUFB10;TSC2;LOC101108663;ND4L;ATP5J;NDUFAF3;RPS6;NDUFB11;ATP5G2;NDUFA11;NDUFB9;LOC101116886;novel.2849;NDUFB3;novel.2048;NDUFB1;LOC101109345;ATP5L;RPS6KA1;LOC101121285;LOC101110664;LOC101108778;LOC101112076;ND3;novel.2267;NDUFA3;LOC101119721;SLC25A29;NDUFA6;novel.1122;NDUFS8;LOC101121420;ADCY4;LOC105609918;LOC101121538;LOC101110066;LOC101115773;OSTF1;HRAS;NDUFA7;MAPK13 | PRKACA;LOC101104348;ADCY9;PRKACB;ADCY3;TSC1;ADCY5;LOC101103154;SLC25A20;ATP5B;LOC101105425;CREB3L2;ARID1A;SDHC;ATP5A1;RPS6KA3;ACTB2;SDHD;LOC105606029;CNR1;ADCY1;SMARCD1;LOC101102527;SOS1;novel.197 |
| Diabetic cardiomyopathy | novel.3324;CYTB;ND5;COX1;COX3;ATP6;ND6;novel.368;novel.1573;novel.3901;novel.3902;LOC105606223;novel.3325;novel.370;LOC101105179;novel.371;NDUFA2;COX2;NDUFA13;UQCRH;novel.369;ND2;NDUFB7;ND4;ND1;NDUFS5;NDUFA1;MAPK12;ATP8;novel.367;NDUFB10;LOC101106452;LOC101108663;ND4L;ATP5J;NDUFB11;ATP5G2;NDUFA11;NDUFB9;LOC101116886;PLCB2;novel.2849;NDUFB3;novel.2048;NCF1;TGFB1;NDUFB1;LOC101121285;LOC101110664;LOC101108778;ND3;LOC101117953;novel.2267;NDUFA3;LOC101119721;NDUFA6;NCF4;novel.1122;NDUFS8;LOC101121420;MPC1;LOC105609918;PLCB3;LOC101121538;LOC101110066;LOC101115773;PPARA;NDUFA7;MAPK13 | LOC101104348;PDHA1;GFPT1;ATP2A2;MAPK8;novel.2897;LOC101109981;PPP1CC;GSK3B;LOC101117153;LOC106991605;ATP5B;TGFBR2;PARP1;PIK3CA;SDHC;ATP5A1;AKT3;PRKCG;SDHD;TBC1D4;MAPK9;LOC101102527;novel.511;CAMK2A |
| Retrograde endocannabinoid signaling | novel.3324;ND5;ND6;novel.1573;novel.3902;novel.370;novel.371;NDUFA2;NDUFA13;ND2;NDUFB7;ND4;ND1;NDUFS5;NDUFA1;MAPK12;NDUFB10;novel.2733;ND4L;NDUFB11;GNG11;NDUFA11;NDUFB9;GNG5;PLCB2;NDUFB3;NDUFB1;novel.2730;ND3;NDUFA3;LOC101103602;GNGT1;novel.2732;NDUFA6;NDUFS8;ADCY4;LOC101103356;GNB3;PLCB3;LOC101116593;CACNA1S;LOC101115773;novel.314;novel.2731;LOC101112183;GABRE;NDUFA7;MAPK13;novel.2728 | KCNJ9;PRKACA;LOC101104348;ADCY9;PRKACB;ADCY3;MAPK1;ADCY5;MAPK8;DAGLA;GABRG1;GABRR2;PRKCG;GABRA4;LOC105606029;GABRR1;CNR1;ABHD6;ADCY1;MAPK9;GABRA1;LOC101123026;GRIA3;SLC32A1;GRM1;RIMS1;GRIA2;GNAI1;GNAO1;novel.197 |
| Oxidative phosphorylation | novel.3324;CYTB;ATP5I;ND5;COX1;COX3;ATP6;ND6;novel.368;novel.1573;novel.3901;novel.3902;LOC105606223;novel.3325;novel.370;LOC101105179;LOC101107153;novel.371;NDUFA2;COX2;NDUFA13;UQCRH;novel.369;ND2;NDUFB7;ND4;ND1;NDUFS5;ATP5J2;NDUFA1;ATP8;novel.367;NDUFB10;LOC101108663;ND4L;ATP5J;NDUFB11;ATP5G2;NDUFA11;NDUFB9;LOC101116886;novel.2849;NDUFB3;novel.2048;NDUFB1;ATP5L;LOC101121285;LOC101110664;LOC101108778;ND3;novel.2267;NDUFA3;LOC101119721;NDUFA6;novel.1122;ATP6V0E1;NDUFS8;LOC101121420;TCIRG1;LOC105609918;LOC101121538;LOC101110066;LOC101115773;NDUFA7 | LOC101104348;ATP6V1D;ATP6AP1;ATP6V0A1;ATP6V0D1;ATP6V1H;ATP5B;LOC101105425;SDHC;ATP5A1;ATP6V1B2;SDHD;ATP6V1A;ATP6V0C;LOC101102527 |
| Axon guidance | LIMK2;SEMA3B;MRCL3;FES;RRAS;RHOD;BOC;PLXNB2;HRAS;PTPN11;MYL9;SSH3;EFNA4;PARD3B;PARD6G | SEMA7A;UNC5C;CDC42;EFNB2;novel.3079;SEMA5A;EPHB2;SEMA4C;EFNB3;MAPK1;DPYSL5;PARD6B;PAK7;PLXNA1;WNT5A;TRPC5;BMPR2;LRIG2;SEMA4G;PTCH1;LOC106991788;BMPR1B;DCC;SRGAP2;GSK3B;LOC105605934;RGMA;SRGAP3;CFL1;NTN1;UNC5D;PIK3CA;EPHB1;PDPK1;novel.4073;NTN3;UNC5B;LOC106992038;LOC105602665;SEMA6B;LOC101108984;LRRC4;EFNB1;ABLIM2;DPYSL3;SSH1;SSH2;EPHA5;BMP7;ABL1;SEMA6A;TRPC4;CAMK2A;SEMA3G;SEMA5B;PAK6;EPHA8;GNAI1;EPHA4 |
| Non-alcoholic fatty liver disease | CYTB;COX1;COX3;novel.368;novel.3901;novel.3325;ATF4;LOC101103463;LOC101105179;NDUFA2;COX2;NDUFA13;UQCRH;novel.369;NDUFB7;NDUFS5;NDUFA1;DDIT3;MAPK12;novel.367;NDUFB10;LOC101108663;NDUFB11;NDUFA11;NDUFB9;LOC101116886;novel.2849;NDUFB3;novel.2048;TGFB1;NDUFB1;LOC101121285;LOC101110664;LOC101108778;NDUFA3;LOC101119721;IL1B;NR1H3;NDUFA6;novel.1122;BAX;NDUFS8;LOC101121420;IL1A;BID;LOC105609918;LOC101121538;LOC101110066;LOC101115773;PPARA;NDUFA7;MAPK13 | CDC42;GSK3A;ADIPOR2;EIF2AK3;MAPK8;IRS2;MLXIP;EIF2S1;TNFRSF1A;GSK3B;LOC106991605;PIK3CA;SDHC;AKT3;SDHD;BCL2L11;ADIPOR1;MAPK9;CASP3;LOC101102527 |
| Cholinergic synapse | ATF4;novel.2733;GNG11;GNG5;PLCB2;PIK3R6;novel.2730;GNGT1;novel.2732;BCL2;ADCY4;GNB3;PLCB3;LOC101116593;CACNA1S;novel.2889;novel.314;novel.2731;LOC101112183;HRAS;novel.2728 | PRKACA;MAP2K1;GNA11;ADCY9;PRKACB;ADCY3;MAPK1;ADCY5;LOC106991605;CHRNB2;CREB3L2;KCNQ2;PIK3CA;CHRNA4;AKT3;KCNJ4;PRKCG;LOC105606029;CHRNA7;ADCY1;CHRNA3;CHRM2;LOC101123026;CAMK2A;GNAI1;GNAO1;novel.197 |
| Progesterone-mediated oocyte maturation | ANAPC16;ANAPC2;ANAPC15;MAPK12;ANAPC11;novel.742;ANAPC4;RPS6KA1;CDC25B;MAD2L2;ADCY4;PLK1;KIF22;CNTD2;ANAPC10;MAPK13;LOC101108105 | PRKACA;MAP2K1;ADCY9;PRKACB;ADCY3;MAPK1;ADCY5;MAPK8;CPEB3;FZR1;CCNJ;BRAF;IGF1R;LOC106991605;LOC105611974;PIK3CA;RPS6KA3;AKT3;LOC105606029;PKMYT1;CPEB4;ADCY1;MAPK9;CPEB2;HSP90AB1;GNAI1;novel.197 |
| Cardiac muscle contraction | CYTB;COX1;COX3;novel.368;novel.3901;novel.3325;LOC101105179;COX2;UQCRH;novel.369;novel.367;LOC101108663;LOC101116886;novel.2849;novel.2048;LOC101121285;TPM4;LOC101110664;LOC101108778;LOC101119721;novel.1122;LOC101121420;TPM2;LOC105609918;LOC101121538;CACNA1S;LOC101110066;TPM1 | CACNG7;SLC9A1;ATP2A2;SLC8A2;CACNA2D1;ATP1A1;CACNA2D2;ATP1A3;CACNB4;ATP1B2;ASPHD2;CACNB1;LOC101102527;CACNG8;SLC9A6 |
| GABAergic synapse | GNG11;GNG5;SLC6A13;GNGT1;ADCY4;GNB3;LOC101116593;CACNA1S;novel.314;LOC101112183;SLC6A12;GABRE | SLC6A1;PRKACA;GLS;ADCY9;PRKACB;ADCY3;ADCY5;SLC12A5;SLC38A1;GABRG1;SLC38A2;GABRR2;PRKCG;GABRA4;LOC105606029;GABARAPL1;LOC106990907;GABRR1;GAD2;ADCY1;GPHN;GABRA1;LOC101123026;SLC32A1;GNAI1;GNAO1;novel.197 |

KEGG Enrichment Pathways and Differentially Expressed Genes in Hypothalamus (SN vs. CON)

| KEGG Enrichment Pathways | Up Genes | Down Genes |
| --- | --- | --- |
| Metabolic pathways | GALNT2;GLA;XYLT1;SETD1B;SRD5A1;LPCAT1;AASDHPPT;GFPT1;PDHA1;NUDT9;PGM2L1;MAN2A2;ADCY5;H6PD;VKORC1L1;AGL;PFKFB3;MUT;ADCY9;B4GALT5;GLUD1;COLGALT2;EARS2;GALNT16;GLS;LOC101104348;MAN1A1;ST3GAL1;LBR;MGAT5;LOC101117153;DHCR24;ST6GAL1;PPCS;LOC106990907;ACP2;ADAL;PTGES3;BCAT1;PGAM1;FKTN;CBR4;LPIN1;ZNF618;AHCYL2;ALDH4A1;novel.3870;ADO;LARGE;LOC106991619;ATP6V1D;AGPAT3;SUCLA2;MTMR4;PRDM2;LOC101109313;LOC101105425;EPT1;ST6GAL2;LOC101121783;CNDP2;IDS;PNPO;SETDB2;INPP5A;PI4KB;ISPD;B4GALT1;PIK3CA;THTPA;HACD3;DCAKD;MGAT4A;IVD;HACD2;LPIN2;IDUA;PIK3C2A;ZNF609;LOC105615198;NOS1;NDST1;GALNT10;novel.915;novel.1275;PDE11A;LCLAT1;ASNS;novel.197;FUT9;ADCY3;PDXK;LOC101114408;ALDH18A1;PAFAH1B1;MOGAT1;ALDH3A2;LOC105606029;ECHDC1;ACLY;AK9;LOC101108953;FADS2;STS;PGAP1;LOC101117195;GLDC;LANCL1;ATP6AP1;ENPP4;PPAT;GAD2;PDE8B;PIK3CG;ACSS2;SETMAR;LOC101117505;LOC105606098;CERK;PISD;ITPKB;ELOVL6;DNMT3A;RPP14;UGT8;NDST4;PGM1;PI4K2A;INPP4A;ATP5A1;LOC105605822;SPTLC3;SPTLC2;ATP6V0A1;RPE65;GLCE;ASH1L;LOC101111006;NMNAT2;SDHD;MCCC2;ST8SIA5;DCK;PANK1;SCD;MINPP1;SYNJ2;GANC;GUCY1A2;PAICS;B3GNT4;PDE3A;BDH1;GMPS;novel.1825;SYNJ1;LACC1;SGSH;HK2;GALNT7;LOC105615111;GPT2;TMEM186;novel.269;NAT8L;novel.2137;DGKD;ETNK1;DEGS1;SRR;PLPP3;PFKL;ENTPD4;PDE5A;novel.3990;PRPS1;novel.2503;LPCAT2;ATP4A;ST3GAL2;ALDH6A1;novel.3463;ATP5B;novel.4061;novel.3763 | ND6;ND5;ATP5I;novel.3324;NDUFA2;ACSM4;NDUFA13;novel.368;LOC105606223;CYTB;novel.3284;NDUFB7;LOC101107153;NDUFS5;LOC101105179;COX3;CKB2;COX1;ATP6;INPP5K;DGAT1;OPLAH;ACY1;novel.1573;NTPCR;NDUFB3;UQCRH;novel.3070;MMAB;UCKL1;SPHK1;SIRT3;novel.370;ATP8;NDUFB1;ACAA1;ATP5J2;CAD;COQ2;NDUFA3;NDUFB10;novel.371;NDUFA8;LOC101113505;LOC101115773;DGKQ;novel.3901;novel.3902;KHK;FAM213B;CHKB;LOC101116886;ATP5J;NDUFB9;PCBD2;IMPDH2;AMT;HSD17B8;NDUFA1;NAGK;MOCS2;DUT;LDHD;FDPS;GUK1;LOC101108778;LOC101108663;novel.3368;APRT;ASPA;MIF;NDUFA11;novel.2849;LOC101114663;ND2;LOC105611673;UROD;novel.1122;CKM;LOC105608370;NDUFB11;LOC101110664;LOC101121538;HAGHL;PGLS;NANS;novel.2566;HDDC2;LTC4S;ENO3;TMEM86B;AADAT;ACSF3;LOC101119721;IDNK;NUDT2;GDPD3;novel.2048;DPM3;novel.1423;COX2;HEXDC;TECR;SMPD2;RENBP;NDUFS7;LOC101110066;novel.369;LOC101104943;LOC101102072;ND1;LOC105616883;COQ7;GSTT2B;LOC101121420;MVD;PROCA1;LOC101118831;GGT5;NME4;LOC101107260;MTHFR;TST;HYI;LOC105612707;HOGA1;CA4;ADSSL1;LOC105609918;ATP5H;novel.2267;NDUFS8;LSS;GSTP1;ACYP2;PCBD1;HSD17B6;novel.551;QPRT;GALK1;GNMT;ATP5L;SMPD4;LOC101106395;ST6GALNAC2;TSTA3;CMPK1;LOC106990244;LOC101119050;CYP3A24;GALE;CMBL;PLD2;AMDHD2;NT5M;SAT2;CERCAM;novel.3883;HDDC3;ST6GALNAC4;TPK1;ALG3;NDUFB8;ND4;GPX4;LOC101121285;HACD4;PIGQ;SAT1;GCSH;novel.309;NADSYN1;MAT1A;NUDT16;LOC101120465;ENOSF1;AGPAT2;BLVRB;PLA2G7;ASL;LOC101102327 |
| Pathways of neurodegeneration - multiple diseases | LOC443240;LOC443104;MAPK1;MAP2K1;LOC101121024;ATF6;CSNK2A1;WNT5A;BCL2L1;ACTR1A;RB1CC1;novel.3599;LOC101104348;MAPK8;PSMD3;UBE2G2;RAB8A;LOC101109981;CTNNB1;novel.511;ATXN2L;DERL1;CSNK2A2;novel.3624;RAB1A;LOC101119975;KIF5A;TNFRSF1A;FZD7;GSK3B;LOC101109608;ATP2A2;UBE2L3;DNAH2;LOC101103959;NOS1;LOC105605934;TARDBP;EIF2AK3;GRM5;CHMP2B;novel.2701;LOC106991402;DNAH6;SQSTM1;EIF2S1;DNAL4;CSNK1A1;LOC101106728;ATP5A1;SDHD;GRIN1;BECN1;DNAI2;DNAH3;TOMM40L;WNT9A;PSMD11;LRP6;novel.2043;WNT3A;LOC101123026;FZD8;NRAS;novel.1334;novel.2030;ATP5B | ND6;ND5;LOC101103463;SOD1;novel.3324;NDUFA2;NDUFA13;novel.368;LOC105606223;CYTB;NDUFB7;NDUFS5;ATF4;LOC101105179;COX3;novel.158;COX1;ATP6;novel.1573;NDUFB3;UQCRH;LOC443297;novel.370;ATP8;NDUFB1;NDUFA3;NDUFB10;novel.371;NDUFA8;PSMC5;LOC101115773;novel.3901;novel.3902;RPS27A;LOC101116886;ATP5J;ATG2A;PSMD4;NDUFB9;CCS;NDUFA1;LOC101108778;LOC101108663;novel.3368;NDUFA11;novel.2849;MAPK12;ND2;HRAS;novel.1122;NDUFB11;LOC101110664;LOC101121538;UBA52;LOC101108730;BAX;LOC101119721;novel.2303;PSMC3;novel.2048;novel.1423;COX2;NDUFS7;LOC101110066;novel.369;ND1;LOC101121420;LOC105609918;ATP5H;novel.2267;NDUFS8;RYR1;HTRA2;novel.551;novel.3517;LOC105614854;PSMA8;NDUFB8;ND4;LOC101121285;novel.2733;novel.309;FZD10;DKK2;LOC101102327 |
| Coronavirus disease - COVID-19 | MAPK1;JAK1;MAPK8;STAT3;MYD88;TNFRSF1A;PIK3CA;LOC443475;LOC105605927;PIK3R1;EGFR;MAVS;LOC101113831;IFNAR1;TRAF3;LOC101111946 | RPL36AL;RPL7A;novel.3631;RPL7;LOC105612165;RPL36;RPS15;LOC101102690;RPS10;novel.900;RPS13;RPL36A;RPL14;LOC101103316;RPS18;LOC101102096;RPL22;RPL26;RPL23;LOC101123279;RPL19;RPS14;RPS9;LOC101121371;RPL31;LOC443297;RPS16;RPL37;RPS4X;RPL35;RPL35A;RPL18;RPL21;RPS27A;LOC101113344;RPL3;RPS21;LOC101115154;C3;RPL13;RPL18A;RPS19;RPL24;LOC101110998;RPS26;novel.37;novel.688;RPS23;RPLP2;RPL9;RPL34;RPS25;LOC105610139;RPL28;RPS12;RPL102;novel.1181;RPL13A;LOC105604842;LOC101123533;RPS24;RPL32;LOC105607128;RPS7;RPLP1;RPS11;RPL10;RPL11;CMC1;RPL30;MAPK12;RPL38;RPL4;RPS17;RPL12;UBA52;LOC101123186;RPS3;FAU;novel.1438;RPS20;RPL23A;RPS29;ACE2;LOC101109155;RPL5;RPL29;novel.2003;novel.3330;novel.283;novel.2327;RPL26L1;RPSA;LOC101111180;RPS27;LOC101118057;RPLP0;LOC101116132;novel.256;RPL39;LOC101110546;LOC101114033;LOC101109380;LOC101104787;RPS8;LOC101123112;RPL27;LOC101123672;RPS3A;LOC105607745;IKBKE;RPS6;TMEM173;ISG15;RPS2;LOC101123419;CFD;LOC101103595;novel.1132;RPL6;RPL8;RPL27A;RPS5;RPS26-2;RPS27L;LOC101110758;LOC101119869;LOC101109545 |
| Ribosome | MRPL15;LOC101119765 | RPL36AL;novel.3935;RPL7A;novel.3631;RPL7;LOC101104725;LOC105612165;RPL36;RPS15;LOC101102690;RPS10;novel.900;RPS13;RPL36A;MRPL13;RPL14;LOC101103316;RPS18;MRPL36;LOC101102096;RPL22;RPL26;MRPS2;RPL23;LOC101123279;RPL19;RPS14;RPS9;LOC101121371;RPL31;LOC443297;RPS16;RPL37;RPS4X;RPL35;RPL35A;RPL18;MRPL14;RPL21;RPS27A;LOC101113344;RPL3;RPS21;LOC101115154;RPL13;RPL18A;RPS19;MRPL27;RPL24;LOC101110998;MRPL24;RPS26;novel.37;MRPL2;novel.688;RPS23;RPLP2;RPL9;RPL34;RPS25;LOC105610139;RPL28;RPS12;RPL102;novel.1181;MRPS14;RPL13A;LOC105604842;LOC101123533;RPS24;RPL32;MRPL21;LOC105607128;RPS7;RPLP1;RPS11;RPL10;RPL11;CMC1;RPL30;MRPL11;RPL38;RPL4;RPS17;RPL12;UBA52;LOC101123186;RPS3;FAU;novel.1438;RPS20;RPL23A;RPS29;MRPS18A;LOC101109155;RPL5;novel.574;RPL29;novel.2003;novel.3330;novel.283;novel.2327;RPL26L1;RPSA;LOC101111180;RPS27;LOC101118057;RPLP0;LOC101116132;novel.256;RPL39;LOC101110546;LOC101114033;LOC101109380;LOC101104787;RPS8;LOC101123112;RPL27;RPS3A;LOC105607745;RPS6;MRPL32;novel.1741;RPS2;MRPS15;LOC101103595;novel.1132;RPL6;RPL8;RPL27A;RPS5;RPS26-2;RPS27L;LOC101110758;LOC101119869;LOC101109545 |
| Alzheimer disease | LOC101115614;LOC443240;LOC443104;MAPK1;IRS2;MAP2K1;LOC101121024;ATF6;CSNK2A1;WNT5A;RB1CC1;novel.3599;LOC101104348;MAPK8;PSMD3;SLC39A9;LOC101109981;CTNNB1;novel.511;CSNK2A2;SLC39A13;LPL;novel.3624;LOC101119975;KIF5A;TNFRSF1A;FZD7;PIK3CA;GSK3B;LOC101109608;ATP2A2;AKT3;LOC101103959;NOS1;LOC105605934;AKT2;EIF2AK3;GRM5;novel.2701;LOC106991402;SLC39A3;PIK3R1;EIF2S1;CSNK1A1;ATP5A1;SDHD;LOC106991605;RTN4;GRIN1;BECN1;WNT9A;PSMD11;LRP6;novel.2043;WNT3A;FZD8;NRAS;ATP5B | ND6;ND5;LOC101103463;novel.3324;NDUFA2;NDUFA13;novel.368;LOC105606223;CYTB;NDUFB7;NDUFS5;ATF4;LOC101105179;COX3;COX1;ATP6;novel.1573;NDUFB3;UQCRH;novel.370;ATP8;NDUFB1;NDUFA3;NDUFB10;novel.371;NDUFA8;PSMC5;LOC101115773;novel.3901;novel.3902;LOC101116886;ATP5J;ATG2A;PSMD4;NDUFB9;NDUFA1;LOC101108778;LOC101108663;novel.3368;NDUFA11;novel.2849;ND2;HRAS;novel.1122;NDUFB11;LOC101110664;LOC101121538;LOC101108730;LOC101119721;novel.2303;PSMC3;novel.2048;novel.1423;COX2;NDUFS7;LOC101110066;novel.369;ND1;LOC101121420;LOC105609918;ATP5H;novel.2267;NDUFS8;novel.551;SLC39A8;SLC39A5;LOC105614854;PSMA8;NDUFB8;ND4;LOC101121285;novel.2733;novel.309;FZD10;APOE;DKK2;LOC101102327 |
| Amyotrophic lateral sclerosis | UBQLN1;LOC443104;ATF6;BCL2L1;ACTR1A;RB1CC1;POM121C;LOC101104348;PSMD3;RAB8A;NUP50;ATXN2L;DERL1;ERBB4;RAB1A;LOC101119975;KIF5A;TNFRSF1A;NUP62;LOC101109608;TPR;NUP153;DNAH2;LOC101103959;NOS1;TARDBP;EIF2AK3;CHMP2B;DNAH6;SQSTM1;SETX;EIF2S1;DNAL4;NUP58;ATP5A1;SDHD;GRIN1;BECN1;ACTB-2;NDC1;DNAI2;DNAH3;TOMM40L;PSMD11;NUP133;novel.2043;novel.1334;novel.2030;PFN2;ATP5B | ND6;ND5;LOC101103463;SOD1;novel.3324;NDUFA2;NDUFA13;novel.368;LOC105606223;CYTB;NDUFB7;NDUFS5;ATF4;LOC101105179;COX3;novel.158;COX1;ATP6;novel.1573;NDUFB3;UQCRH;novel.370;ATP8;NDUFB1;NDUFA3;NDUFB10;novel.371;NDUFA8;PSMC5;LOC101115773;novel.3901;novel.3902;PFN1;LOC101116886;ATP5J;ATG2A;PSMD4;NDUFB9;CCS;NDUFA1;LOC101108778;LOC101108663;novel.3368;NDUFA11;novel.2849;MAPK12;ND2;novel.1122;NDUFB11;LOC101110664;LOC101121538;LOC101108730;BAX;LOC101119721;novel.2303;PSMC3;novel.2048;novel.1423;COX2;NXF1;NDUFS7;LOC101110066;novel.369;ND1;LOC101121420;HDAC6;LOC105609918;ATP5H;CHCHD10;novel.2267;NDUFS8;novel.551;novel.3517;PSMA8;NDUFB8;ND4;LOC101121285;novel.2733;novel.309;LOC101102327 |
| Parkinson disease | LOC101115614;LOC443104;PRKACA;ADCY5;LOC101121024;ATF6;BCL2L1;LOC101104348;MAPK8;PSMD3;SLC39A9;UBE2G2;LOC101109981;novel.511;SLC39A13;LOC101119975;KIF5A;LOC101109608;PRKACB;UBE2L3;LOC101103959;novel.197;EIF2AK3;LOC106991402;LOC105606029;SLC39A3;GNAL;EIF2S1;ATP5A1;SDHD;LOC101102411;PSMD11;novel.2043;novel.2030;ATP5B | ND6;ND5;SOD1;novel.3324;NDUFA2;NDUFA13;novel.368;LOC105606223;CYTB;NDUFB7;NDUFS5;ATF4;LOC101105179;COX3;novel.158;COX1;ATP6;novel.1573;NDUFB3;UQCRH;LOC443297;novel.370;ATP8;NDUFB1;NDUFA3;NDUFB10;novel.371;NDUFA8;PSMC5;LOC101115773;novel.3901;novel.3902;RPS27A;LOC101116886;ATP5J;PSMD4;NDUFB9;NDUFA1;LOC101108778;LOC101108663;novel.3368;TXN;NDUFA11;novel.2849;ND2;novel.1122;NDUFB11;LOC101110664;LOC101121538;UBA52;LOC101108730;BAX;LOC101119721;novel.2303;PSMC3;novel.2048;novel.1423;COX2;NDUFS7;LOC101110066;novel.369;ND1;LOC101121420;LOC105609918;ATP5H;novel.2267;NDUFS8;HTRA2;novel.551;novel.3517;SLC39A8;SLC39A5;LOC105614854;PSMA8;NDUFB8;ND4;LOC101121285;novel.2733;novel.309;LOC101102327 |
| Huntington disease | LOC443104;KCNJ10;AP2B1;ACTR1A;TFAM;RB1CC1;LOC101104348;MAPK8;PSMD3;LOC101109981;novel.511;CREB3L2;POLR2A;LOC101119975;KIF5A;AP2A1;LOC101109608;DNAH2;LOC101103959;GRM5;DNAH6;RCOR1;AP2M1;DNAL4;ATP5A1;SDHD;GRIN1;BECN1;DNAI2;DNAH3;PSMD11;novel.2043;LOC101123026;CREBBP;ATP5B | LOC105604154;ND6;ND5;LOC101103463;SOD1;novel.3324;NDUFA2;NDUFA13;POLR2I;novel.368;LOC105606223;CYTB;NDUFB7;NDUFS5;LOC101105179;COX3;novel.158;COX1;ATP6;novel.1573;NDUFB3;UQCRH;novel.370;ATP8;NDUFB1;NDUFA3;NDUFB10;novel.371;NDUFA8;PSMC5;LOC101115773;novel.3901;POLR2J;novel.3902;LOC101116886;ATP5J;ATG2A;PSMD4;NDUFB9;NDUFA1;LOC101108778;LOC101108663;novel.3368;NDUFA11;novel.2849;ND2;novel.1122;NDUFB11;LOC101110664;LOC101121538;LOC101108730;BAX;LOC101119721;novel.2303;PSMC3;novel.2048;novel.1423;COX2;NDUFS7;LOC101110066;LOC105616906;novel.369;ND1;LOC101121420;LOC105609918;ATP5H;novel.2267;NDUFS8;novel.551;BBC3;PSMA8;CLTB;NDUFB8;ND4;LOC101121285;LOC106991563;novel.309;AP2S1;LOC101102327 |
| Prion disease | LOC443104;MAPK1;PRKACA;HSPA8;CSNK2A1;LOC101104348;MAPK8;PSMD3;NCAM1;LOC101109981;STIP1;CAV1;novel.511;CREB3L2;CSNK2A2;LOC101119975;LOC101121082;KIF5A;PIK3CA;GSK3B;LOC101109608;LOC494436;LOC101121333;PRKACB;LOC101103959;EIF2AK3;PIK3R1;EIF2S1;ATP5A1;EGR1;SDHD;GRIN1;PSMD11;novel.2043;LOC101123026;ATP5B | ND6;ND5;SOD1;novel.3324;NDUFA2;NDUFA13;novel.368;LOC105606223;CYTB;NDUFB7;NDUFS5;ATF4;LOC101105179;COX3;novel.158;COX1;ATP6;novel.1573;NDUFB3;UQCRH;novel.370;ATP8;NDUFB1;NDUFA3;NDUFB10;novel.371;NDUFA8;PSMC5;LOC101115773;novel.3901;novel.3902;LOC101116886;ATP5J;PSMD4;NDUFB9;NDUFA1;LOC101108778;LOC101108663;novel.3368;NDUFA11;novel.2849;MAPK12;ND2;novel.1122;NDUFB11;LOC101110664;LOC101121538;LOC101108730;BAX;LOC101119721;novel.2303;PSMC3;novel.2048;novel.1423;COX2;NDUFS7;LOC101110066;novel.369;LOC101106452;ND1;LOC101121420;LOC105609918;ATP5H;novel.2267;NDUFS8;RYR1;novel.551;PSMA8;NDUFB8;ND4;LOC101121285;novel.2733;novel.309;LOC101102327 |
| Thermogenesis | PRKACA;TSC1;ADCY5;ADCY9;LOC101104348;LOC101120042;CREB3L2;LOC101105425;ZNF217;SLC25A20;PRKACB;LOC101103154;novel.197;ADCY3;ZNF516;LOC105606029;ATP5A1;SDHD;ACTB-2;RPS6KA3;LOC101102411;LOC105615140;NRAS;ATP5B | ND6;ND5;ATP5I;novel.3324;NDUFA2;LOC106991149;NDUFA13;novel.368;LOC105606223;CYTB;NDUFB7;LOC101107153;NDUFS5;LOC101105179;COX3;COX1;ATP6;NDUFAF3;novel.1573;NDUFB3;UQCRH;C11H17orf89;novel.370;ATP8;NDUFB1;ATP5J2;NDUFA3;NDUFB10;novel.371;NDUFA8;LOC101112076;LOC101115773;novel.3901;novel.3902;LOC101116886;ATP5J;RPS6KB2;NDUFB9;NDUFA1;LOC101108778;LOC101108663;novel.3368;NDUFA11;novel.2849;MAPK12;ND2;HRAS;novel.1122;NDUFB11;LOC101110664;LOC101121538;LOC101119721;LOC101109345;novel.2048;novel.1423;COX2;novel.2327;NDUFS7;LOC101110066;novel.369;ND1;LOC101121420;LOC105609918;ATP5H;novel.2267;NDUFS8;novel.551;ATP5L;RPS6;NDUFB8;ND4;LOC101121285;RPS6KA1;novel.309;LOC101104176;LOC101102327 |
| Chemical carcinogenesis - reactive oxygen species | MAPK1;MAP2K1;FOXO3;ARNT;LOC101104348;MAPK8;LOC101109981;novel.511;ABL1;PDPK1;PIK3CA;AKT3;AKT2;LOC101114408;PIK3R1;ATP5A1;SDHD;EGFR;LOC106991605;ABL2;NRAS;ATP5B | ND6;ND5;SOD1;novel.3324;NDUFA2;NDUFA13;novel.368;LOC105606223;CYTB;NDUFB7;NDUFS5;LOC101105179;COX3;novel.158;COX1;ATP6;novel.1573;NDUFB3;UQCRH;novel.370;ATP8;NDUFB1;NDUFA3;NDUFB10;novel.371;NDUFA8;LOC101115773;novel.3901;novel.3902;LOC101116886;ATP5J;NDUFB9;NDUFA1;LOC101108778;LOC101108663;novel.3368;NDUFA11;novel.2849;MAPK12;LOC101114663;ND2;HRAS;novel.1122;NDUFB11;LOC101110664;LOC101121538;LOC101119721;novel.2048;novel.1423;COX2;NDUFS7;LOC101110066;novel.369;LOC101106452;ND1;GSTT2B;LOC101121420;LOC105609918;ATP5H;novel.2267;NDUFS8;novel.551;PLD2;SLC26A6;NDUFB8;ND4;LOC101121285;novel.309;AS3MT;LOC101102327 |
| Diabetic cardiomyopathy | GFPT1;PDHA1;LOC101104348;LOC101117153;MAPK8;TGFBR2;LOC101109981;novel.511;PARP1;PIK3CA;novel.2897;GSK3B;ATP2A2;AKT3;MMP2;AKT2;PPP1CC;PIK3R1;ATP5A1;SDHD;LOC106991605;TBC1D4;ATP5B | ND6;ND5;novel.3324;NDUFA2;NDUFA13;novel.368;LOC105606223;CYTB;NDUFB7;NDUFS5;LOC101105179;COX3;COX1;ATP6;novel.1573;NDUFB3;UQCRH;novel.370;ATP8;NDUFB1;NDUFA3;NDUFB10;novel.371;NDUFA8;LOC101115773;novel.3901;novel.3902;LOC101116886;ATP5J;NDUFB9;NDUFA1;LOC101108778;LOC101108663;novel.3368;NDUFA11;novel.2849;MAPK12;ND2;novel.1122;NDUFB11;LOC101110664;LOC101121538;LOC101119721;novel.2048;novel.1423;COX2;NDUFS7;LOC101110066;novel.369;LOC101106452;ND1;LOC101121420;LOC105609918;ATP5H;LOC101117953;novel.2267;NDUFS8;novel.551;MPC1;NDUFB8;ND4;LOC101121285;novel.309;LOC101102327 |
| Endocytosis | IQSEC1;AP2B1;RAB35;HSPA8;ARF4;EHD3;LOC105606122;WIPF2;TGFBR2;RAB5C;IGF2R;RAB11B;CDC42;RAB8A;CYTH3;CAV1;ARFGEF2;ARF3;ZFYVE9;LOC106991303;GIT1;SNX2;KIF5A;RAB10;LOC101117289;AP2A1;AGAP1;LOC494436;GRK6;MVB12B;IQSEC3;DNAJC6;ARF6;IGF1R;SH3GL2;ARFGEF1;CHMP2B;IQSEC2;AP2M1;LOC106991323;VPS37B;ACAP2;EPN1;PARD6B;LOC101103187;EGFR;PDGFRA;ARFGAP2;PSD3;VPS26B;PML;CAPZA1;RAB11FIP4;RAB11FIP1;EPN2 | ACAP1;CHMP2A;HRAS;VPS28;PSD2;novel.170;LOC105616906;ARAP3;LOC101119385;GRK4;ACAP3;ARPC1B;LOC106990244;ASAP3;PLD2;CLTB;novel.1283;LOC106991563;AP2S1;LOC101105609 |
| Oxidative phosphorylation | LOC101104348;ATP6V1D;LOC101105425;ATP6AP1;ATP5A1;ATP6V0A1;SDHD;ATP4A;ATP5B | ND6;ND5;ATP5I;novel.3324;NDUFA2;NDUFA13;novel.368;LOC105606223;CYTB;NDUFB7;LOC101107153;NDUFS5;LOC101105179;COX3;COX1;ATP6;novel.1573;NDUFB3;UQCRH;novel.370;ATP8;NDUFB1;ATP5J2;NDUFA3;NDUFB10;novel.371;NDUFA8;LOC101115773;novel.3901;novel.3902;LOC101116886;ATP5J;NDUFB9;NDUFA1;LOC101108778;LOC101108663;novel.3368;NDUFA11;novel.2849;ND2;novel.1122;NDUFB11;LOC101110664;LOC101121538;LOC101119721;novel.2048;novel.1423;COX2;NDUFS7;LOC101110066;novel.369;ND1;LOC101121420;LOC105609918;ATP5H;novel.2267;NDUFS8;novel.551;ATP5L;NDUFB8;ND4;LOC101121285;novel.309;LOC101102327 |
| Non-alcoholic fatty liver disease | IRS2;MAPK8;GSK3A;CDC42;CEBPA;TNFRSF1A;PIK3CA;GSK3B;ADIPOR2;AKT3;AKT2;EIF2AK3;PIK3R1;EIF2S1;SDHD;novel.588;LOC106991605;MLXIP;novel.587;LOC105615140;BCL2L11 | LOC101103463;NDUFA2;NDUFA13;novel.368;CYTB;NDUFB7;NDUFS5;ATF4;LOC101105179;COX3;COX1;NDUFB3;UQCRH;NDUFB1;NDUFA3;NDUFB10;NDUFA8;LOC101115773;novel.3901;LOC101116886;NDUFB9;NDUFA1;LOC101108778;LOC101108663;novel.3368;NDUFA11;novel.2849;MAPK12;novel.1122;NDUFB11;LOC101110664;LOC101121538;BAX;LOC101119721;novel.2048;COX2;NDUFS7;LOC101110066;novel.369;LOC101121420;LOC105609918;NDUFS8;novel.551;NDUFB8;LOC101121285;LOC101102327 |
| Retrograde endocannabinoid signaling | MAPK1;PRKACA;KCNJ9;ADCY5;ADCY9;LOC101104348;MAPK8;GNG4;DAGLA;PRKACB;novel.197;ADCY3;GRM5;LOC105606029;GABRR2;GABRR1;GABRG1;LOC101123026 | ND6;ND5;novel.3324;NDUFA2;NDUFA13;NDUFB7;NDUFS5;novel.1573;NDUFB3;novel.370;NDUFB1;NDUFA3;NDUFB10;novel.371;NDUFA8;LOC101115773;novel.3902;NDUFB9;NDUFA1;novel.3368;NDUFA11;MAPK12;ND2;NDUFB11;NDUFS7;ND1;NDUFS8;novel.551;LOC101103602;GNG11;NDUFB8;GNG5;ND4;novel.2733 |
| Endocrine resistance | MAPK1;PRKACA;ADCY5;MAP2K1;ADCY9;MED1;CARM1;MAPK8;LOC101121082;PIK3CA;ESR2;LOC101121333;CDKN1A;PRKACB;AKT3;MMP2;IGF1R;novel.197;AKT2;ADCY3;LOC105606029;PIK3R1;LOC101103187;EGFR;LOC106991605;LOC101102411;NRAS | RPS6KB2;MAPK12;HRAS;BAX;novel.2641;LOC105603317;SHC2 |
| Cardiac muscle contraction | CACNG7;SLC8A2;SLC9A1;CACNB4;ATP2A2;ATP1B2;LOC101118433;ATP1A1;CACNA2D2;ASPHD2 | novel.368;CYTB;LOC101105179;COX3;COX1;UQCRH;novel.3901;LOC101116886;LOC101108778;LOC101108663;novel.2849;novel.1122;LOC101110664;LOC101121538;LOC101119721;novel.2048;COX2;LOC101110066;novel.369;LOC101121420;LOC105609918;LOC101121285;LOC101102327 |
| Progesterone-mediated oocyte maturation | MAPK1;PRKACA;ADCY5;MAP2K1;ADCY9;MAPK8;CPEB3;PIK3CA;PRKACB;CCNJ;AKT3;FZR1;IGF1R;novel.197;AKT2;ADCY3;LOC105606029;PIK3R1;LOC105611974;LOC106991605;RPS6KA3 | ANAPC11;novel.742;ANAPC16;ANAPC4;ANAPC15;MAPK12;MAD2L2;KIF22;LOC101108105;PLK1;RPS6KA1;CDC25B |
| Longevity regulating pathway - multiple species | IRS2;PRKACA;ADCY5;FOXO3;HSPA8;ADCY9;CLPB;PIK3CA;EIF4EBP2;LOC494436;PRKACB;AKT3;IGF1R;novel.197;AKT2;ADCY3;LOC105606029;PIK3R1;LOC106991605;LOC105615140;NRAS | SOD1;novel.158;RPS6KB2;HRAS |
