# Supplementary material for: Transcriptomic regulation of the hypothalamic-pituitary axis by GnRH immunization in Xizang sheep
Source: Anim Biotechnol. 2026 Feb 19;37(1):2631819. doi: 10.1080/10495398.2026.2631819 (PMC12927403; doi:10.1080/10495398.2026.2631819)
Supplement: Additional Table1 GO and KEGG Pathway Enrichment Analysis of Pituitary Differentially Expressed Genes.docx [file LABT_A_2631819_SM2768.docx]

GO Enrichment Pathways and Differentially Expressed Genes in pituitary (IM vs. CON)

| GO Enrichment Pathways | Up Genes | Down Genes |
| --- | --- | --- |
| myofibril | TCAP;MYOZ1;TNNI2;NRAP;ACTA1;LOC443471;XIRP2;PDLIM3;LOC442994;TMOD4;LDB3;TNNC1;TNNT1;ACTN3;CSRP1;ACTN2;C25H10orf71;RPL15;KLHL41;CSRP3;MYL2;TTN;FHL2;TNNT3;SYNPO;MYH7B;DES;MYOM1;MYH15;PDLIM1;LOC101123118 | ANK1;CACNA1D |
| contractile fiber | TCAP;MYOZ1;TNNI2;NRAP;ACTA1;LOC443471;XIRP2;PDLIM3;LOC442994;TMOD4;LDB3;TNNC1;TNNT1;ACTN3;CSRP1;ACTN2;C25H10orf71;RPL15;KLHL41;CSRP3;MYL2;TTN;FHL2;TNNT3;SYNPO;MYH7B;DES;MYOM1;MYH15;PDLIM1;LOC101123118 | ANK1;CACNA1D |
| ribosomal subunit | novel.5586;RPL8;LOC106991767;RPL35;RPS6;novel.4029;RPL28;LOC101109132;RPL15;RPS5;RPS27;LOC101113219;RPS14;RPLP0;novel.4178;RPSA;RPS26;RPL29;novel.58;GNB2L1;novel.4488;RPS2;RPS29;LOC101102096;LOC101107098;RPL3L;RPL18;novel.4146;LOC101107216;RPS15;novel.782 | novel.3794;RPL10A |
| structural constituent of ribosome | novel.5586;RPL8;LOC106991767;RPL35;RPS6;novel.4029;RPL28;LOC101109132;RPL15;RPS5;RPS27;LOC101113219;RPS14;RPLP0;novel.4178;RPSA;RPS26;RPL29;novel.58;novel.4488;RPS2;RPS29;LOC101110941;LOC101102096;LOC101107098;RPL3L;RPL18;novel.4146;LOC101107216;RPS15;novel.782 | novel.3794;RPL10A |
| cytosolic ribosome | RPL8;LOC106991767;RPL35;RPS6;novel.4029;RPL28;LOC101109132;RPL15;RPS5;RPS27;LOC101113219;RPS14;RPLP0;novel.4178;RPSA;RPS26;RPL29;novel.58;GNB2L1;novel.4488;RPS2;RPS29;LOC101102096;LOC101107098;RPL3L;RPL18;LOC101107216;RPS15;novel.782 | novel.3794;RPL10A |
| sarcomere | TCAP;MYOZ1;TNNI2;NRAP;ACTA1;LOC443471;XIRP2;PDLIM3;TMOD4;LDB3;TNNC1;TNNT1;ACTN3;CSRP1;ACTN2;C25H10orf71;RPL15;KLHL41;CSRP3;MYL2;TTN;FHL2;TNNT3;SYNPO;DES;MYOM1;PDLIM1;LOC101123118 | ANK1;CACNA1D |
| cytoplasmic translation | RPL8;LOC106991767;novel.4029;LOC101109132;RPL15;RPS5;EIF3G;RPS27;LOC101113219;RPS14;EIF4B;RPLP0;novel.4178;RPSA;RPS26;RPL29;GNB2L1;novel.4488;RPS29;LOC101102096;LOC101107098;novel.2232;RPL18;LOC101107216 | novel.1689;RPL10A;LOC101122488 |
| muscle contraction | VEGFB;TCAP;TNNI2;MYBPC2;TPM2;ACTA1;PABPN1;LOC442994;TMOD4;TNNC1;TNNT1;ATP2A1;ACTN3;KLHL41;CSRP3;MYL2;TTN;TNNT3;MYOM1;CACNA1G;MYLK2;FGF13 | KCNMA1;CACNA1D;CACNA2D1;SCN7A |
| respirasome | LOC105616860;CYTB;ND4L;COX3;COX1;novel.4917;ND4;novel.586;ND2;novel.4919;COX2;novel.5553;ND6;novel.585;novel.2366;novel.583;ND3 | LOC101104348 |
| small ribosomal subunit | novel.5586;RPS6;LOC101109132;RPS5;RPS27;LOC101113219;RPS14;RPSA;RPS26;novel.58;GNB2L1;novel.4488;RPS2;RPS29;LOC101102096;novel.4146;LOC101107216;RPS15 | -- |
| oxidative phosphorylation | LOC105616860;ND4L;COX3;COX1;novel.4917;ATP6;ACTN3;ND4;novel.586;ND2;novel.5553;ND6;novel.585;novel.2366;novel.583;ND3 | LOC101104348 |
| oxidoreduction-driven active transmembrane transporter activity | CYTB;ND4L;COX3;COX1;novel.4917;ND4;novel.586;ND2;novel.4919;COX2;novel.5553;ND6;novel.585;novel.2366;novel.583;ND3 | LOC101104348 |
| electron transfer activity | CYTB;ND4L;COX3;COX1;novel.4917;ND4;novel.586;ND2;novel.4919;COX2;novel.5553;ND6;novel.585;novel.2366;novel.583;ND3 | LOC101104348 |
| cytosolic small ribosomal subunit | RPS6;LOC101109132;RPS5;RPS27;LOC101113219;RPS14;RPSA;RPS26;novel.58;GNB2L1;novel.4488;RPS2;RPS29;LOC101102096;LOC101107216;RPS15 | -- |
| respiratory chain complex | LOC105616860;CYTB;COX3;COX1;novel.4917;ND4;novel.586;ND2;novel.4919;COX2;novel.5553;ND6;novel.585;novel.2366;novel.583;ND3 | -- |
| NADH dehydrogenase (ubiquinone) activity | ND4L;ND4;novel.586;ND2;novel.5553;ND6;novel.2366;ND3 | LOC101104348 |
| NADH dehydrogenase (quinone) activity | ND4L;ND4;novel.586;ND2;novel.5553;ND6;novel.2366;ND3 | LOC101104348 |
| NADH dehydrogenase activity | ND4L;ND4;novel.586;ND2;novel.5553;ND6;novel.2366;ND3 | LOC101104348 |
| NAD(P)H dehydrogenase (quinone) activity | ND4L;ND4;novel.586;ND2;novel.5553;ND6;novel.2366;ND3 | LOC101104348 |
| oxidoreductase activity, acting on NAD(P)H, quinone or similar compound as acceptor | ND4L;ND4;novel.586;ND2;novel.5553;ND6;novel.2366;ND3 | LOC101104348 |
| myofilament | TNNI2;ACTA1;TMOD4;TNNC1;TNNT1;TTN;TNNT3;MYOM1 | -- |
| striated muscle thin filament | TNNI2;ACTA1;TMOD4;TNNC1;TNNT1;TTN;TNNT3 | -- |

GO Enrichment Pathways and Differentially Expressed Genes in pituitary (IM vs. SN)

| GO Enrichment Pathways | Up Genes | Down Genes |
| --- | --- | --- |
| actin binding | TPM2;TPM4;novel.4676;MYH7;TNNI2;LOC101114849;PDLIM3;MYBPC2;LDB3;PDLIM7;EVL;AIF1;MKL1;TMSB4X;MISP;TMOD4;PDLIM5;MYOM1;LOC106991842;LOC106991557;MICAL3;GMFB;PSTPIP1;XIRP2;FAM107A;TNNC1;GAS7;MYOM2;AIF1L;USH1C;novel.5434;TTN;SNCA;TNNI1;NRAP;PARVG;DAAM2;ACTN2;CROCC;KLHL5;SYNE1;SMTN;ACTN3;MYH7B;NEXN;CSRP3;LOC443471;novel.5655;SPTBN5;SYNPO2;LOC101106384;MARCKSL1;LOC106991083;ANKRD1;LOC101120817;LOC101121750;ANLN;INF2;SHTN1;novel.4405;PPP1R42;LOC101106245;DIAPH2;MYOT;MYOZ1;MOBP;novel.2453;MYBPC1;ERMN;LOC101106517;CTNNA3;MYOZ2;LSP1;CNN1;LOC442994 | KCNMA1;MLLT4;SNTB2;ACTN1;ACTN4;LOC101106728;CDK5R2;AFAP1;SLC6A4;SHROOM2;TRPM7;LOC101104133;TMEM201;CORO1B;EPB41;FHDC1;HOOK1;MYO5C;GAS2;FMN1;novel.1365;novel.2910;ABLIM2;novel.5769;novel.4674;novel.5227;ARC;novel.1871;PLS1 |
| structural constituent of ribosome | novel.5586;RPL4;RPL38;novel.5266;novel.2912;RPL26;RPL37;LOC106991767;RPL31;RPS25;RPS19;MRPS2;RPL22;LOC101123279;RPL5;RPL9;LOC101114033;novel.1337;RPS6;RPL3L;LOC101110998;RPL24;RPL30;LOC105604842;RPL32;LOC106990192;novel.1700;RPS21;RPLP1;RPL39;LOC101103316;RPL35A;RPL28;RPS12;RPLP0;RPS262;LOC105606185;RPS11;RPL8;LOC101116132;novel.4029;RPL29;RPL15;novel.782;RPL102;RPS5;RPL18A;novel.435;novel.1285;RPL35;RPS29;novel.1387;RPS14;RPL10;RPS16;RPS2;RPS26;RPSA;RPL18;RPS27;LOC101110941;LOC106990113;LOC101107216;RPS15;LOC101102096;novel.4178;LOC101110758;novel.4101;novel.4488;LOC101105336;LOC105610139;novel.58;novel.1798;novel.3542;novel.692;LOC101107098;LOC101109545 | novel.4396;novel.645;LOC106991948;novel.3151;novel.3794;RPL10A |
| ribosomal subunit | novel.5586;RPL4;RPL38;novel.5266;novel.2912;RPL26;RPL37;LOC106991767;RPL31;RPS25;RPS19;MRPS2;LOC101123279;RPL5;RPL9;LOC101114033;novel.1337;RPS6;RPL3L;LOC101110998;novel.3757;RPL24;RPL30;LOC105604842;RPL32;novel.1700;RPS21;RPLP1;RPL39;LOC101103316;RPL35A;RPL28;RPS12;RPLP0;RPS262;RPS11;RPL8;LOC101116132;novel.4029;RPL29;RPL15;novel.782;RPL102;RPS5;RPL18A;novel.435;RPL35;RPS29;novel.1387;RPS14;RPL10;RPS16;RPS2;RPS26;RPSA;RPL18;RPS27;LOC106990113;LOC101107216;RPS15;GNB2L1;LOC101102096;novel.4178;LOC101110758;novel.4101;novel.4488;LOC101105336;LOC105610139;novel.58;novel.1798;novel.3542;novel.692;LOC101107098;LOC101109545 | novel.4396;DDX3X;novel.645;LOC106991948;novel.3151;novel.3794;RPL10A |
| cytosolic ribosome | RPL4;RPL38;novel.5266;novel.2912;RPL26;RPL37;LOC106991767;RPL31;RPS25;RPS19;LOC101123279;RPL5;RPL9;LOC101114033;novel.1337;RPS6;RPL3L;LOC101110998;RPL24;RPL30;LOC105604842;RPL32;novel.1700;RPS21;RPLP1;RPL39;LOC101103316;RPL35A;RPL28;RPS12;RPLP0;RPS262;RPS11;RPL8;LOC101116132;novel.4029;RPL29;RPL15;novel.782;RPL102;RPS5;RPL18A;novel.435;RPL35;RPS29;novel.1387;RPS14;RPL10;RPS16;RPS2;RPS26;RPSA;RPL18;RPS27;LOC106990113;LOC101107216;RPS15;GNB2L1;LOC101102096;novel.4178;LOC101110758;novel.4101;novel.4488;LOC101105336;LOC105610139;novel.58;novel.1798;novel.3542;novel.692;LOC101107098;LOC101109545 | novel.4396;DDX3X;novel.645;LOC106991948;novel.3151;novel.3794;RPL10A |
| transporter complex | novel.4917;ND6;CYTB;ND5;novel.2366;novel.5553;COX1;novel.4919;novel.584;novel.586;CATSPER3;TEX40;TIMM8A;SLC26A6;GRIN2D;ND2;ND1;WDR93;LOC101107658;LOC105608581;ND4;CACNG2;CACNA1G;novel.4085;LOC101108953;KCNAB1;CATSPER4;SHISA8;FKBP1B;LRRC8E;CNIH2;KCNAB2;RYR2;KCNG1;GRIN3B;DPP10 | KCNMA1;GABRR2;ATP1B2;ATP8B2;TTYH3;GABRA1;ATP1A3;ABCB8;DLG4;CHRNA2;CACNA2D2;LRRC8A;LOC101116843;SCN4B;LOC105606136;KCNC3;ATP11A;KCNJ3;novel.5123;CACNA1C;novel.929;NDUFS4;CACNA1E;KCNQ4;GRIN2A;CNGB3;ATP11C;MICU3;LOC101106522;LOC101122774;CACNA2D1;CHRNB2;GABRA5;ATP1A1;LOC105604604;novel.4557;HCN1;KCNV2;LOC106991592;LRRC38;ATP8A1;SCN2B |
| transport vesicle | RNF112;SEPT4;COPZ2;AP1G2;EXOC3L1;novel.5707;BGN;SNCA;RAB26;SYT13;LOC105615749;DPYSL3;SNCAIP;SYNDIG1;CNIH2;DGKI;LOC101113807;ANXA13 | STX3;ATP6V0A1;UNC13A;SYT5;VGF;ATP6V0C;SLC17A6;ATP6AP1;MCFD2;TGOLN2;PCSK1;SNTB2;SYT4;SYT6;SEMA4C;SEC23A;SCAMP5;SCG3;C14H16orf70;LOC105606122;DLG4;CHGB;RAB3C;SV2B;SEC24B;CLCN4;GALNT15;LOC101104133;AP2A1;RAB1A;ITPR1;ATP6V1A;BSN;novel.5433;SH3GL2;SYNGR1;AP1M1;RAB3A;PTPRS;CLCN3;GRIN2A;CHGA;SLC17A7;novel.1481;novel.3464;novel.4090;RAB3IP;MCTP2;SYT1;DISC1;WFS1;SYN3;PCSK2;novel.4091 |
| primary active transmembrane transporter activity | novel.4917;ND6;CYTB;ND5;novel.2366;novel.5553;COX1;novel.4919;novel.584;COX2;COX3;novel.586;novel.5551;ND2;ND1;novel.583;ND4;ND4L;ATP2A1;LOC101102327;novel.1353;ABCB1;LOC101106131 | ATP6V0A1;LOC101104348;ATP6V0C;ATP2B2;ATP1A3;ABCB8;ATP6V0B;LOC101116843;ATP6V1A;novel.4565;novel.4566;ABCB10;NDUFS4;TAP2;ABCG1;ABCC5;LOC105605850;ATP1A1;LOC101121256;ABCB12;LOC105606000;ATP6V1D;LOC101106534;LOC101112460;ATP8A1 |
| cytosolic small ribosomal subunit | novel.5266;RPS25;RPS19;RPS6;LOC105604842;RPS21;LOC101103316;RPS12;RPS262;RPS11;LOC101116132;RPS5;novel.435;RPS29;RPS14;RPS16;RPS2;RPS26;RPSA;RPS27;LOC101107216;RPS15;GNB2L1;LOC101102096;LOC101110758;novel.4488;novel.58;novel.3542 | DDX3X |
| synaptic vesicle membrane | SNCA;RAB26;SYNDIG1;DGKI | ATP6V0A1;UNC13A;SYT5;ATP6V0C;SLC17A6;ATP6AP1;SYT4;SYT6;SEMA4C;SCAMP5;C14H16orf70;SV2B;LOC101104133;BSN;SH3GL2;SYNGR1;RAB3A;PTPRS;CLCN3;SLC17A7;novel.1481;MCTP2;SYT1;WFS1;SYN3 |
| exocytic vesicle membrane | SNCA;RAB26;SYNDIG1;DGKI | ATP6V0A1;UNC13A;SYT5;ATP6V0C;SLC17A6;ATP6AP1;SYT4;SYT6;SEMA4C;SCAMP5;C14H16orf70;SV2B;LOC101104133;BSN;SH3GL2;SYNGR1;RAB3A;PTPRS;CLCN3;SLC17A7;novel.1481;MCTP2;SYT1;WFS1;SYN3 |
| electron transfer activity | novel.4917;ND6;CYTB;ND5;novel.2366;novel.5553;COX1;novel.4919;novel.584;COX2;COX3;novel.586;novel.5551;FDX1L;ND2;ND1;novel.583;ND4;ND4L;LOC101102327;AIFM2;LOC101106131 | LOC101104348;LOC101116843;NDUFS4;AOC2 |
| oxidoreduction-driven active transmembrane transporter activity | novel.4917;ND6;CYTB;ND5;novel.2366;novel.5553;COX1;novel.4919;novel.584;COX2;COX3;novel.586;novel.5551;ND2;ND1;novel.583;ND4;ND4L;LOC101102327;LOC101106131 | LOC101104348;LOC101116843;NDUFS4 |
| polysomal ribosome | RPL38;RPL31;EIF3H;RPL24;RPL30;RPL32;RPS21;RPL39;LOC101103316;RPS262;RPL8;RPL18A;RPS29;RPS26;RPL18;novel.4178;LOC105610139 | LARP1;RPL10A |
| actinin binding | novel.4676;PDLIM3;LDB3;PDLIM7;PDLIM5;XIRP2;TTN;NRAP;CSRP3;SYNPO2;LOC101106245;MYOT | ACTN1;CACNA1C;novel.4674 |
| oxidoreductase activity, acting on NAD(P)H, quinone or similar compound as acceptor | ND6;ND5;novel.2366;novel.5553;novel.586;ND2;ND1;ND4;ND4L;CBR3;AIFM2 | LOC101104348;LOC101116843;NDUFS4 |
| NAD(P)H dehydrogenase (quinone) activity | ND6;ND5;novel.2366;novel.5553;novel.586;ND2;ND1;ND4;ND4L;CBR3 | LOC101104348;LOC101116843;NDUFS4 |
| chaperone-mediated autophagy | LOC100125610;novel.3614;GFAP;SNCA;LOC101120236;EEF1A1;novel.659;LOC101114018;SYNPO2;LOC101102857 | LOC101114987;HSPA8 |
| NADH dehydrogenase (ubiquinone) activity | ND6;ND5;novel.2366;novel.5553;novel.586;ND2;ND1;ND4;ND4L | LOC101104348;LOC101116843;NDUFS4 |
| NADH dehydrogenase (quinone) activity | ND6;ND5;novel.2366;novel.5553;novel.586;ND2;ND1;ND4;ND4L | LOC101104348;LOC101116843;NDUFS4 |
| NADH dehydrogenase activity | ND6;ND5;novel.2366;novel.5553;novel.586;ND2;ND1;ND4;ND4L | LOC101104348;LOC101116843;NDUFS4 |

GO Enrichment Pathways and Differentially Expressed Genes in pituitary (SN vs. CON)

| GO Enrichment Pathways | Up Genes | Down Genes |
| --- | --- | --- |
| generation of precursor metabolites and energy | JMJD8;LOC106991605;SLC25A22;PPARD;LOC105616860;PGAM1;MDH1;MDH2;ATP5B;LOC101114294;novel.5484;novel.2399;novel.407;PPP1R3D;LOC101102824;H6PD | ND6;ND5;novel.5553;novel.4917;ATP6;novel.4919;CYTB;novel.2366;ND1;novel.584;novel.586;NUPR1;COX1;COX3;novel.5551;UCN;PKLR;ND2 |
| transmembrane transporter complex | ATP1B2;TTYH3;GABRR2;KCNF1;ATP1A3;KCNC3;GABRA1;LRRC38;novel.5123;LOC106991592;novel.929;SCN2B;KCNG4;LOC101110253 | ND6;ND5;novel.5553;novel.4917;novel.4919;CYTB;novel.2366;ND1;novel.584;CATSPER3;novel.586;CACNG2;COX1;SCN7A;CHRNA5;ND2;TEX40 |
| active ion transmembrane transporter activity | SLC6A11;SLC25A22;SLC9A1;ATP1A3;SLC17A7;SLC6A1;SLC24A3;SLC26A11;CLCN3;ATP5B;ATP6V1D;SLC24A2;SLC12A5;SLC1A2;SLC10A1;SLC36A1;SLC22A18;SLCO1A2;LOC101106534;LOC101109981 | novel.4917;novel.4919;CYTB;COX2;novel.584;SLC9A5;COX1;COX3;novel.5551 |
| energy derivation by oxidation of organic compounds | LOC106991605;SLC25A22;LOC105616860;MDH1;MDH2;ATP5B;novel.5484;novel.2399;PPP1R3D | ND6;ND5;novel.5553;novel.4917;ATP6;novel.4919;CYTB;novel.2366;ND1;novel.584;novel.586;NUPR1;COX1;COX3;novel.5551;UCN;ND2 |
| secondary active transmembrane transporter activity | SLC6A11;SLC25A22;SLC9A1;ATP1A3;SLC25A10;SLC6A17;SLC17A7;SLC7A5;SLC6A1;SLC24A3;SLC26A11;CLCN3;SLC24A2;SLC12A5;SLC1A2;KIAA1919;SLC1A4;SLC10A1;SLC16A6;SLC36A1;SLC22A18;SLCO1A2;LOC101109981 | SLC47A2;SLC9A5;SLC16A8 |
| cellular respiration | SLC25A22;LOC105616860;MDH1;MDH2;ATP5B;novel.2399 | ND6;ND5;novel.5553;novel.4917;ATP6;novel.4919;CYTB;novel.2366;ND1;novel.584;novel.586;NUPR1;COX1;COX3;novel.5551;UCN;ND2 |
| primary active transmembrane transporter activity | ATP1A3;ATP5B;ATP6V1D;LOC105605772;LOC101106534 | ND6;ND5;novel.5553;novel.4917;novel.4919;CYTB;novel.2366;COX2;ND1;novel.584;novel.586;novel.1353;COX1;COX3;novel.5551;ND2 |
| inner mitochondrial membrane protein complex | TIMM22;LOC105616860;ATP5B;LOC106991907 | ND6;ND5;novel.5553;novel.4917;ATP6;novel.2366;COX2;ND1;novel.584;novel.586;COX1;COX3;ATP8;novel.5551;LOC101121949;ND2 |
| proton transmembrane transporter activity | SLC25A22;SLC9A1;SLC17A7;CLCN3;ATP5B;ATP6V1D;SLC36A1;SLC22A18;HVCN1 | novel.4917;ATP6;novel.4919;CYTB;COX2;novel.584;SLC9A5;COX1;COX3;ATP8;novel.5551 |
| aerobic respiration | LOC105616860;MDH1;MDH2;ATP5B | ND6;ND5;novel.5553;novel.4917;ATP6;novel.2366;ND1;novel.584;novel.586;NUPR1;COX1;COX3;novel.5551;UCN;ND2 |
| respirasome | LOC105616860 | ND6;ND5;novel.5553;novel.4917;novel.4919;CYTB;novel.2366;COX2;LOC101118736;ND1;novel.584;novel.586;COX1;COX3;novel.5551;ND2 |
| oxidative phosphorylation | LOC105616860;ATP5B | ND6;ND5;novel.5553;novel.4917;ATP6;novel.2366;ND1;novel.584;novel.586;NUPR1;COX1;COX3;novel.5551;ND2 |
| synaptic vesicle membrane | SH3GL2;SCAMP5;ATP6AP1;RAB11B;VAMP2;LOC101104133;UNC13A;SLC6A17;SLC17A7;SYT5;CLCN3;novel.1481;SYT6;SYNGR1;SYT1;WFS1 | -- |
| exocytic vesicle membrane | SH3GL2;SCAMP5;ATP6AP1;RAB11B;VAMP2;LOC101104133;UNC13A;SLC6A17;SLC17A7;SYT5;CLCN3;novel.1481;SYT6;SYNGR1;SYT1;WFS1 | -- |
| respiratory chain complex | LOC105616860 | ND6;ND5;novel.5553;novel.4917;novel.4919;CYTB;novel.2366;COX2;ND1;novel.584;novel.586;COX1;COX3;novel.5551;ND2 |
| electron transfer activity | LOC101102211 | ND6;ND5;novel.5553;novel.4917;novel.4919;CYTB;novel.2366;COX2;ND1;novel.584;novel.586;COX1;COX3;novel.5551;ND2 |
| oxidoreduction-driven active transmembrane transporter activity | -- | ND6;ND5;novel.5553;novel.4917;novel.4919;CYTB;novel.2366;COX2;ND1;novel.584;novel.586;COX1;COX3;novel.5551;ND2 |
| mitochondrial respirasome | LOC105616860 | ND6;ND5;novel.5553;novel.4917;novel.2366;COX2;ND1;novel.584;novel.586;COX1;COX3;novel.5551;ND2 |
| NADH dehydrogenase (ubiquinone) activity | -- | ND6;ND5;novel.5553;novel.2366;ND1;novel.586;ND2 |
| NADH dehydrogenase (quinone) activity | -- | ND6;ND5;novel.5553;novel.2366;ND1;novel.586;ND2 |

KEGG Enrichment Pathways and Differentially Expressed Genes in pituitary (IM vs. CON)

| KEGG Enrichment Pathways | Up Genes | Down Genes |
| --- | --- | --- |
| Coronavirus disease - COVID-19 | MAPK13;RPL8;LOC106991767;RPL35;RPS6;novel.4029;RPL28;LOC101109132;RPL15;RPS5;RPS27;LOC101113219;RPS14;RPLP0;novel.4178;RPSA;RPS26;RPL29;novel.58;FOS;novel.4488;RPS2;RPS29;LOC101110941;LOC101102096;LOC101107098;RPL3L;CFB;RPL18;novel.4146;LOC101107216;RPS15;MYD88;PRKCA | ADAM17;RPL10A |
| Pathways of neurodegeneration - multiple diseases | SNCAIP;ATP8;MAPK13;CYTB;ND4L;COX3;COX1;novel.4917;ATP6;CAMK2A;GRIN2D;ATP2A1;LOC101116286;ND4;novel.586;ND2;novel.4919;COX2;LOC101119975;novel.5553;ND6;LOC443104;novel.585;novel.2366;ATP5G2;novel.583;PRKCA;CHRM1;ND3 | ITPR1;GRIN2A;LOC101104348;CACNA1D;LOC101104288;novel.4092 |
| Alzheimer disease | ATP8;CYTB;ND4L;COX3;COX1;novel.4917;ATP6;GRIN2D;ATP2A1;LOC101116286;ND4;novel.586;ND2;novel.4919;COX2;LOC101119975;novel.5553;ND6;LOC443104;novel.585;novel.2366;ATP5G2;novel.583;CHRM1;ND3 | ITPR1;GRIN2A;LOC101104348;CACNA1D;ADAM17;novel.4092;SLC39A8 |
| Ribosome | novel.5586;RPL8;LOC106991767;RPL35;RPS6;novel.4029;RPL28;LOC101109132;RPL15;RPS5;RPS27;LOC101113219;RPS14;RPLP0;novel.4178;RPSA;RPS26;RPL29;novel.58;novel.4488;RPS2;RPS29;LOC101110941;LOC101102096;LOC101107098;RPL3L;RPL18;novel.4146;LOC101107216;RPS15 | RPL10A |
| Prion disease | ATP8;MAPK13;CYTB;ND4L;COX3;COX1;novel.4917;ATP6;GRIN2D;LOC101116286;ND4;novel.586;ND2;novel.4919;COX2;LOC101119975;novel.5553;ND6;LOC443104;novel.585;novel.2366;GRIN3B;ATP5G2;novel.583;ND3 | ITPR1;GRIN2A;LOC101104348;CACNA1D;novel.4092 |
| Parkinson disease | SNCAIP;ATP8;CYTB;ND4L;COX3;COX1;novel.4917;ATP6;CAMK2A;LOC101116286;ND4;novel.586;ND2;novel.4919;COX2;LOC101119975;novel.5553;ND6;LOC443104;novel.585;novel.2366;ATP5G2;novel.583;ND3 | ITPR1;LOC101104348;DRD1;novel.4092;SLC39A8 |
| Amyotrophic lateral sclerosis | ATP8;MAPK13;CYTB;ND4L;COX3;COX1;novel.4917;ATP6;GRIN2D;LOC101116286;ND4;novel.586;ND2;novel.4919;COX2;LOC101119975;novel.5553;ND6;LOC443104;novel.585;novel.2366;ATP5G2;novel.583;ND3 | NUP133;GRIN2A;LOC101104348;LOC101104288;novel.4092 |
| Chemical carcinogenesis - reactive oxygen species | ATP8;MAPK13;CYTB;ND4L;COX3;COX1;novel.4917;ATP6;HMOX1;ND4;novel.586;LOC101111397;ND2;novel.4919;COX2;novel.5553;FOS;ND6;novel.585;novel.2366;ATP5G2;novel.583;novel.5497;ND3 | LOC101104348;VEGFA;LOC105616612;HGF |
| Thermogenesis | ATP8;MAPK13;CYTB;ND4L;COX3;COX1;novel.4917;ATP6;ND4;RPS6;novel.586;LOC101120042;ND2;novel.4919;COX2;novel.5553;ND6;novel.585;novel.2366;ATP5G2;novel.583;ND3 | ACSL3;ACSL1;LOC101104348;ARID1A;RPS6KA6;LOC105616612 |
| Diabetic cardiomyopathy | ATP8;COL1A2;MAPK13;CYTB;ND4L;COX3;COX1;novel.4917;ATP6;CAMK2A;COL1A1;ATP2A1;ND4;novel.586;ND2;novel.4919;COX2;novel.5553;ND6;novel.585;novel.2366;ATP5G2;novel.583;PRKCA;ND3 | PPP1CC;LOC101104348 |
| Huntington disease | ATP8;CYTB;ND4L;COX3;COX1;novel.4917;ATP6;LOC101116286;ND4;novel.586;ND2;novel.4919;COX2;LOC101119975;novel.5553;ND6;LOC443104;novel.585;novel.2366;ATP5G2;novel.583;ND3 | ITPR1;LOC101104348;RCOR1 |
| Calcium signaling pathway | VEGFB;CAMK2A;TNNC1;GRIN2D;ATP2A1;FGF1;CACNA1G;MYLK2;GRIN3B;ITPKA;PRKCA;CHRM1;ASPHD1 | SLC8A1;ITPR1;GRIN2A;ATP2B2;VEGFA;CACNA1D;DRD1;novel.4092;MRLN;HGF |
| Oxidative phosphorylation | ATP8;CYTB;ND4L;COX3;COX1;novel.4917;ATP6;ND4;novel.586;ND2;novel.4919;COX2;novel.5553;ND6;novel.585;novel.2366;ATP5G2;novel.583;ND3 | ATP6V0C;LOC101104348 |
| Focal adhesion | VEGFB;COL1A2;LOC106990244;COL1A1;MYLPF;MYL2;FAM101B;MYLK2;PRKCA | PPP1CC;ACTN4;RAPGEF1;XIAP;VAV3;VEGFA;COL6A3;LRCH1;LOC105616612;PAK7;HGF |
| Cardiac muscle contraction | TPM2;CYTB;COX3;COX1;novel.4917;TNNC1;ATP2A1;novel.4919;COX2;MYL2;novel.585;novel.583;ASPHD1 | SLC8A1;CACNA1D;CACNA2D1;CACNA2D2 |
| Retrograde endocannabinoid signaling | MAPK13;GNG11;ND4L;ND4;novel.586;ND2;novel.5553;ND6;novel.2366;PRKCA;ND3 | SLC17A6;ITPR1;LOC101104348;GABRA1;CACNA1D;novel.4092 |
| Adrenergic signaling in cardiomyocytes | TPM2;MAPK13;CAMK2A;TNNC1;ATP2A1;MYL2;PRKCA | SLC8A1;PPP1CC;ATP2B2;CACNA1D;CACNA2D1;SCN7A;CACNA2D2 |
| Hypertrophic cardiomyopathy | TPM2;TNNC1;ATP2A1;MYL2;TTN;DES | SLC8A1;CACNA1D;CACNA2D1;CACNA2D2 |
| Amphetamine addiction | CAMK2A;GRIN2D;FOS;GRIN3B;PRKCA | PPP1CC;GRIN2A;CACNA1D;DRD1 |
| Long-term potentiation | CAMK2A;GRIN2D;PRKCA | ITPR1;PPP1CC;GRIN2A;RPS6KA6;novel.4092 |

KEGG Enrichment Pathways and Differentially Expressed Genes in pituitary (IM vs.SN)

| KEGG Enrichment Pathways | Up Genes | Down Genes |
| --- | --- | --- |
| Pathways of neurodegeneration - multiple diseases | novel.4917;ND6;CYTB;ND5;ATP6;novel.2366;novel.5553;COX1;novel.4919;novel.584;COX2;CALML4;COX3;novel.586;novel.5551;ATP8;MAPK12;GRIN2D;ND2;LOC101103463;MAPK13;ND1;ATF4;LOC106991842;BCL2;novel.583;ND4;LOC105607070;AGER;ND4L;SNCA;ATP2A1;LOC101102327;novel.3117;SNCAIP;DNAH7;PRKCA;DNALI1;DNAH3;DNAL1;novel.495;novel.5257;DNAI1;LOC101108295;DNAH8;RYR2;LOC101106131 | DVL2;LOC106991402;LOC101104348;DERL1;ATXN2L;SLC25A4;ATF6;CSNK2A1;LOC101106728;DLG4;DVL1;KLC2;LOC101116843;TNFRSF1A;LOC101103959;RAB1A;ITPR1;LOC101121024;TRAF2;LOC105601936;NEFM;CACNA1C;NDUFS4;GRIN2A;ACTR1A;TARDBP;novel.4090;LOC101122774;CYBB;novel.1990;MAP2K1;novel.5729;novel.4091;novel.838;FZD8 |
| Coronavirus disease - COVID-19 | RPL4;RPL38;novel.5266;MAPK12;RPL26;RPL37;LOC106991767;RPL31;RPS25;RPS19;MAPK13;RPL22;LOC101123279;RPL5;RPL9;LOC101114033;novel.1337;RPS6;RPL3L;LOC101110998;RPL24;RPL30;LOC105604842;RPL32;novel.1700;RPS21;RPLP1;RPL39;LOC101103316;IKBKE;RPL35A;RPL28;RPS12;RPLP0;RPS262;C7;LOC101113831;RPS11;RPL8;LOC101116132;novel.4029;RPL29;RPL15;RPL102;RPS5;RPL18A;novel.435;RPL35;RPS29;RPS14;PRKCA;RPL10;RPS16;RPS2;RPS26;RPSA;RPL18;RPS27;LOC101110941;LOC106990113;LOC101107216;RPS15;LOC101102096;novel.4178;LOC101110758;novel.4101;novel.4488;LOC101105336;LOC105610139;LOC105613829;novel.58;novel.3542;LOC101107098;LOC101109545 | JUN;TNFRSF1A;MASP2;CYBB;ADAM17;RPL10A |
| Prion disease | novel.4917;ND6;CYTB;ND5;ATP6;novel.2366;novel.5553;COX1;novel.4919;novel.584;COX2;COX3;novel.586;novel.5551;ATP8;MAPK12;GRIN2D;ND2;MAPK13;ND1;ATF4;novel.583;ND4;LOC105607070;ND4L;LOC101102327;C7;NCF2;novel.495;LOC101108295;RYR2;LOC101106131;GRIN3B | LOC101104348;SLC25A4;PRKACA;CSNK2A1;LOC101114987;KLC2;LOC101116843;LOC101103959;ITPR1;LOC105601936;HSPA8;CACNA1C;NDUFS4;GRIN2A;novel.4090;LOC101122774;EGR1;CYBB;novel.4091;LOC101121082;novel.838 |
| Diabetic cardiomyopathy | novel.4917;ND6;CYTB;ND5;ATP6;novel.2366;novel.5553;COX1;novel.4919;novel.584;COX2;COX3;novel.586;novel.5551;ATP8;MAPK12;ND2;COL1A1;MAPK13;ND1;novel.583;ND4;LOC105607070;AGER;ND4L;COL1A2;ATP2A1;LOC101115115;PDK4;LOC101102327;LOC105609336;PRKCA;NCF2;LOC105613829;novel.495;RYR2;LOC101106131 | LOC101104348;SLC25A4;LOC106991605;PPP1CC;LOC101116843;NDUFS4;CYBB;novel.4319;novel.838 |
| Focal adhesion | FAM101B;VEGFB;BCAR1;COL1A1;PGF;THBS3;BCL2;LOC106990244;MYL2;COL1A2;CCND1;ZYX;PARVG;MYLPF;COL2A1;SHC3;PRKCA;MYLK2;COL9A3;TNXB;novel.5039;CCDC153;THBS2;LAMA4;COL9A2 | VEGFA;JUN;LRCH3;XIAP;ITGAV;LOC106991605;ACTN1;ACTN4;RAPGEF1;PPP1CC;PAK7;ITGA3;RELN;LOC101110859;VAV3;LRCH1;MAP2K1;novel.5593;LOC105615252 |
| Adrenergic signaling in cardiomyocytes | CALML4;TPM2;TPM4;MAPK12;MYH7;MAPK13;ATF4;BCL2;RAPGEF3;TNNC1;CACNG2;MYL2;PPP2R2C;ATP2A1;PPP1R1A;ADCY4;PIK3R6;PRKCA;RYR2 | ATP1B2;SLC9A1;ADRA1B;PRKACA;LOC106991605;ATP2B2;PPP1CC;ATP1A3;RPS6KA5;CACNA2D2;SCN4B;ADCY5;CACNA1C;CACNA2D1;LOC105606030;ATP1A1;SLC8A1;ADCY3;RAPGEFL1 |
| cGMP-PKG signaling pathway | CALML4;MYH7;ATF4;LOC105607070;ATP2A1;GNA12;MEF2B;ADCY4;PIK3R6;MRVI1;BDKRB2;LOC105609336;MYLK2;ADRA2C | KCNMA1;ATP1B2;ADRA1B;CNGA3;SLC25A4;LOC106991605;MEF2D;ATP2B2;PPP1CC;ATP1A3;GNA11;ITPR1;ADCY5;CACNA1C;novel.4090;LOC105606030;ATP1A1;SLC8A1;ADCY3;MAP2K1;GUCY1A2;LOC105601850;SRF;novel.4091 |
| Retrograde endocannabinoid signaling | ND6;ND5;novel.2366;novel.5553;novel.586;MAPK12;ND2;MAPK13;ND1;GNG11;GNG4;ND4;ND4L;LOC101106689;ADCY4;PRKCA | GABRR2;LOC101104348;SLC17A6;PRKACA;GABRA1;LOC101116843;ITPR1;KCNJ3;ADCY5;CACNA1C;NDUFS4;GNB5;SLC17A7;novel.4090;LOC101122774;LOC105606030;GABRA5;ADCY3;novel.4091;novel.838 |
| Apelin signaling pathway | CALML4;GNG11;MRAS;GNG4;RPS6;RRAS;MYL2;CCND1;MEF2B;LOC101106689;ADCY4;PIK3R6;LOC105609336;MYLK2;novel.5039;novel.3542;RYR2 | SLC9A1;PRKACA;LOC106991605;MEF2D;ITPR1;ADCY5;GNB5;novel.4090;HDAC4;LOC105606063;LOC105606030;EGR1;SLC8A1;ADCY3;LOC105616384;MAP2K1;novel.4091 |
| Oxidative phosphorylation | novel.4917;ND6;CYTB;ND5;ATP6;novel.2366;novel.5553;COX1;novel.4919;novel.584;COX2;COX3;novel.586;novel.5551;ATP8;ND2;ND1;novel.583;ND4;ND4L;ATP6AP1L;LOC101102327;novel.495;LOC101106131 | ATP6V0A1;LOC101104348;ATP6V0C;ATP6AP1;ATP6V0B;LOC101116843;ATP6V1A;NDUFS4;ATP6V1D;novel.838 |
| Lysosome | AP1G2;ATP6AP1L;LITAF;CLHC1 | ATP6V0A1;LAPTM4A;ATP6V0C;IGF2R;ATP6AP1;LAPTM4B;CTSV;ATP6V0B;LGMN;SLC17A5;M6PR;LOC101107188;CTSB;MFSD8;LOC105609990;LOC106991303;AP1M1;novel.3647;FUCA2;novel.4319;CTSO;LOC106991323;LOC105605995;MANBA;LAMP3;AP1M2 |
| Cardiac muscle contraction | novel.4917;CYTB;COX1;novel.4919;novel.584;COX2;COX3;TPM2;novel.5551;TPM4;MYH7;novel.583;TNNC1;CACNG2;MYL2;ATP2A1;LOC101102327;TRDN;RYR2;LOC101106131 | ATP1B2;ASPHD2;SLC9A1;ATP1A3;CACNA2D2;CACNA1C;CACNA2D1;ATP1A1;SLC8A1 |
| Glutamatergic synapse | GRIN2D;GNG11;GNG4;LOC101106689;ADCY4;GLUL;PRKCA;PLD1;GRIN3B | GRM6;SLC17A6;PRKACA;DLG4;GLS;ITPR1;KCNJ3;ADCY5;CACNA1C;SHANK2;GNB5;SLC1A2;GRIN2A;SLC17A7;novel.4090;LOC105606030;ADCY3;novel.4091 |
| Cholinergic synapse | ATF4;GNG11;GNG4;ACHE;BCL2;LOC101106689;ADCY4;PIK3R6;PRKCA | PRKACA;LOC106991605;GNA11;ITPR1;KCNJ14;KCNJ3;ADCY5;CACNA1C;GNB5;KCNQ4;novel.4090;LOC101122774;CHRNB2;LOC105606030;ADCY3;MAP2K1;novel.4091 |
| Parathyroid hormone synthesis, secretion and action | PTH1R;ATF4;novel.880;BCL2;GNA12;ADCY4;PRKCA;LOC101108953;PLD1;GATA3;novel.4455 | LOC101117505;PRKACA;MEF2D;MMP24;MMP15;GNA11;ITPR1;ADCY5;CDKN1A;novel.4090;LOC105606030;EGR1;ADCY3;MAP2K1;novel.4091 |
| Glycerophospholipid metabolism | DGKQ;LCAT;PROCA1;PNPLA6;CHKB;ACHE;PLA2G5;PLA2G2C;LOC101120283;PCYT1B;PLD1;PLPP4;DGKI | PLA2G12A;DGKD;AGPAT3;LPCAT2;EPT1;ETNK1;PISD;CDIPT;PTDSS2-2;LPCAT1;PLA1A;MBOAT1 |
| Synaptic vesicle cycle | CLHC1;LOC105616906 | STX3;ATP6V0A1;UNC13A;ATP6V0C;SLC17A6;ATP6V0B;SLC6A4;SLC6A11;AP2A1;SLC6A9;CPLX4;ATP6V1A;RAB3A;SLC1A2;SLC6A1;SLC17A7;LOC101121080;novel.3464;LOC101122774;SYT1;ATP6V1D;SLC18A2 |
| Morphine addiction | GNG11;GNG4;LOC101106689;ADCY4;PDE1A;PRKCA;LOC101108953 | GABRR2;LOC101117505;PRKACA;GABRA1;KCNJ3;DRD1;ADCY5;PDE10A;GNB5;LOC101122774;LOC105606030;GABRA5;ADCY3 |
| Mineral absorption | SLC26A6;LOC101117184;LOC101117691;FTL;FTH1;novel.1937;HEPHL1;novel.3155;novel.4175 | ATP1B2;ATP2B2;ATP1A3;TRPM7;SLC30A1;ATP1A1;SLC8A1;novel.652 |
| Ribosome | novel.5586;RPL4;RPL38;novel.5266;RPL26;RPL37;LOC106991767;RPL31;RPS25;RPS19;MRPS2;RPL22;LOC101123279;RPL5;RPL9;LOC101114033;novel.1337;RPS6;RPL3L;LOC101110998;RPL24;RPL30;LOC105604842;RPL32;novel.1700;RPS21;RPLP1;RPL39;LOC101103316;RPL35A;RPL28;RPS12;RPLP0;RPS262;RPS11;RPL8;LOC101116132;novel.4029;RPL29;RPL15;RPL102;RPS5;RPL18A;novel.435;RPL35;RPS29;RPS14;RPL10;RPS16;RPS2;RPS26;RPSA;RPL18;RPS27;LOC101110941;LOC106990113;LOC101107216;RPS15;LOC101102096;novel.4178;LOC101110758;novel.4101;novel.4488;LOC101105336;LOC105610139;novel.58;novel.3542;LOC101107098;LOC101109545 | RPL10A |

KEGG Enrichment Pathways and Differentially Expressed Genes in pituitary (SN vs. CON)

| KEGG Enrichment Pathways | Up Genes | Down Genes |
| --- | --- | --- |
| Pathways of neurodegeneration - multiple diseases | ATXN2L;ACTR1A;PSMD3;TNFRSF1A;LOC101121024;TARDBP;LOC101103959;MAP2K1;UCHL1;LOC101109608;ATP5B;novel.1990;LOC101119975;LOC106991402;LOC105605934;DNAL4;FZD8;CHRM1;FZD7;WNT9A;LOC101109981;novel.5750 | ND6;ND5;novel.5553;novel.4917;ATP6;novel.4919;CYTB;novel.2366;COX2;ND1;MAPK12;novel.584;novel.586;COX1;COX3;ATP8;novel.5551;LOC101104288;ND2 |
| Alzheimer disease | LOC106991605;PSMD3;SLC39A13;TNFRSF1A;LOC101121024;LOC101103959;MAP2K1;LOC101109608;ATP5B;LOC101119975;LOC106991402;LOC105605934;FZD8;CHRM1;FZD7;SLC39A4;WNT9A;LOC101109981;novel.5750 | ND6;ND5;novel.5553;novel.4917;ATP6;novel.4919;CYTB;novel.2366;COX2;ND1;novel.584;novel.586;COX1;COX3;ATP8;novel.5551;EIF2AK2;ND2 |
| Amyotrophic lateral sclerosis | ATXN2L;ACTR1A;PSMD3;TNFRSF1A;TARDBP;LOC101103959;ALYREF;LOC101109608;ATP5B;SLC1A2;novel.1990;LOC101119975;POM121C;DNAL4;novel.5750 | ND6;ND5;novel.5553;novel.4917;ATP6;novel.4919;CYTB;novel.2366;COX2;novel.3832;ND1;MAPK12;novel.584;novel.586;COX1;COX3;ATP8;novel.5551;LOC101104288;ND2 |
| Parkinson disease | PRKACA;PSMD3;SLC39A13;LOC101121024;LOC101103959;UCHL1;LOC101109608;ATP5B;LOC101119975;ADCY5;LOC106991402;SLC39A4;LOC101109981;novel.5750 | ND6;ND5;novel.5553;novel.4917;ATP6;novel.4919;CYTB;novel.2366;COX2;ND1;novel.584;novel.586;COX1;COX3;ATP8;novel.5551;ND2 |
| Prion disease | PRKACA;PSMD3;HSPA8;LOC101103959;LOC101109608;ATP5B;LOC494436;LOC101119975;EGR1;LOC101121082;LOC101109981;novel.5750 | ND6;ND5;novel.5553;novel.4917;ATP6;novel.4919;CYTB;novel.2366;COX2;ND1;MAPK12;novel.584;novel.586;COX1;COX3;ATP8;novel.5551;ND2 |
| Chemical carcinogenesis - reactive oxygen species | JUN;LOC106991605;MAP2K1;LOC101114408;AKR1A1;ATP5B;VEGFA;LOC101109981;LOC101115172 | ND6;ND5;novel.5553;novel.4917;ATP6;novel.4919;CYTB;novel.2366;COX2;LOC101119706;ND1;MAPK12;novel.584;novel.586;COX1;COX3;ATP8;novel.5551;ND2;HGF |
| Huntington disease | ACTR1A;PSMD3;AP2M1;LOC101103959;LOC101109608;ATP5B;SLC1A2;LOC101119975;DNAL4;LOC101109981;novel.5750 | ND6;ND5;novel.5553;novel.4917;ATP6;novel.4919;CYTB;novel.2366;COX2;ND1;novel.584;novel.586;COX1;COX3;ATP8;novel.5551;ND2 |
| Thermogenesis | PRKACA;ATP5B;ADCY5;LOC101105425 | ND6;ND5;novel.5553;novel.4917;ATP6;novel.4919;CYTB;novel.2366;COX2;ND1;MAPK12;novel.584;LOC106991149;novel.586;COX1;COX3;ATP8;novel.5551;novel.3542;ND2 |
| Diabetic cardiomyopathy | LOC106991605;ATP5B;MMP2;LOC101109981 | ND6;ND5;novel.5553;novel.4917;ATP6;novel.4919;CYTB;novel.2366;COX2;ND1;MAPK12;novel.584;novel.586;COX1;COX3;ATP8;novel.5551;LOC105613829;ND2 |
| Oxidative phosphorylation | ATP6AP1;ATP5B;ATP6V1D;LOC101105425 | ND6;ND5;novel.5553;novel.4917;ATP6;novel.4919;CYTB;novel.2366;COX2;ND1;novel.584;novel.586;COX1;COX3;ATP8;novel.5551;ND2 |
| Proteoglycans in cancer | CDKN1A;LOC106991605;PRKACA;SLC9A1;MAP2K1;VEGFA;TIMP3;LOC105605934;FZD8;MMP2;FZD7;LOC101114596;WNT9A | MAPK12;novel.3542;HGF |
| MicroRNAs in cancer | CDKN1A;PIM1;MAP2K1;SLC7A1;novel.363;HNRNPK;VEGFA;TIMP3;LOC105605934;SOX4;LOC101121082;LOC105616384 | LOC101119706;FAM101B;novel.1353 |
| Cardiac muscle contraction | ATP1B2;ASPHD2;SLC9A1;ATP1A3 | novel.4917;novel.4919;CYTB;COX2;novel.584;CACNG2;COX1;COX3;novel.5551;MYH7 |
| Retrograde endocannabinoid signaling | PRKACA;GABRR2;SLC17A7;GABRA1;ADCY5 | ND6;ND5;novel.5553;novel.2366;ND1;MAPK12;novel.586;ND2 |
| Non-alcoholic fatty liver disease | JUN;LOC106991605;TNFRSF1A | novel.4917;novel.4919;CYTB;COX2;MAPK12;novel.584;COX1;COX3;novel.5551;PKLR |
| Synaptic vesicle cycle | SLC6A11;CPLX4;VAMP2;UNC13A;SLC17A7;AP2M1;SLC6A1;ATP6V1D;SLC1A2;SYT1 | -- |
| Thyroid hormone signaling pathway | ATP1B2;LOC106991605;PRKACA;SLC9A1;ATP1A3;MAP2K1;LOC101121082;novel.2213 | BMP4;MYH7 |
| Bile secretion | ATP1B2;PRKACA;SLC9A1;ATP1A3;ADCY5;SLC10A1;LOC105605772;SLCO1A2;LOC101106534 | novel.1353 |
| Endocrine resistance | CDKN1A;JUN;LOC106991605;PRKACA;MAP2K1;ADCY5;LOC101121082;MMP2 | MAPK12 |
| Proximal tubule bicarbonate reclamation | ATP1B2;ATP1A3;SLC25A10;MDH1 | -- |
